# A New 3D 10-Connected Cd(II) Based MOF With Mixed Ligands: A Dual Photoluminescent Sensor for Nitroaroamatics and Ferric Ion

**DOI:** 10.3389/fchem.2019.00244

**Published:** 2019-04-16

**Authors:** Jun Wang, Jian Wu, Lu Lu, Hongjia Xu, Manoj Trivedi, Abhinav Kumar, Jianqiang Liu, Mingbin Zheng

**Affiliations:** ^1^School of Chemistry and Environmental Engineering, Sichuan University of Science and Engineering, Zigong, China; ^2^Guangxi Key Laboratory of Chemistry and Engineering of Forest Products, Guangxi University for Nationalities, College of Chemistry and Chemical Engineering, Nanning, China; ^3^Dongguan Key Laboratory of Drug Design and Formulation Technology, Key Laboratory of Research and Development of New Medical Materials of Guangdong Medical University, School of Pharmacy, Guangdong Medical University, Dongguan, China; ^4^Department of Chemistry, University of Delhi, New Delhi, India; ^5^Department of Chemistry, Faculty of Science, University of Lucknow, Lucknow, India

**Keywords:** MOF, nitroaromatics, sensor, theoretical calculation, topology

## Abstract

The precise unification of functional groups and photoluminescence properties can give rise to MOFs that can offer diverse applications like selective detection of nitroaromatic compounds (NACs) which are considered to be an important ingredient of explosive as well as cation and anion sensing. Hence, a new 3D metal-organic framework (MOF) [Cd_2_(btc)(bib)(HCOO)(H_2_O)·H_2_O]_n_ (**1**) has been synthesized using mixed ligand strategy by solvothermal reaction of cadmium acetate with two ligands *viz*. 1,3,5-benzenetricarboxylic acid (H_3_btc) and 1,4-bis(2-methyl-imidazol-1-yl)butane (bib). The MOF **1** possesses highly 10-connected network which is based on {Cd_4_(btc)_2_(bib)_4_} molecular building block. The studies showed that **1** could be taken as the fluorescent sensor for sensitive recognition of NACs, in particular 2,4,6-trinitrophenol (TNP) with notable quenching (*K*_sv_ = 5.42 × 10^4^ M^−1^) and LOD of 1.77 ppm. Additionally, **1** also displayed selective sensing for Fe^3+^ ions with *K*_sv_ = 6.05 × 10^3^ M^− 1^ and LOD = 1.56 ppm. Also, this dual sensor displayed excellent reusability toward the detection of TNP and Fe^3+^ ion. Theoretical calculations have been performed to propose the probable mechanism for the sensing luminescence intensity. Calculations indicated that because of the charge transfer and weak interaction that is operating between NACs and MOF, the weakening in the photoluminescence intensity resulted.

## Introduction

Currently, tremendous amount of efforts have been devoted in developing new metal-organic frameworks (MOFs) which display photoluminescent properties and hence this class of luminescence materials can be in demand for solving the pollution problems and can offer potential application as luminescent sensors (Hua et al., 2015; Li et al., 2015; Shi et al., 2015; Wang et al., 2015, 2017; Liu et al., 2016a,b, 2017; Chen et al., 2017; Lu et al., 2017; Ma et al., 2017). To prepare useful and excellent luminescent MOF based sensors, the ligand-based strategy had been proposed in which the precise incorporation of functional groups in the π-conjugated organic ligands and their coordination with d^10^-metal ions deliver MOFs which can be used as luminescent materials (Ma et al., 2013; Zheng et al., 2014; Wang et al., 2016; Guo et al., 2017; Chen et al., 2018a,b; Shen et al., 2018). Up to now, numerous luminescent MOFs have been documented which had been utilized to detect metal ions and small organic compounds (Cui et al., 2012). Sun et al. had synthesized two Cd(II)-based MOFs which demonstrated selectivity to detect acetone (Yi et al., 2012). Chen et al. proposed that the choice of metal centers play a crucial role in molecular recognition by binding interactions of metal sites of the host MOF with guest molecules (Chen et al., 2007). Bu et al. reported a highly sensitive luminescent Cd(II)-based MOF that quenched at 100 ppm of TNP and display a high quenching efficiency of 92.5% (Tian et al., 2014). Thus, luminescence quenching based detection of compounds offers an alternative that is simple, sensitive, and convenient in nature. The possible mechanism associated with the sensing properties of these materials depend on monitoring of the transmission signals generated during interactions that are taking place between sensors (guest) and substrates (host) (Gole et al., 2011; Pramanik et al., 2011; Chen et al., 2013; Balamurugan et al., 2014).

The recent investigations have proved that MOFs, constructed from mixed organic ligands comprising of dicarboxylate and N-donor linkers display interesting dynamic properties (Zhan et al., 2014). Bearing these aspects in mind and in continuation to our efforts in the area of metal-organic frameworks (MOFs) (Li et al., 2016; Liu et al., 2016c; Jin et al., 2017), which were aimed toward the syntheses of multifunctional MOFs we have chosen a flexible 1,4-bis(2-methyl-imidazol-1-yl)butane (bib) ligand (Hou et al., 2014a,b; Shi et al., 2014), and a rigid 1,3,5-benzenetricarboxylic acid (H_3_btc) ligand as well as d^10^ transition-metal center (Cd^2+^) to develop new structures with potential applications as luminescence sensor. The choice of using this strategy has been based on the following considerations (Marin et al., 2006; Kent et al., 2010; Saini and Das, 2012; Son et al., 2013; Banerjee et al., 2014; Li et al., 2014; Park and Lee, 2015; Wen et al., 2015; Park et al., 2016; Das and Mandal, 2018): (1) the binding capacity between bib ligand and metal centers not only induces flexible and diverse structures but also facilitates the surface functionalities of the materials; (2) rigid ligand btc with aromatic rings could effectively favor intra-ligand interactions and induce luminescent character. Herein, we are presenting a new Cd(II) MOF having formula[Cd_2_(btc)(bib)(HCOO)(H_2_O)·H_2_O]_n_ (**1**) which have been utilized as dual photoluminescent sensor for the selective detection of nitroaromatics (NACs) especially TNP and ferric ions.

## Materials and Methods

The instrumental, X-ray crystallographic, and computational details are presented in the Supplementary Information.

### Synthesis of [Cd_2_(btc)(bib)(HCOO)(H_2_O)·H_2_O]_n_ (1)

A mixture of H_3_btc (0.10 mmol, 0.021 g), bib (0.10 mmol, 0.022 g), Cd(NO_3_)_2_·4H_2_O (0.15 mmol, 0.027 g) in 20 mL DMF/H_2_O mixture (v/v = 3:1) was stirred for 30 min. This mixture was then transferred in a 25-mL Teflon-lined reactor, sealed and heated up to 120°C and this temperature was maintained for 72 h. The mixture was cooled down to room temperature with a cooling rate of 5°C/h. Yellow block type crystals of **1** were obtained in 74% yield on the basis of cadmium. Elemental analysis (% calc/found): C: 35.17/34.92, H:3.37/3.28, N:7.81/7.55.

## Results and Discussion

### Crystal Structure Description

**Figure 1:**
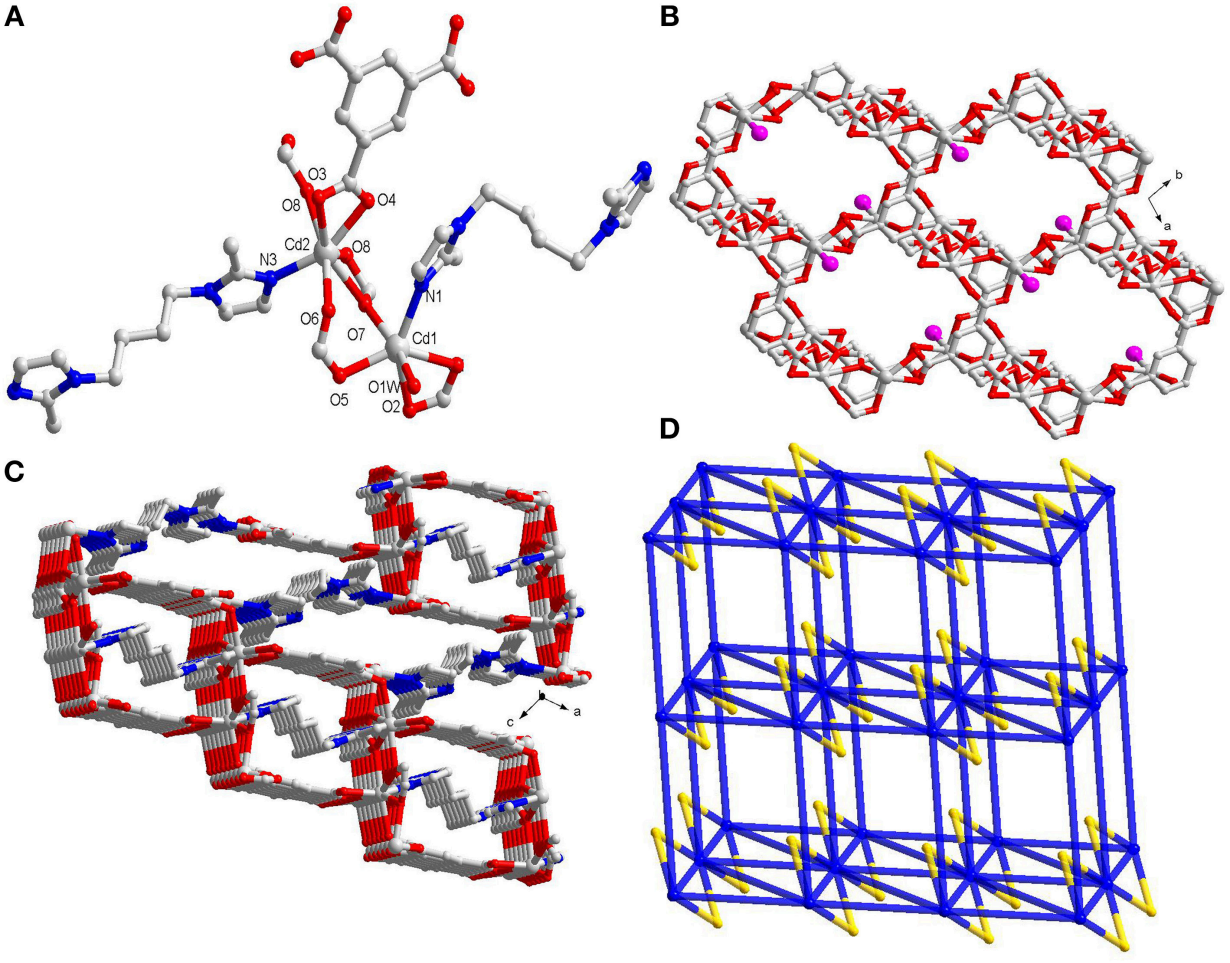
**(A)** The coordination environment around Cd(II) centers in **1**; **(B)** Perspective view of 2D layer constructed by Cd(II) centers and btc linkers(the pink ball represents the uncoordinated carboxylate group); **(C)** The 3D framework connected by btc and bib ligands; **(D)** Schematic representation of the 3D 10-connected network(the yellow ball represents the formate linker).

The single-crystal X-ray diffraction results indicate that **1** crystallizes in triclinic space group *P*-1. **1** is a 3D architecture having binuclear Cd(II) clusters as secondary building units(SBUs) which in turn is constructed by mixed ligands *viz*. btc and bib. **1** shows two types of Cd(II) centers with different coordination fashions. The SBUs of **1** are bonded by two bib, one μ_2_-η^2^:η^2^ formate anion and two bidentate chelating carboxylic anions and one bridging bidentate carboxylic anion (Figure 1A and Scheme S1). The formate anion was generated from DMF under acid condition. This conversion had already been reported previously many times (Lu et al., 2017). The Cd1 ion is hexa-coordinated where the CdO5N unit possess distorted octahedral geometry, while the Cd2 ion is seven-coordinated in which CdO6N moiety display distorted pentagonal bipyramidal geometry. The Cd1 coordinates to one carboxylate group from one btc, one O-atom of one bridged carboxylate from another btc, one μ_2_-O of one formate, one N from imidazole and is capped by the O of one H_2_O.The Cd2 center of **1** is connected to one carboxylate from one btc, one O of one bridged carboxylate from the other btc, three μ_2_-O of two formate anions and one N from imidazole. The dimeric units in SBU are formed by completely deprotonated btc anions to generate a 2D layered arrangement (Figure 1B and Figure S1). Further these 2D layers further linked by bib ligand to generate 3D framework (Figure 1C). Thus, the four Cd(II)centers are divulged by two bridged formates and two carboxylates of btc. Further it spreads out with six 3-connected btc and four linear imidazole-based bib ligands and in this way, this tetra-cadmium SBU can be simplified into a 10-connected node. The full motif of **1** can be taken as a 10-connected 3D network with (3^6^.4^4^.5^10^. 5^12^.6^8^.6^5^) topology (Figure 1D). The evacuated **1** shows theoretical porosity of 15.8% according to PLATON calculations with a probe radius of 1.65 Å (Spek, 2003). Thermogravimetric result indicates that **1** remains stable till 350°C (Figure S2). Also, PXRD experiment had been performed to assess whether the MOF is having phase purity in the solid state. The PXRD patterns confirm the phase purity of the bulk sample (Figure S3).

**Scheme 1:**
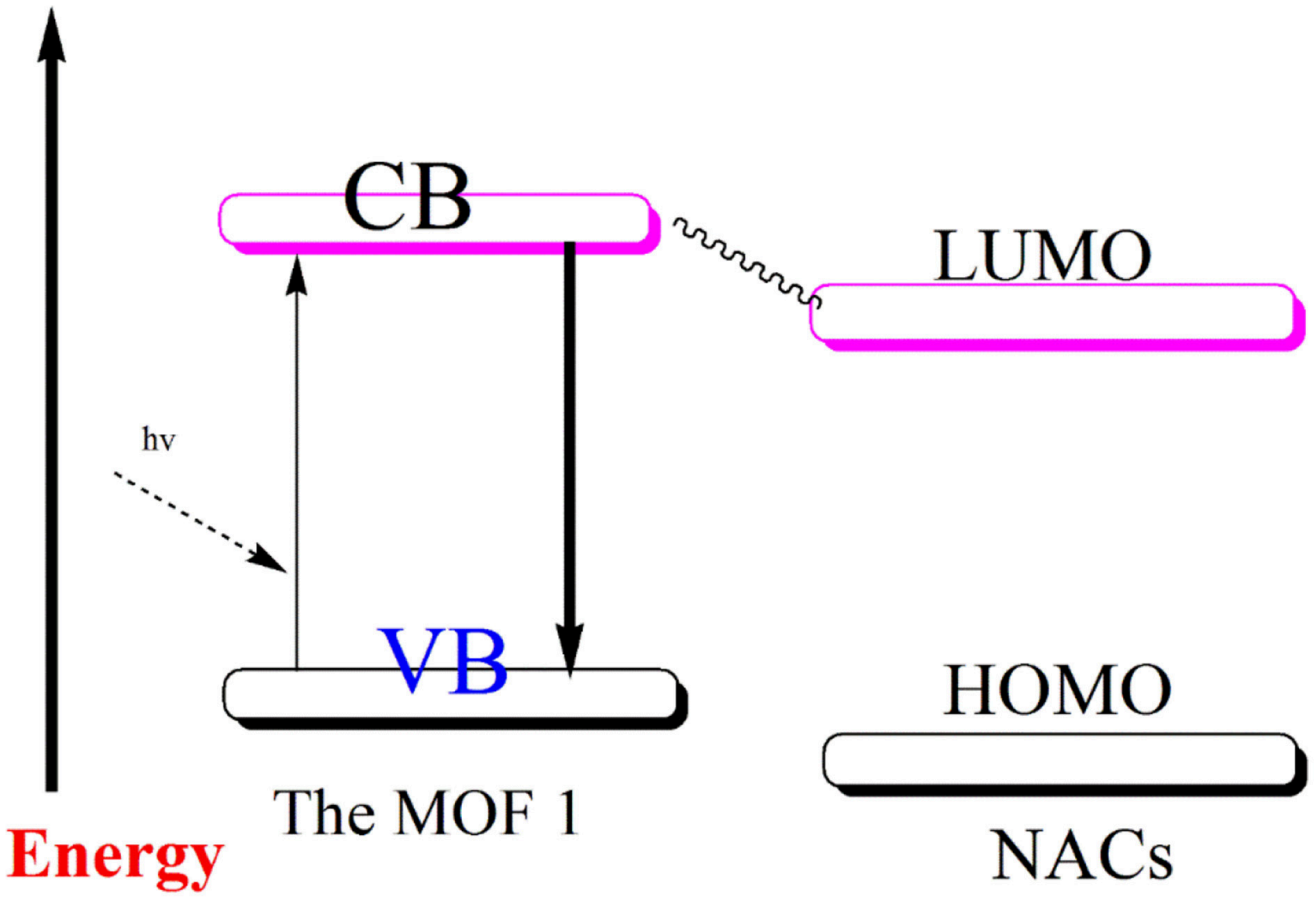
The schematic illustration of the electron transfer process between **1** and NACs.

### Luminescence Sensing

**Figure 2:**
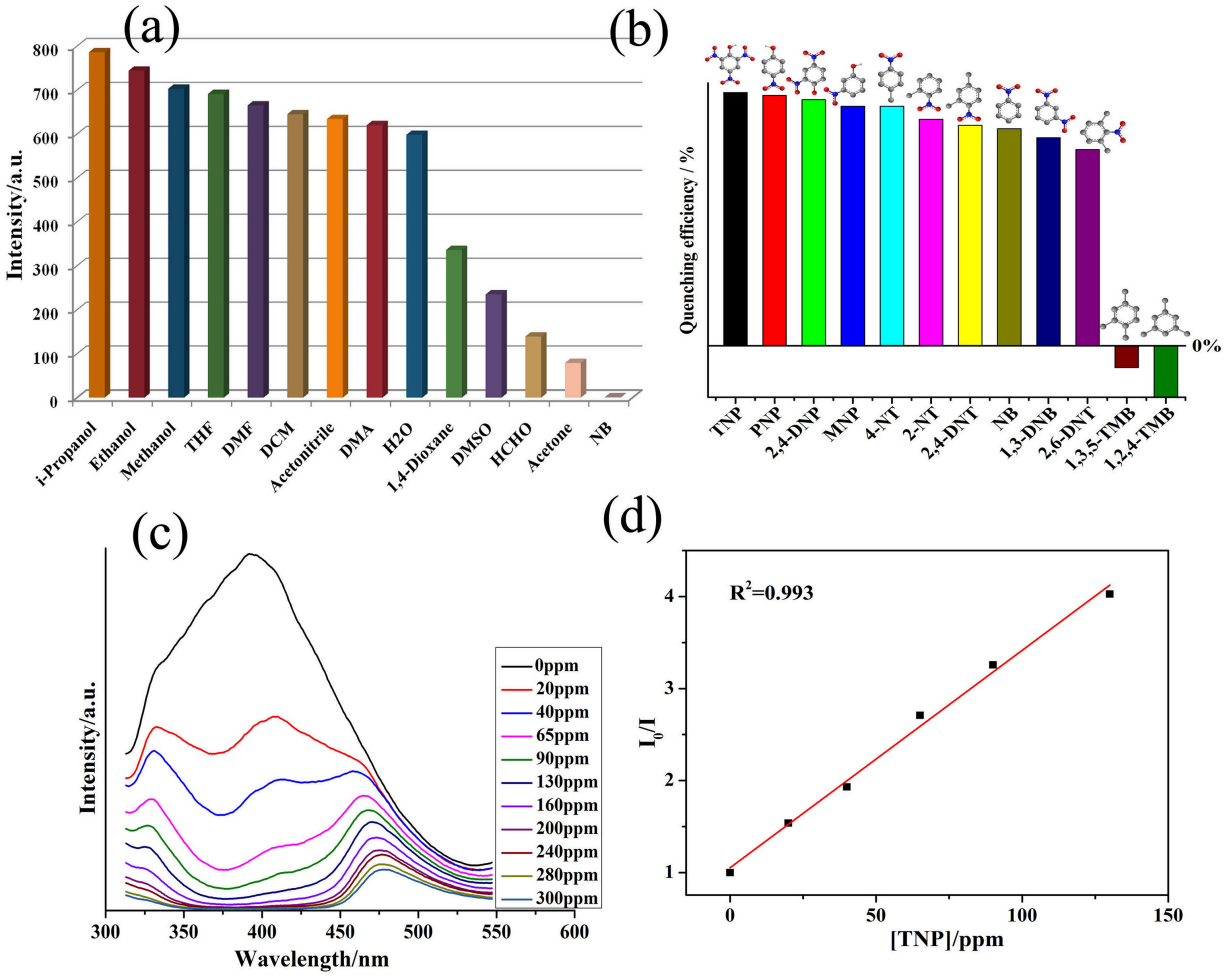
**(a)** The plots of varied luminescent intensity of **1** when dispersed in different small organic molecules (λ_ex_ = 320 nm); **(b)** The quenching efficiency of different nitro compounds (Emission intensities at 375 nm were selected); **(c)** The luminescence intensity of **1** recorded after addition of 1 mM TNP (λ_ex_ = 320 nm); **(d)** SV plot in the presence of **1** at different TNP concentrations.

In general, the MOFs based on d^10^-based metal centers and conjugated linkers are excellent candidates for the photoluminescent properties. It has been reported that the btc and bib exhibit emissions at 375 nm (λ_ex_ = 300 nm) and 445 nm (λ_ex_ = 370 nm), respectively (Li et al., 2015). **1** displayed emission with the maxima at 395 nm (λ_ex_ = 300 nm).The red-shift in emission maximum in the case of **1** as compared to btc with concomitant enhancement in the intensity indicates that the electron transfer may be taking place between the ligands and metalcenters (Allendorf et al., 2009; Rocha et al., 2011; Heine and Buschbaum, 2013; Hu et al., 2015). The spectral feature associated with **1** provided us impetus to use this MOF as the luminescent sensor for the detection of organic compounds (Figure S4). Thus, the photoluminescent property of the emulsion of **1** in different solvent was investigated. It has been found that luminescent spectrum of **1** is largely dependent on nature of small solvents, especially nitrobenzene (NB) (Figure S5) where **1** exhibits the most significant quenching effect in intensity (Figure 2a). The possible mechanism of the quenching in photoluminescent intensity in presence of NB has been proposed to originate from the electron-withdrawing effect from its nitro groups (Chen et al., 2009; Zheng M. et al., 2013; Song et al., 2014). Such solvent reliant luminescence properties can be interesting for sensing of different derivatives of NB. Hence, the luminescence intensity of emulsion of **1** in presence of different nitroaromatic compounds (NACs), *viz*.2,6-dinitrotoluene (2,6-DNT), *o*-nitrophenol (ONP),2,4-dinitrophenol (2,4-DNP), 2,4,6-trinitrophenol (TNP), *p*-nitrophenol (PNP),2-nitrotoluene (2-NT), 4-nitrotoluene (4-NT), 1,3-dinitrobenzene (1,3-DNB),1,2,4-trimethylbenzene 1,3,5-trimethylbenzene (1,3,5-TMB),2,4-dinitrotoluene (2,4-DNT), were recorded. The experiments showed that the addition of same concentration of NACs to the emulsion of **1** will results in the decline in its luminescent intensity to different levels (Figure 2b and Figures S6–S26). However, aromatic compounds not having such electron withdrawing nitro group, *viz.1,3,5*-TMB and 1,2,4-TMB have displayed intensity enhancement in **1**.

To further explore the sensing property of **1** toward TNP, the emission spectra of the suspension of **1** were recorded by gradual addition of batches of TNP solutions. The experiment indicated that the luminescence intensity of **1** declined with augmenting concentration of TNP (Figure 2c). The quenching efficiency was evaluated to be 44.5% for 20 ppm of TNP and 85% for 240 ppm of TNP. The Stern–Volmer (SV) equation which is used quantitatively to calculate the quenching efficiency was employed (Sanchez and Trogler, 2008; Sanchez et al., 2008; Zhang et al., 2015). The SV plot of TNP was almost linear at related low concentrations (Figure 2d), and its quenching constant (*K*_sv_) of **1** is 5.42 × 10^4^ M^−1^ and its LOD is 1.77 ppm, which was similar to some previously reported examples (Table S3). Additionally the *K*_sv_ value of **1** for TNP is 3.64 × 10^3^ M^−1^ and LOD parameter is 1.35 ppm. These observations clearly indicates that the **1** under study may be a highly selective sensor for TNP in comparison to other NACs (Qin et al., 2014; Singh and Nagaraja, 2014). The PXRD pattern of the recovered sample after performing 5 cycles of sensing studies indicate high stability of **1** and hence it can be concluded that the integrity of **1** remained almost unchanged after operational sensing works (Figure S3). On the basis of the structural feature of **1** which indicated only 15.8% porosity (*vide supra*), the encapsulation of TNP into the channels of MOF cannot take place. Thus, the interaction of analytes with **1** are more inclined toward the surface interaction operating between the analytes and **1**.

To offer the possible explanation for the decline in the photoluminescent intensity of MOF to varying magnitudes in the presence of different aromatic compounds, the computational investigation was done at the B3LYP level of theory (Table 1 and Figure S27). The weakening in photoluminescence intensity of MOFs when aromatic compounds comprising of –NO_2_ functions are added to them may be attributed to charge transfer that is taking place from conduction band of MOF to LUMO of these compounds (Scheme 1). To enable such transition the conduction band of MOFs should lie at higher energy scale than the conduction band of aromatic nitro-analytes (Lan et al., 2009; Zhang et al., 2010, 2011, 2016; Kreno et al., 2012; Li et al., 2013; Lin et al., 2016; Yang et al., 2016; Zhao et al., 2016, 2017). The Table 1 elucidatesthe conduction band energy levels of the full nitro-aromatic compounds, which are low than that of **1**.

**Table 1:** The HOMO-LUMO energies (in eV) for 1, ligand and aromatic analytes, ligand.

**Ligand/1/Analyte**	**HOMO**	**LUMO**
H_3_btc	−7.73	−1.97
**1**	−2.21	−1.13
2-nitrotoluene (2-NT)	−7.28	−2.32
4-nitrotoluene (4-NT)	−7.36	−2.32
Nitrobenzene (NB)	−7.60	−2.43
2, 6-dinitrotoluene (2,6-DNT)	−7.91	−2.87
2, 4-dinitrotoluene (2,4-DNT)	−8.11	−2.98
1, 3-dinitrobenzene (1,3-DNB)	−8.42	−3.14
2,4,6-trinitrophenol (TNP)	−8.54	−3.55
1,2,4-trimethylbenzene1,2,4-TMB	−6.03	0.28
1,3,5-trimethylbenzene1,3,5-TMB	−6.18	0.26
2,4-DNP	−7.62	−3.33
*o*-nitrophenol (ONP)	−6.80	−2.72
*p*-nitrophenol (PNP)	−7.43	−2.39

So, these nitro-aromatic analytes are in the apt position to accept the charge from **1** which is photo-excited. Hence, this phenomenon results in deterioration in photoluminescence intensity of the MOF to varying degree different nitro-aromatic compounds are added to it. Also, the Table 1 indicates that 1,3,5-TMB and 1,2,4-TMBare possessing higher LUMO energy level position than **1**. Therefore, these aromatic compounds deprived of –NO_2_ functions display small effect on photoluminescence intensity of **1**. However, the experimental order of decrement in photoluminescence intensity of **1** when different nitroaromatic analytes are added to it is not in accordance with their corresponding LUMO energy values. Therefore, it assessed that charge transfer may not be the solitary phenomenon responsible for decline in the photoluminescence. Additionally, along with the charge transfer phenomenon, weak interactions between the MOF and nitroaromatic analytes may also contribute toward the decline in the photoluminescence (Lan et al., 2009; Zhang et al., 2010, 2011; Kreno et al., 2012; Lin et al., 2016). Probably because of this reason the aromatic compounds 1,3,5-TMB and 1,2,4-TMB even though having relatively high LUMO energy values than **1** are slightly enhancing the photoluminescence intensity of **1** (Toal and Trogler, 2006; Kim et al., 2013; Wang et al., 2013; Zheng Q. et al., 2013; Hu et al., 2016).

Furthermore, to authenticate the selective sensing behavior of **1** toward TNP, the competitive experiments were executed by the adding different nitroaromatic compounds in the suspension of **1** followed by TNP (Figure S28). Results indicated that effective photoluminescent quenching was observed only when the TNP solution was added (Figure S28). These experiments validate the remarkable selectivity of **1** toward TNP. Further, to have better understanding into the selective TNP-sensing ability of **1**, the electronic properties of MOF reported herein as well as the nitroaromatic compounds were analyzed. The mechanism for photoluminescent quenching through electronic migration from conduction band of probes MOFs to LUMO of NAC shaving electron deficient nature is well-established (Toal and Trogler, 2006; Lan et al., 2009; Zhang et al., 2010, 2011; Kreno et al., 2012; Kim et al., 2013; Wang et al., 2013; Zheng M. et al., 2013; Hu et al., 2016; Lin et al., 2016). In general, the conduction band of rich electric probes MOFs is having higher energy in comparison to the energies of LUMO corresponding to nitro-aromatic compounds. With downfall in the LUMO energy values of NACs, the tendency to accept electron by these analytes and photoluminescent quenching becomes higher. The selective sensing for TNP is in agreement with its lower LUMO energy in comparison to other NACs (Table 1). The disagreements between quenching of photoluminescent intensity for others NACs except for TNP and their LUMO energy parametersare conducted, in which may contain the electronic migration and/or resonance energy transfer (RET).The correlative factors responsible for photoluminescent intensity quenching process (Hu et al., 2016). Also, the efficiency of energy transfer depend on the extent of overlap between the emission spectrum of MOF and the electronic absorption spectrum of the analyte (Marin et al., 2006; Kent et al., 2010; Saini and Das, 2012; Son et al., 2013; Banerjee et al., 2014; Li et al., 2014; Park and Lee, 2015; Wen et al., 2015; Park et al., 2016; Das and Mandal, 2018). In sharp contrast to other NACs the absorption spectrum for TNP displays good full coverage the emission spectrum of **1** (Figure S29). This clearly suggests the electronic migration and energy conversion mechanisms are associated with the luminescence quenching in **1** by TNP, but electron transfer mechanism solely exists for other nitro-aromatic compounds. The existence of the dominating energy transfer between **1** and TNP has also been substantiated by the preferential quenching of the 375 nm band over 475 nm during the titration experiments (Figure 2c). The emission band at ~375 nm display spectral overlap with the electronic absorption spectrum of TNP which lead to efficient quenching of this band by an energy transfer mechanism (Figure S29). Antagonistically, the emission band at ~475 nm display poor spectral overlap with the electronic absorption spectrum of TNP because of which the quenching of this emission band occurs by a “less efficient” photo-induced electron transfer (PET) mechanism (Banerjee et al., 2014). In addition, there might be electrostatic interactions between TNP and the nitrogen centers of the ligands in **1** (Marin et al., 2006; Kent et al., 2010; Saini and Das, 2012; Son et al., 2013; Banerjee et al., 2014; Li et al., 2014; Park and Lee, 2015; Wen et al., 2015; Park et al., 2016; Das and Mandal, 2018) Hence due to combination of electron-transfer, energy-transfer and electrostatic interaction between TNP **1**, the quenching efficiency for TNP gets significantly enhanced (Marin et al., 2006; Kent et al., 2010; Son et al., 2013; Li et al., 2014; Das and Mandal, 2018).

**Figure 3:**
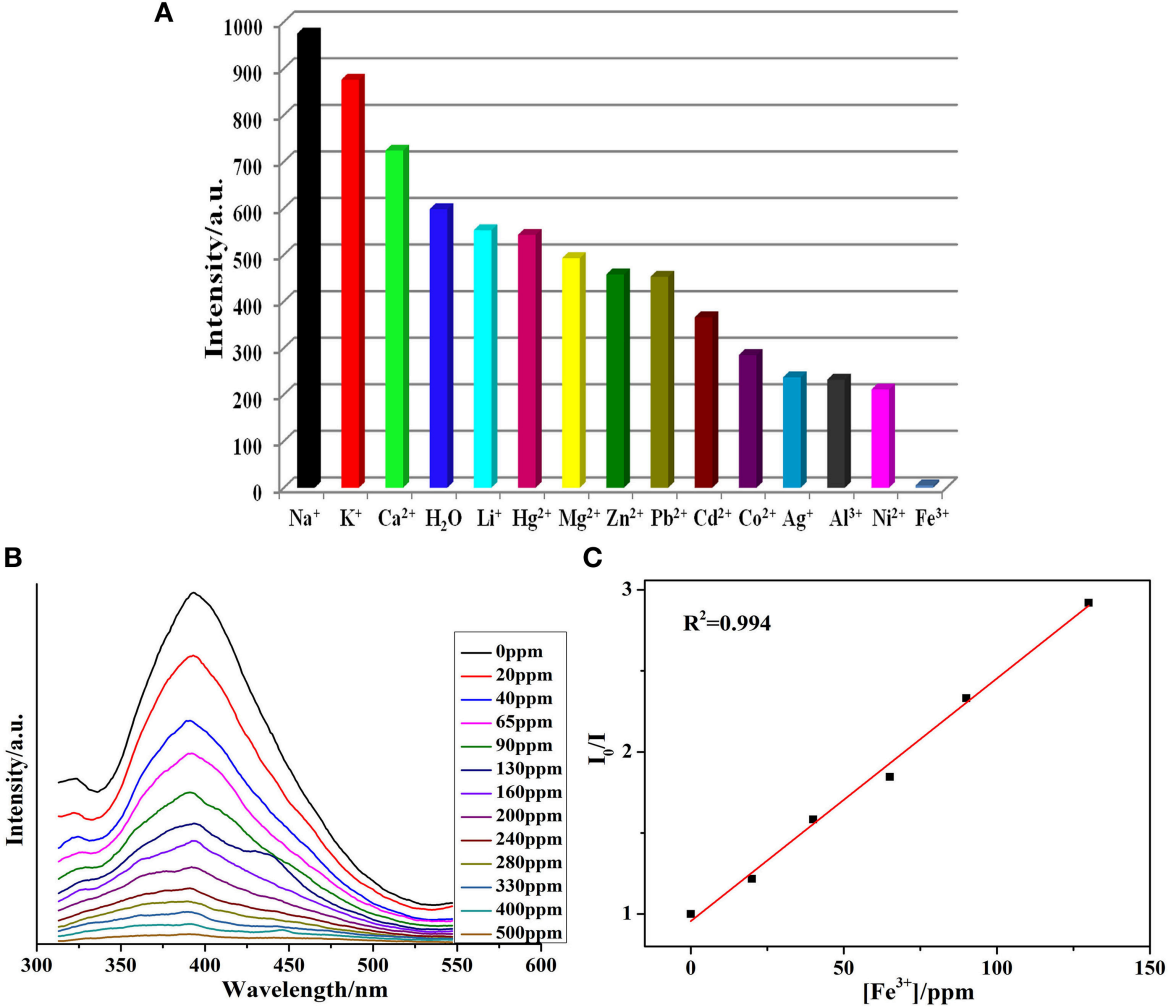
**(A)** Photoluminescence intensity of **1** when it was dispersed in different metal ions solutions in H_2_O (λ_ex_ = 320 nm); **(B)** Quenching in photoluminescence intensity after adding 1 mM Fe^3+^ solution; **(C)** SV plot after adding different Fe^3+^ concentrations.

The MOF**1** was suspended in distilled water having 1 × 10^2^ M M(NO_3_)_n_ to form the M^n+^@**1** to perform sensing experiments for the detection of metal cations. The intensities were enhanced upon adding Na^+^, K^+^, and Ca^2+^ ions in solutions when compared to the blank experiment (Figure 3A), while other metal ions exhibited different levels of quenching effects. Notably in the presence of Fe^3+^ ions the significant quenching in the luminescence intensity of MOF was observed. Thus, the relationship between the concentrations of Fe^3+^ ions upon the intensity of **1** was explored by varying the concentration of Fe^3+^ (Figure 3B). When the concentration of ferric ion in the emulsion of **1** was 500 ppm, then the luminescent intensity was completely disappeared. The mechanism pertaining to the quenching effect caused by Fe^3+^could be explicated on the basis of electronic migration operating from the organic ligands of the MOF which behave as donor toward the metal ions which act as acceptor (Wu et al., 2015). The crystal structure investigation indicates that in**1** the uncoordinated oxygens of the carboxylate groups of btc ligands can behave as potential electrons donors.

Based on the above results, **1** shows a highly selective to Fe^3+^. The photoluminescent intensity of **1** was nearly nullified at a Fe^3+^ concentration of 6.05 × 10^3^ M^−1^. Further, the LOD was calculated *ca*. 1.56 ppm, which is comparable to the reported examples (Table S4) (Xiang et al., 2010; Jayaramulu et al., 2012; Liu et al., 2014; Cao et al., 2015; Xu et al., 2015). Moreover, the PXRD patterns of **1** after accomplishing sensing experiments indicated that the MOF retain its structural integrity and hence can be reused as a probe to detect ferric ions (Figure S3). The highly selective sensing for Fe^3+^ ions stimulated us to further inspect the effect of the related metal ions on Fe^3+^ sensing. This had been checked by the addition of metal ions other than Fe^3+^ to **1** followed by Fe^3+^. The experiments revealed that the luminescence intensity of **1** will be completely quenched while the Fe^3+^ ion (10^−2^ M) is added to **1**. The *K*_sv_ of Fe^3+^ in the above systems is little greater that the value obtained for the pure **1** (Figure S30).

The possible sensing mechanism associated with the luminescence quenching of in the presence of Fe^3+^ has been further examined. Till date, some pertinent reasons for quenching in the luminescence intensity are: (Xu et al., 2011; Dang et al., 2012; Tang et al., 2013; Yang et al., 2013; Hu et al., 2014; Zhou et al., 2014; Xu and Yan, 2015) (I) the breakdown of framework; (II) ion exchange operating between sensing ions and the metal centers of probes; (III) resonance energy transfer (RET); and (IV) the weak interactions that are operating between metal cations and the functional groups from the organic ligands. The PXRD patterns (Figure S3) indicates that the MOF **1** even when immersed in metal ion solutions maintain its structural integrity. To substantiate the probable mechanism, the ICP has also been performed which showed that negligible Cd content is present in the filtrate obtained after suspending **1** in the Fe^3+^ solution for 1 day. This rules out that the exchange of Fe^3+^ with the Cd^2+^ of the MOF during sensing experiment (Table S5). Additionally, UV-Vis absorption data have been also conducted (Figure S31). The absorption spectrum of Fe^3+^ solution covers most of the emission spectrum of **1**, while in the absorption spectra of other metal ions no such spectral overlap have been observed. So, the competitive absorption of excitation wavelength between aqueous solution of Fe^3+^ and **1** may be plausible reason for the quenching in photoluminescnece (Tang et al., 2013; Yang et al., 2013; Hu et al., 2014). Furthermore, the fluorescence lifetime of 3.6 ms in **1** was reduced to 1.9 ms using 1.0 mM Fe^3+^ solution (Figure S32). Hence, energy transfer is the prime phenomenon because of which loss in photoluminescent intensity is observed. To further explicate the possible mechanism for luminescence quenching in **1** by Fe^3+^ ion, the X-ray photoelectron spectroscopy (XPS) on Fe^3+^@**1** showed that the energy of Fe 2p1 shifts to 712.6 eV, which may demonstrate the weak interaction between them (Figure S33) (Buragohain et al., 2016; Santra et al., 2016; Wu et al., 2017; Wang et al., 2018). In the time-dependent luminescence intensity measurement experiment (Figure S34), the final results indicated that it follows the first-order exponential decay curve. The character showed that the collision between Fe^3+^ and **1** may be faster than the static process. Hence, the static interaction might be incomplete instantly and acts as a continuous process which leads to a gradual decrease in luminescence intensity.

## Conclusion

A new 10-connected Cd(II)-based MOF with (3^6^.4^4^.5^10^.5^12^.6^8^.6^5^) topology have been synthesized. Moreover, **1** reveals the selective and sensitive photoluminescent quenching by TNP. A combined experimental and mechanistic investigation substantiates that the quenching of the photoluminescent intensity of MOF in presence of analytes can be credited to the simultaneous charge transfer and weak interactions that are operating between **1** and analytes. Additional investigation of **1** to detect metal-ions indicated extremely sensitive luminescent quenching of **1** framework by Fe^3+^ ion over other metal ions. The good quenching constant (6.05 × 10^3^ M^−1^) and very low limit of detection (LOD) (1.56 ppm), validates that **1** can be the potential detector for Fe^3+^. The excellent reusability/recyclability of MOF with respect to both TNP and Fe^3+^ sensing is certainly remarkable and this makes **1** as anew dual channel sensor for detection of TNP and Fe^3+^ ions.

## Author Contributions

JuW wrote the manuscript. JiW, LL, HX, and MT designed and synthesised the materials. AK, JL, and MZ checked the full materials.

### Conflict of Interest Statement

The authors declare that the research was conducted in the absence of any commercial or financial relationships that could be construed as a potential conflict of interest.

## References

[B1] Allendorf M. D. Bauer A. Bhakta R. K. Houk R. J. T. (2009). Luminescent metal-organic frameworks. Chem. Soc. Rev. 38, 1330–1352. 10.1039/B802352M 19384441

[B2] Balamurugan A. Kumar V. Jayakannan M. (2014). Triple action polymer probe: carboxylic distilbene fluorescent polymer chemosensor for temperature, metal-ions and biomolecules. Chem.Commun. 50, 842–845. 10.1039/C3CC45274C 24292277

[B3] Banerjee D. Hu Z. C. Li J. (2014). Luminescent metal-organic frameworks as explosive sensors. Dalton Trans. 43, 10668–10685. 10.1039/C4DT01196A 24921188

[B4] Buragohain A. Yousufuddin M. Sarma M. Biswas S. (2016). 3D luminescent amide-functionalized cadmium tetrazolate framework for selective detection of 2,4,6-trinitrophenol. Cryst. Growth Des. 16, 842–851. 10.1021/acs.cgd.5b01427

[B5] Cao K. L. Xia Y. Wang G. X. Feng Y. L. (2015). A robust luminescent Ba (II) metal-organic framework based on pyridine carboxylate ligand for sensing of small molecules. Inorg. Chem. Commun. 53, 42–45. 10.1016/j.inoche.2015.01.021

[B6] Chen B. Yang Y. Zapata F. Lin G. Qian G. Lobkovsky E. B. (2007). Luminescent open metal sites within a metal-organic framework for sensing small molecules. Adv. Mater. 19, 1693–1696. 10.1002/adma.200601838

[B7] Chen B. L. Wang L. B. Xiao Y. Q. Fronczek F. R. Xue M. Cui Y. J.. (2009). A luminescent metal-organic framework with lewis basic pyridyl sites for the sensing of metal ions. Angew. Chem. Int. Ed. 48, 508–511. 10.1002/ange.200805101 19072806

[B8] Chen W. M. Meng X. L. Zhuang G. L. Wang Z. Kurmoo M. Zhao Q. Q. (2017). A superior fluorescent sensor for Al^3+^and UO${}_{2}^{2+}$ based on a Co(II) metal–organic framework with exposed pyrimidyl Lewis base sites. J. Mater. Chem. A 5, 13079–13085. 10.1039/C7TA01546A

[B9] Chen Z. Mi X. Lu J. Wang S. Li Y. Dou J. (2018a). From 2D → 3D interpenetration to packing: N coligand-driven structural assembly and tuning of luminescent sensing activities towards Fe^3+^ and Cr_2_ O${}_{7}^{2-}$ ions. Dalton Trans. 47, 6240–6249. 10.1039/C8DT00909K 29687120

[B10] Chen Z. Mi X. Wang S. Lu J. Li Y. Li D. (2018b). Two novel penetrating coordination polymers based on flexible S-containing dicarboxylate acid with sensing properties towards Fe^3+^ and Cr_2_O${}_{7}^{2-}$ ions. J. Solid State Chem. 261, 75–85. 10.1016/j.jssc.2018.02.008

[B11] Chen Z. Sun Y. W. Zhang L. L. Sun D. Liu F. L. Meng Q. G.. (2013). A tubular europium-organic framework exhibiting selective sensing of Fe^3+^ and Al^3+^ over mixed metal ions. Chem. Commun. 49, 11557–11559. 10.1039/C3CC46613B 24177442

[B12] Cui Y. J. Yue Y. F. Qian G. D. Chen B. L. (2012). Luminescent functional metal–organic frameworks. Chem. Rev. 112, 1126–1162. 10.1021/cr200101d 21688849

[B13] Dang S. Ma E. Sun Z. M. Zhang H. J. (2012). A layer-structured Eu-MOF as a highly selective fluorescent probe for Fe^3+^ detection through a cation-exchange approach. J. Mater. Chem. 22, 16920–16926. 10.1039/C2JM32661B

[B14] Das P. Mandal S. K. (2018). Strategic design and functionalization of an amine-decorated luminescent metal organic framework for selective gas/vapor sorption and nanomolar sensing of 2,4,6-trinitrophenol in water. ACS Appl. Mater. Interfaces 10, 25360–25371. 10.1021/acsami.8b06339 29957936

[B15] Gole B. Bar A. K. Mukherjee S. (2011). Fluorescent metal–organic framework for selective sensing of nitroaromatic explosives. Chem. Commun. 47, 1137–12139. 10.1039/c1cc15594f 21993497

[B16] Guo L. Y. Su H. F. Kurmoo M. Wang X. P. Zhao Q. Q. Lin S. C.. (2017). Multifunctional triple-decker inverse 12-metallacrown-4 sandwiching halides. ACS Appl. Mater. Interfaces 9, 19980–19987. 10.1021/acsami.7b05191 28537067

[B17] Heine J. Buschbaum K. M. (2013). Engineering metal-based luminescence in coordination polymers and metal–organic frameworks. Chem. Soc. Rev. 42, 9232–9242. 10.1039/c3cs60232j 24077361

[B18] Hou Y. F. Liu B. Yue K. F. Zhou C. S. Wang Y. M. Yan N. (2014a). Five solvent-induced cadmium coordination polymers (CPs) based on the same mixed ligands. CrystEngComm 16, 9560–9567. 10.1039/C4CE01359J

[B19] Hou Y. F. Yan N. Yue K. F. Shi J. T. He T. Li X. Y. (2014b). Syntheses and characterization of Co(II) and Cu(II) coordination polymers (CPs) based on mixed flexible and rigid ligands. Inorg. Chem.Commun. 48, 44–47. 10.1016/j.inoche.2014.08.011

[B20] Hu X. L. Liu F. H. Qin C. Shao K. Z. Su Z. M. (2015). A 2D bilayered metal–organic framework as a fluorescent sensor for highly selective sensing of nitro explosives. Dalton. Trans. 44, 7822–7827. 10.1039/C5DT00515A 25820298

[B21] Hu Y. Ding M. Liu X. Sun L. Jiang H. (2016). Rational synthesis of an exceptionally stable Zn(II) metal-organic framework for the highly selective and sensitive detection of picric acid. Chem. Commun. 52, 5734–5737. 10.1039/C6CC01597B 27046028

[B22] Hu Z. C. Deibert B. J. Li J. (2014). Luminescent metal–organic frameworks for chemical sensing and explosive detection. Chem. Soc. Rev. 43, 5815–5840. 10.1039/C4CS00010B 24577142

[B23] Hua J. A. Zhao Y. Kang Y. S. Lu Y. Sun W. Y. (2015). Solvent-dependent zinc(II) coordination polymers with mixed ligands: selective sorption and fluorescence sensing. Dalton Trans. 44, 11524–11532. 10.1039/C5DT01386K 26032187

[B24] Jayaramulu K. Narayanan R. P. George S. J. Maji T. K. (2012). Luminescent microporous metal-organic framework with functional lewis basic sites on the pore surface: specific sensing and removal of metal ions. Inorg. Chem. 51, 10089–10091. 10.1021/ic3017547 22988809

[B25] Jin J. C. Wu X. R. Luo Z. D. Deng F. Y. Liu J. Q. Singh A. (2017). Luminescent sensing and photocatalytic degradation properties of an uncommon (4,5,5)-connected 3D MOF based on 3,5-di(3′,5′-dicarboxylphenyl)benzoic acid. CrystEngComm 19, 4368–4377. 10.1039/C7CE01012E

[B26] Kent C. A. Mehl B. P. Ma L. Papanikolas J. M. Meyer T. J. Lin W. (2010). Energy transfer dynamics in metal-organic frameworks. J. Am. Chem. Soc. 132, 12767–12769. 10.1021/ja102804s 20735124

[B27] Kim T. K. Lee J. H. Moon D. Moon H. R. (2013). Luminescent Li-based metal–organic framework tailored for the selective detection of explosive nitroaromatic compounds: direct observation of interaction sites. Inorg. Chem. 52, 589–595. 10.1021/ic3011458 23270469

[B28] Kreno L. E. Leong K. Farha O. K. Allendorf M. Van Duyne R. P. Hupp J. T. (2012). Metal-organic framework materials as chemical sensors. Chem. Rev. 112, 1105–1125. 10.1021/cr200324t 22070233

[B29] Lan A. Li K. Wu H. Olson D. H. Emge T. J. Ki Hong W.. (2009). A Luminescent Microporous Metal-Organic Framework for the Fast and Reversible Detection of High Explosives. Angew. Chem. Int. Ed. 48, 2370–2374. 10.1002/ange.200804853 19180622

[B30] Li B. H. Wu J. Liu J. Q. Gu C. Y. Xu J. W. Luo M. M. (2016). A luminescent zinc(II) metal-organic framework for selective detection of nitroaromatics, Fe^3+^ and CrO${}_{4}^{2-}$: a versatile threefold fluorescent sensor. ChemPlusChem 81, 885–892. 10.1002/cplu.201600304 31968818

[B31] Li J. Yang G. P. Hou L. Cui L. Li Y. P. Wang Y. Y.. (2013). Three new solvent-directed 3D lead(II)-MOFs displaying the unique properties of luminescence and selective CO_2_ sorption. Dalton Trans. 42, 13590–13598. 10.1039/c3dt51203g 23900684

[B32] Li X. Y. Liu M. Yue K. F. Wu Y. P. He T. Yan N. (2015). A series of reaction-controlled coordination polymers constructed from bis(imidazole) and tetrafluoroterephthalic acid ligands: syntheses, structural diversities, properties. CrystEngComm 17, 8273–8281. 10.1039/C5CE01423A

[B33] Li Y. Song H. Chen Q. Liu K. Zhao F. Y. Ruan W. J. (2014). Two coordination polymers with enhanced ligand-centered luminescence and assembly imparted sensing ability for acetone. J. Mater. Chem. A 2, 9469–9473. 10.1039/c4ta00944d

[B34] Lin R. B. Liu S. Y. Ye J. W. Li X. Y. Zhang J. P. (2016). Photoluminescent metal-organic frameworks for gas sensing. Adv. Sci. 3:1500434. 10.1002/advs.201500434 27818903 PMC5069648

[B35] Liu B. Wu W. P. Hou L. Wang Y. Y. (2014). Four uncommon nanocage-based Ln-MOFs: highly selective luminescent sensing for Cu^2+^ ions and selective CO_2_ capture. Chem. Commun. 50, 8731–8734. 10.1039/c4cc03049d 24963971

[B36] Liu J. Q. Li G. P. Liu W. C. Li Q. L. Li B. H. Gable R. W. (2016b). Two unusual nanocage-based Ln-MOFs with triazole sites: highly fluorescent sensing for Fe^3+^ and Cr_2_O${}_{7}^{2-}$, and selective CO_2_ capture. ChemPlusChem 81, 1299–1304. 10.1002/cplu.201600289 31964061

[B37] Liu J. Q. Liu G. L. Gu C. Y. Liu W. C. Xu J. W. Li B. H. (2016a). Rational synthesis of a novel 3,3,5-c polyhedral metal-organic framework with high thermal stability and hydrogen storage capability. J. Mater. Chem. A 4, 11630–11634. 10.1039/C6TA03675A

[B38] Liu J. Q. Wang W. J. Luo Z. D. Li B. H. Yuan D. Q. (2017). Microporous metal-organic framework based on ligand-truncation strategy with high performance for gas adsorption and separation. Inorg. Chem. 56, 10215–10219. 10.1021/acs.inorgchem.7b00851 28837334

[B39] Liu J. Q. Wu J. Li F. M. Liu W. C. Li B. H. Wang J. (2016c). Luminescent sensing from a new Zn(II) metal–organic framework. RSC. Adv. 6, 31161–31166. 10.1039/C6RA01709F

[B40] Lu L. Wu J. Wang J. Liu J. Q. Li B. H. Singh A. (2017). An uncommon 3D 3,3,4,8-c Cd(II) metal-organic framework for highly efficient luminescent sensing and organic dye adsorption: experimental and theoretical insight. CrystEngComm 19, 7057–7067. 10.1039/C7CE01638G

[B41] Ma D. X. Li B. Y. Zhou X. J. Zhou Q. Liu K. Zeng G.. (2013). A dual functional MOF as a luminescent sensor for quantitatively detecting the concentration of nitrobenzene and temperature. Chem. Commun. 49, 8964–8966. 10.1039/c3cc44546a 23963022

[B42] Ma R. Chen Z. Wang S. Yao Q. Li Y. Lu J. (2017). Solvent-induced assembly of two helical Eu(III) metal-organic frameworks and fluorescence sensing activities towards nitrobenzene and Cu^2+^ ions. J. Solid State Chem. 252, 142–151. 10.1016/j.jssc.2017.05.018

[B43] Marin V. Holder E. Hoogenboom R. Tekin E. Schubert U. S. (2006). Light-emitting iridium(III) and ruthenium(II) polypyridyl complexes containing quadruple hydrogen-bonding moieties. Dalton Trans. 13, 1636–1644. 10.1039/b513957k 16547538

[B44] Park I. H. Mulijanto C. E. Lee H. H. Kang Y. Lee E. Chanthapally A. (2016). Influence of interpenetration in diamondoid metal-organic frameworks on the photoreactivity and sensing properties. Cryst. Growth Des. 16, 2504–2508. 10.1021/acs.cgd.6b00354

[B45] Park S. Lee S. Y. (2015). Significant enhancement of curcumin photoluminescence by a photosensitizing organogel: an optical sensor for pyrrole detection. Sens. Actuators B 220, 318–325. 10.1016/j.snb.2015.05.078

[B46] Pramanik S. Zheng C. Zhang X. Emge T. J. Li J. (2011). New microporous metal-organic framework demonstrating unique selectivity for detection of high explosives and aromatic compounds. J. Am. Chem. Soc. 133, 4153–4155. 10.1021/ja106851d 21384862

[B47] Qin J. S. Bao S. J. Li P. Xie W. Du D. Y. Zhao L. (2014). A stable porous anionic metal-organic framework for luminescence sensing of Ln3+ ions and detection of nitrobenzene. Chem. Asian J. 9, 749–753. 10.1002/asia.201301531 24402738

[B48] Rocha J. Carlos L. D. Paz F. A. A. Ananias D. (2011). Luminescent multifunctional lanthanides-based metal-organic frameworks. Chem. Soc. Rev. 40, 926–940. 10.1039/C0CS00130A 21180775

[B49] Saini R. K. Das K. J. (2012). Picosecond spectral relaxation of curcumin excited state in a binary solvent mixture of toluene and methanol. Phys. Chem. B 116, 10357–10363. 10.1021/jp305447y 22866603

[B50] Sanchez J. C. Trogler W. C. (2008). Efficient blue-emitting silafluorene-fluorine-conjugated copolymers: selective turn-off/turn-on detection of explosives. *J. Mater*. Chem. 18, 3143–3156. 10.1039/B802623H

[B51] Sanchez J. C. Urbas S. A. Toal S. J. DiPasquale A. G. Rheingold A. L. Trogler W. C. (2008). Catalytic hydrosilylation routes to divinylbenzene bridged silole and silafluorene polymers. Applications to surface imaging of explosive particulates. Macromolecules 41, 1237–1245. 10.1021/ma702274c

[B52] Santra D. C. Bera M. K. Sukul P. K. Malik S. (2016). Charge- transfer-induced fluorescence quenching of anthracene derivatives and selective detection of picric acid. Chem. Eur. J. 22, 2012–2019. 10.1002/chem.201504126 26743445

[B53] Shen J. Wang Z. Sun D. Xia C. Yuan S. Sun P.. (2018). pH-responsive nanovesicles with enhanced emission co-assembled by Ag(I) nanoclusters and polyethyleneimine as a superior sensor for Al^3+^. ACS Appl. Mater. Interfaces 10, 3955–3963. 10.1021/acsami.7b16316 29319291

[B54] Shi B. B. Zhong Y. H. Guo L. L. Li G. (2015). Two dimethylphenyl imidazole dicarboxylate-based lanthanide metal-organic frameworks for luminescence sensing of benzaldehyde. Dalton Trans. 44, 4362–4369. 10.1039/C4DT03326D 25641054

[B55] Shi J. T. Yue K. F. Liu B. Zhou C. S. Liu Y. L. Fang Z. G. (2014). Two porous metal-organic frameworks (MOFs) based on mixed ligands: synthesis, structure and selective gas adsorption. CrystEngComm 16, 3097–3102. 10.1039/c3ce41557k

[B56] Singh D. Nagaraja C. M. (2014). A luminescent 3D interpenetrating metal–organic framework for highly selective sensing of nitrobenzene. Dalton Trans. 43, 17912–17915. 10.1039/C4DT02841D 25360887

[B57] Son H. J. Jin S. Patwardhan S. Wezenberg S. J. Jeong N. C. So M. Hupp, J. T.. (2013). Light-harvesting and ultrafast energy migration in porphyrin-based metal–organic frameworks. J. Am. Chem. Soc. 135, 862–869. 10.1021/ja310596a 23249338

[B58] Song X. Z. Song S. Y. Zhao S. N. Hao Z. M. Zhu M. Meng X. (2014). Single-crystal-to-single-crystal transformation of a europium(III) metal-organic framework producing a multi-responsive luminescent sensor. Adv. Funct. Mater. 24, 4034–4041. 10.1002/adfm.201303986

[B59] Spek A. L. (2003). Single-crystal structure validation with the program PLATON. J. Appl. Crystallogr. 36, 7–13. 10.1107/S0021889802022112

[B60] Tang Q. Liu S. X. Liu Y. W. Miao J. Li S. J. Zhang L.. (2013). Cation sensing by a luminescent metal-organic framework with multiple lewis basic sites. Inorg. Chem. 52, 2799–2801. 10.1021/ic400029p 23458844

[B61] Tian D. Li Y. Chen R. Y. Chang Z. Wang G. Y. Bu X. H. (2014). A luminescent metal-organic framework demonstrating ideal detection ability for nitroaromatic explosives. J. Mater. Chem. A 2, 1465–1470. 10.1039/C3TA13983B

[B62] Toal S. J. Trogler W. C. (2006). Polymer sensors for nitroaromatic explosives detection. J. Mater. Chem. 116, 2871–2883. 10.1039/b517953j

[B63] Wang G. Y. Yang L. L. Li Y. Song H. Ruan W. J. Chang Z.. (2013). A luminescent 2D coordination polymer for selective sensing of nitrobenzene. Dalton Trans. 42, 12865–12868. 10.1039/c3dt51450a 23903950

[B64] Wang J. Wu X. R. Liu J. Q. Li B. H. Singh A. Kumar A. (2017). An uncommon (5,5)-connected 3D metal organic material for selective and sensitive sensing of nitroaromatics and ferric ion: experimental studies and theoretical analysis. CrystEngComm 19, 3519–3525. 10.1039/C7CE00912G

[B65] Wang X. L. Luan J. Lin H. Y. Lu Q. L. Le M. Liu G. C. (2015). Metal(II)-Organic coordination polymers modulated by two isomeric semirigid Bis-Pyridyl-Bis-Amide ligands: structures, fluorescent sensing behavior, and selective photocatalysis. ChemPlusChem 79, 1691–1702. 10.1002/cplu.201402193

[B66] Wang X. P. Han L. L. Wang Z. Guo L. Y. Sun D. (2016). Microporous Cd(II) metal-organic framework as fluorescent sensor for nitroaromatic explosives at the sub-ppm level. J. Mol. Struct. 1107, 1–6. 10.1016/j.molstruc.2015.11.018

[B67] Wang X. S. Li L. Yuan D. Huang Q. Y. B. Cao R. (2018). Fast, highly selective and sensitive anionic metal-organic framework with nitrogen-rich sites fluorescent chemosensor for nitro explosives detection. *J*. Hazard Mater. 344, 283–290. 10.1016/j.jhazmat.2017.10.027 29055832

[B68] Wen L. L. Xu X. Y. Lv K. L. Huang Y. M. Zheng X. F. Zhou L. (2015). Metal-organic frameworks constructed from d-camphor acid: bifunctional properties related to luminescence sensing and liquid-phase separation. *ACS Appl. Mater*. Interfaces 7, 4449–4455. 10.1021/acsami.5b00160 25654262

[B69] Wu Y. L. Yang G. P. Zhou X. Li J. Ning Y. Wang Y. Y. (2015). Three new luminescent Cd(II)-MOFs by regulating the tetracarboxylate and auxiliary co-ligands, displaying high sensitivity for Fe^3+^ in aqueous solution. Dalton Trans. 44, 10385–10391. 10.1039/C5DT00492F 25974713

[B70] Wu Y. P. Xu G. W. Dong W. W. Zhao J. Li D. S. Zhang J.. (2017). Anionic lanthanide MOFs as a platform for iron-selective sensing, systematic color tuning, and efficient nanoparticle catalysis. Inorg. Chem. 56, 1402–1411. 10.1021/acs.inorgchem.6b02476 28072525

[B71] Xiang S. Zhou W. Zhang Z. Green M. A. Liu Y. Chen B. (2010). Open metal sites within isostructural metal–organic frameworks for differential recognition of acetylene and extraordinarily high acetylene storage capacity at room temperature. Angew. Chem. Int. Ed. 49, 4719–4722. 10.1002/ange.201000094 20491101

[B72] Xu H. Hu H. C. Cao C. S. Zhao B. (2015). Lanthanide organic framework as a regenerable luminescent probe for Fe^3+^. Inorg. Chem. 54, 4585–4587. 10.1021/acs.inorgchem.5b00113 25947239

[B73] Xu H. Liu F. Cui Y. Chen B. Qian G. (2011). A luminescent nanoscale metal-organic framework for sensing of nitroaromatic explosives. Chem. Commun. 47, 3153–3155. 10.1039/c0cc05166g 21271003

[B74] Xu X. Y. Yan B. (2015). Eu(III)-functionalized MIL-124 as fluorescent probe for highly selectively sensing ions and organic small molecules especially for Fe(III) and Fe(II). ACS Appl. Mater. Interfaces 7, 721–729. 10.1021/am5070409 25510710

[B75] Yang C. X. Ren H. B. Yan X. P. (2013). Fluorescent metal-organic framework MIL-53(Al) for highly selective and sensitive detection of Fe^3+^ in aqueous solution. Anal. Chem. 85, 7441–7446. 10.1021/ac401387z 23826852

[B76] Yang Y. J. Wang M. J. Zhang K. L. (2016). A novel photoluminescent Cd(II)–organic framework exhibiting rapid and efficient multi-responsive fluorescence sensing for trace amounts of Fe^3+^ ions and some NACs, especially for 4-nitroaniline and 2-methyl-4 –nitroaniline. J. Mater. Chem. C 4, 11404–11418. 10.1039/C6TC04195G

[B77] Yi F. Y. Yang W. T. Sun Z. M. (2012). Highly selective acetone fluorescent sensors based on microporous Cd(II) metal–organic frameworks. J. Mater. Chem. 22, 23201–23209. 10.1039/C2JM35273G

[B78] Zhan C. Ou S. Zou C. Zhao M. Wu C. D. (2014). A luminescent mixed-lanthanide-organic framework sensor for decoding different volatile organic molecules. Anal. Chem. 86, 6648–6653. 10.1021/ac5013442 24892790

[B79] Zhang C. Che Y. Zhang Z. Yang X. Zang L. (2011). Fluorescent nanoscale zinc(II)-carboxylate coordination polymers for explosive sensing. *Chem*. Commun. 47, 2336–2338. 10.1039/C0CC04836D 21165504

[B80] Zhang C. Sun L. Yan Y. Li J. Song X. Liu Y.. (2015). A luminescent cadmium metal-organic framework for sensing of nitroaromatic explosives. Dalton Trans. 44, 230–236. 10.1039/C4DT02227K 25372514

[B81] Zhang L. L. Kang Z. X. Xin X. L. Sun D. F. (2016). Metal-organic frameworks based luminescent materials for nitroaromatics sensing. CrystEngComm 18, 193–206. 10.1039/C5CE01917F

[B82] Zhang Z. Xiang S. Rao X. Zheng Q. Fronczek F. R. Qian G.. (2010). A rod packing microporous metal-organic framework with open metal sites for selective guest sorption and sensing of nitrobenzene. Chem. Commun. 46, 7205–7207. 10.1039/c0cc01236j 20737107

[B83] Zhao S. Ding J. G. Zheng T. R. Li K. Li B. L. Wu B. (2017). The 3D and 2D cadmium coordination polymers as luminescent sensors for detection of nitroaromatics. J. Lumin. 188, 356–364. 10.1016/j.jlumin.2017.04.044

[B84] Zhao S. Lv X. X. Shi L. L. Li B. L. Wu B. (2016). An unusual (4,4)-connected 3D porous cadmium metal-organic framework as a luminescent sensor for detection of nitrobenzene. RSC. Adv. 6, 56035–56041. 10.1039/C6RA10664A

[B85] Zheng M. Tan H. Q. Xie Z. G. Zhang L. G. Jing X. B. Sun Z. C. (2013). Fast response and high sensitivity europium metal organic framework fluorescent probe with chelating terpyridine sites for Fe^3+^. ACS Appl. Mater. Interfaces 5, 1078–1083. 10.1021/am302862k 23331406

[B86] Zheng Q. Yang F. Deng M. Ling Y. Liu X. Chen Z.. (2013). A porous metal–organic framework constructed from carboxylate–pyrazolate shared heptanuclear zinc clusters: synthesis, gas adsorption, and guest-dependent luminescent properties. Inorg. Chem. 52, 10368–10374. 10.1021/ic401092j 24004179

[B87] Zheng X. F. Zhou L. Huang Y. M. Wang C. G. Duan J. G. Wen L. L. (2014). A series of metal-organic frameworks based on 5-(4-pyridyl)-isophthalic acid: selective sorption and fluorescence sensing. J. Mater. Chem. A 2, 12413–12422. 10.1039/C4TA01900H

[B88] Zhou Y. Chen H. H. Yan B. (2014). An Eu^3+^ post-functionalized nanosized metal-organic framework for cation exchange-based Fe^3+^-sensing in an aqueous environment. *J. Mater. Chem*. A 2, 13691–13697. 10.1039/C4TA01297F

