# Supplementary material for: A New 3D 10-Connected Cd(II) Based MOF With Mixed Ligands: A Dual Photoluminescent Sensor for Nitroaroamatics and Ferric Ion
Source: Front Chem. 2019 Apr 16;7:244. doi: 10.3389/fchem.2019.00244 (PMC6476924; doi:10.3389/fchem.2019.00244)
Supplement: Supplementary file 1 [file Data_Sheet_1.docx]

***Supporting Information***

A new 3D 10-connected Cd(II) based MOF with mixed ligands: A dual photoluminescent sensor for nitroaroamatics and ferric ion

Materials and Method

General Considerations

All the chemicals were purchased from commercial sources and used without further purification. The powder X-ray diffraction (PXRD) data were collected on a Bruker D8 ADVANCE X-ray diffractometer that was equipped with Cu-Kα radiation (λ=1.5418 Å) at 50 kV, 20 mA with a scanning rate of 6°/min and a step size of 0.02°. The simulated powder X-ray diffraction patterns for **1** were obtained using Mercury 2.0 software. The FT-IR spectra as KBr pellet were recorded using a Nicolet Impact 750 FTIR in the range of 400-4000 cm^-1^. Thermogravimetric analysis (TGA) was performed under nitrogen atmosphere from room temperature to 650°C at a heating rate of 10°C min^-1^, using a SDT Q600 thermogravimetric analyzer.

X-ray Crystallography

The single crystal X-ray diffraction data collection were carried out on a Bruker SMART APEX diffractometer that was equipped with a graphite monochromated Mo-Κα radiation (λ = 0.71073 Å) by using an ω-scan technique. The intensities against the absorption effects were corrected by using SADABS. The structure was solved by direct method (SHLEXS-2014) and refined using the full-matrix least-squares procedure based on F^2^ (Shelxl-2014).^1^ All the hydrogen atoms were generated geometrically and refined isotropically using the riding model. All the non-hydrogen atoms were refined with anisotropic displacement parameters. The crystallographic details and selected bond dimensions for **1** are listed in Tables S1 and S2. CCDC number: 1839729. The topological network with the Schläfli symbol was analyzed by TOPOS 4.0.^2^

Computational Details

The plausible mechanism related with the decline in the luminescent intensity of **1** in presence of NACs and to ascertain the nature of highest occupied molecular orbitals (HOMO) and lowest unoccupied molecular orbital (LUMO) of different nitro-analytes, the ligand as well as **1**, density functional theory (DFT) calculations were performed. The geometry optimizations were performed using the B3LYP exchange-correlation functional.^3^ For all the atoms except Cd 6-31G** basis set was used while for Cd CEP-121G basis set was employed for geometry optimization. All the calculations were performed using Gaussian 09 programme.^4^

Photoluminescence measurements

The photoluminescence properties of **1** were investigated in H_2_O/DMF suspensions at room temperature using a RF-5301PC fluorophotometer. These suspensions were prepared by adding 5 mg of finely divided **1** into 3 mL of H_2_O/DMF and then ultrasonically agitating the mixture for 30 min before testing.

References:

1. G. M. Sheldrick, SHELXT-Integrated space-group and crystal-structure determination. Acta. Crystallogr. Sect. A: Found. Adv. 71 (2015) 3.

2. (a) V.A. Blatov, Struct. Chem. 23 (2012) 955; (b) E. V. Alexandrov, V. A. Blatov, A. V. Kochetkov, D. M. Proserpio, CrystEngComm, 13(2011) 3947.

3. (a) A. D. Becke, J. Chem. Phys. 98 (1993) 5648; (b) C. T. Lee, W. T. Yang, R. G. Parr, Phys. Rev. B: Condens. Matter Mater. Phys. 37 (1998) 785.

4. M. J. Frisch, G. W. Trucks, H. B. Schlegel, G. E. Scuseria, M. A. Robb, J. R. Cheeseman, J. A. Montgomery, J. T. Vreven, K. N. Kudin, J. C. Burant, J. M. Millam, S. S. Iyengar, J. Tomasi, V. Barone, B. Mennucci, M. Cossi, G. Scalmani, N. Rega, G. A. Petersson, H. Nakatsuji, M. Hada, M. Ehara, K. Toyota, R. Fukuda, J. Hasegawa, M. Ishida, T. Nakajima, Y. Honda, O. Kitao, H. Nakai, M. Klene, X. Li, J. E. Knox, H. P. Hratchian, J. B. Cross,V. Bakken, C. Adamo, J. Jaramillo, R. Gomperts, R. E. Stratmann, O. Yazyev, A. J. Austin, R. Cammi, C. Pomelli, J. W. Ochterski, P. Y. Ayala, K. Morokuma, G. A. Voth, P. Salvador, J. J. Dannenberg, V. G. Zakrzewski, S. Dapprich, A. D. Daniels, M. C. Strain, O. Farkas, D. K. Malick, A. D. Rabuck, K. Raghavachari, J. B. Foresman, J. V. Ortiz, Q. Cui, A. G. Baboul, S. Clifford, J. Cioslowski, B. B. Stefanov, G. Liu, A. Liashenko, P. Piskorz, I. Komaromi, R. L. Martin, D. J. Fox, T. Keith, M. A. Al-Laham, C. Y. Peng, A. Nanayakkara, M. Challacombe, P. M. W. Gill, B. Johnson, W. Chen, W. M. Wong, C. Gonzalez, J. A. Pople, Gaussian, Inc. Walling ford CT (2009).





Scheme S1 view of the different coordination modes of the coligands in this work.


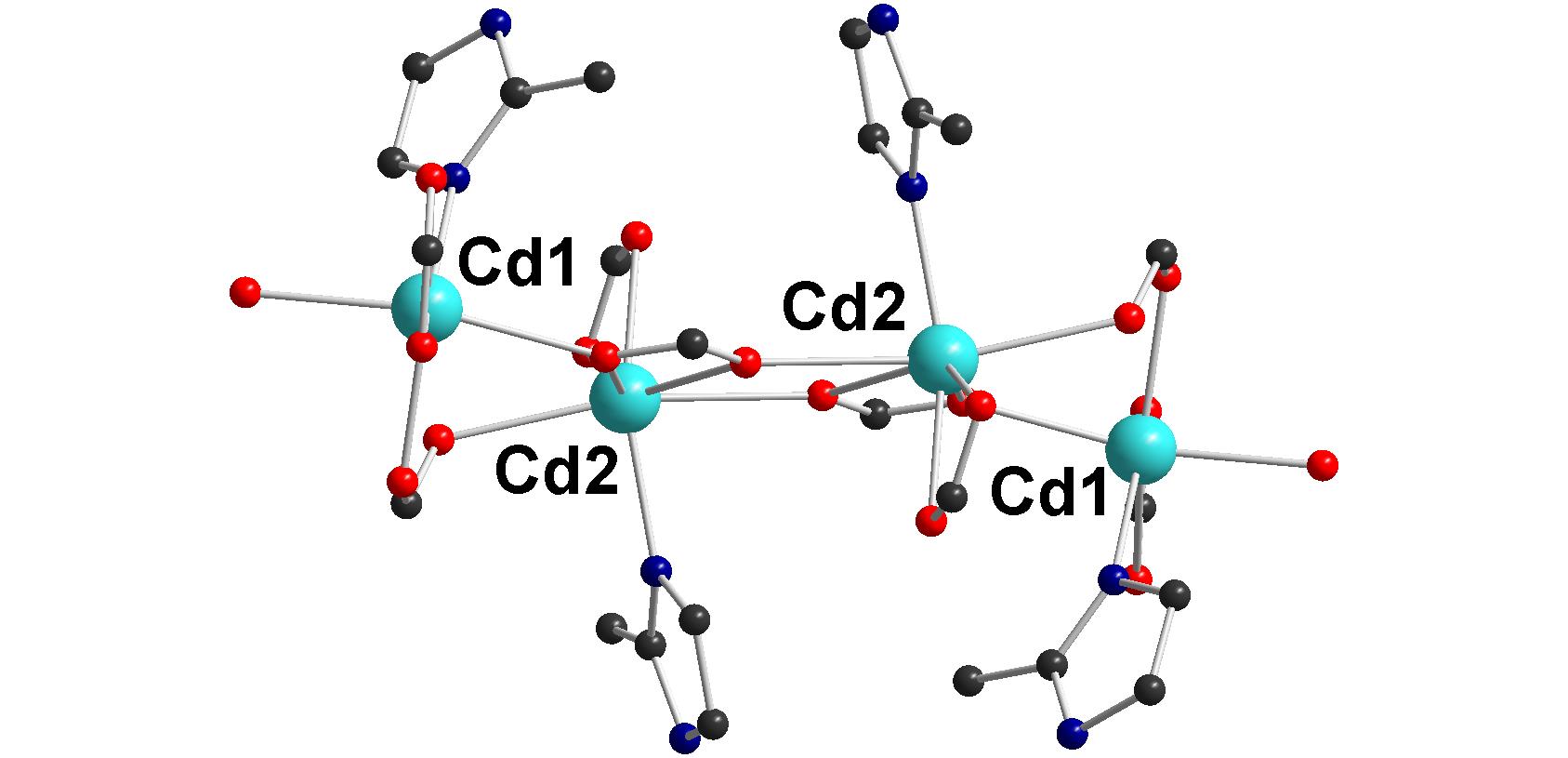


Figure S1 view of the tetracadimum SBU in **1**.


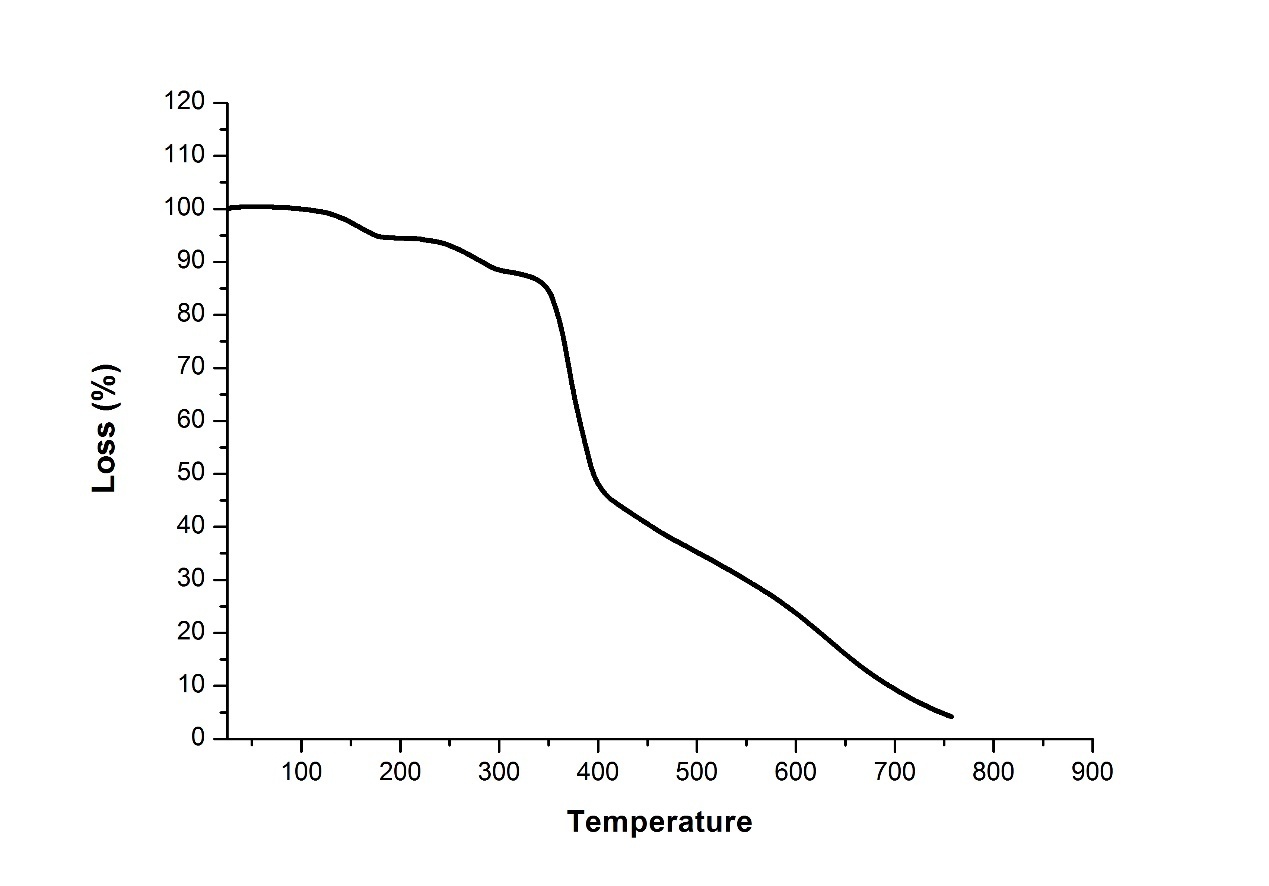


Fig. S2 view of the TGA.


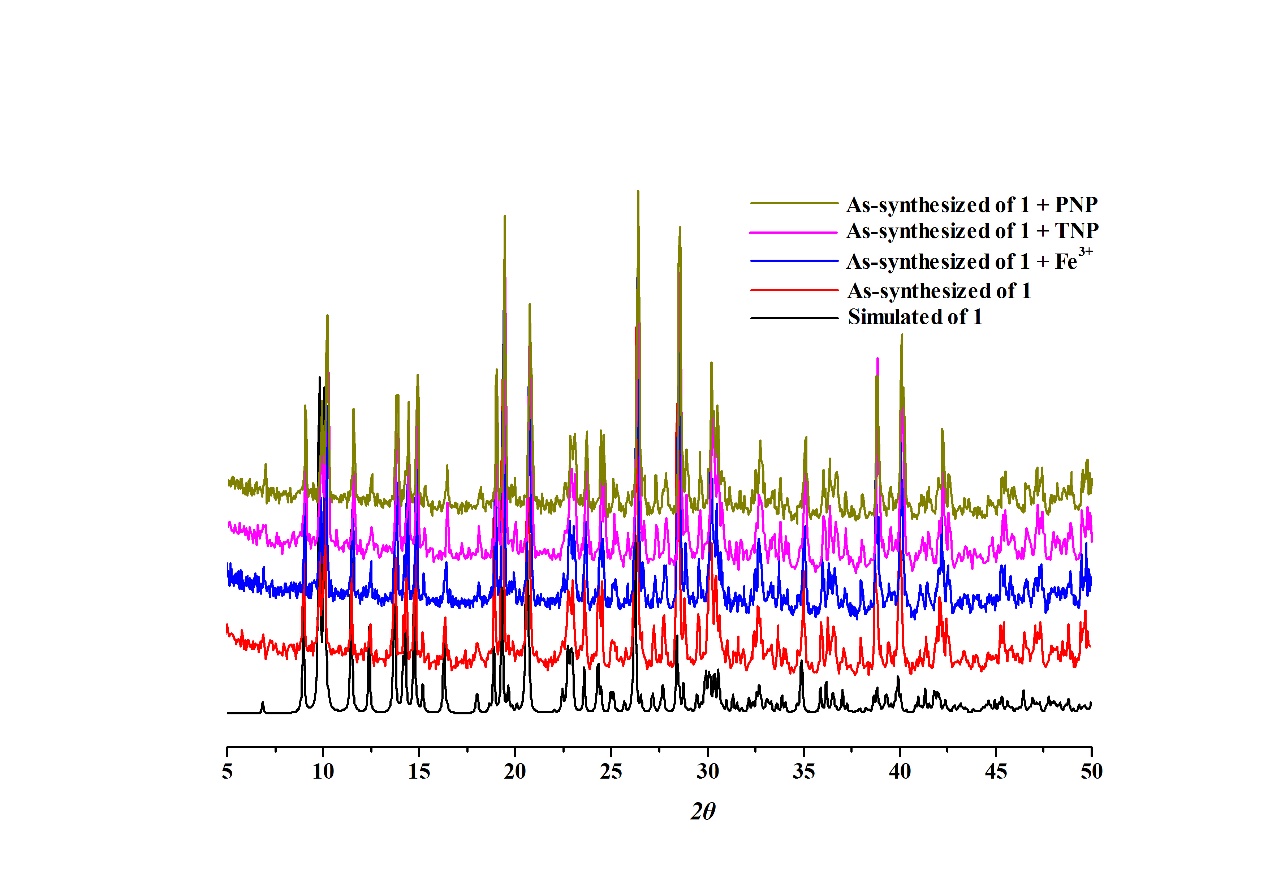
Fig. S3 Powder XRD profiles of **1**: simulated, as-synthesized and after being soaked in various analytes.


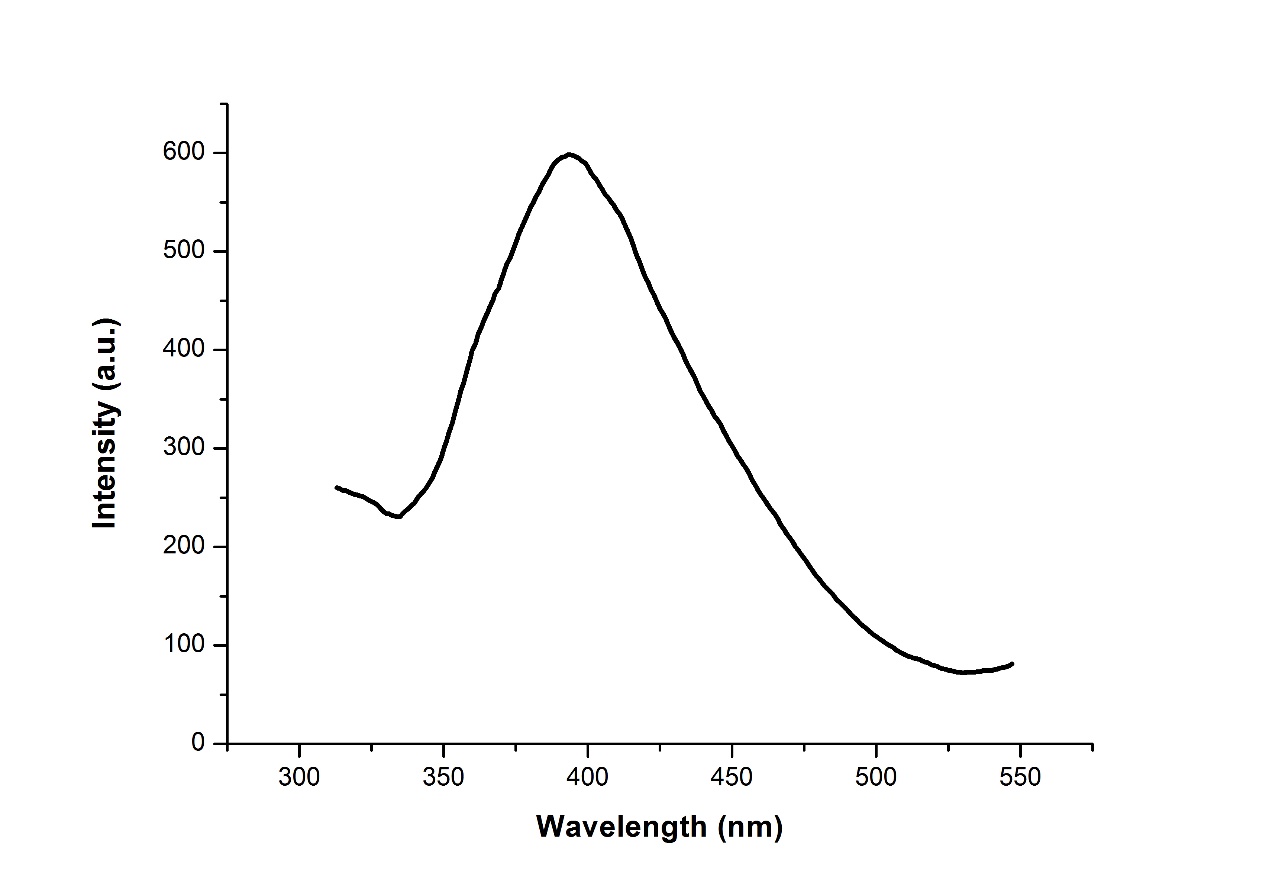


Fig. S4 the fluorescence emission of **1** at room temperature.


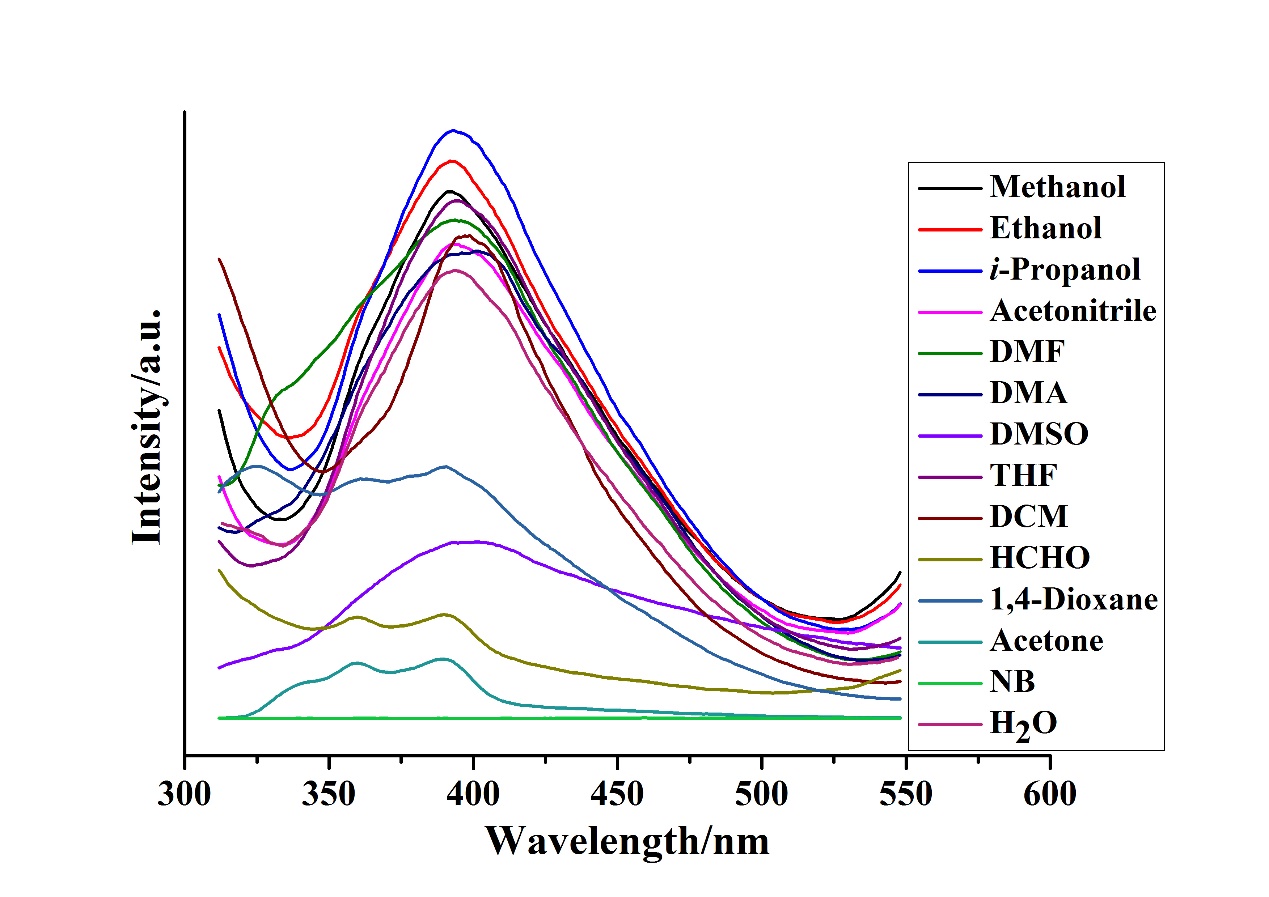


Fig. S5 view of the solvent-dependent luminescence intensity of different organic molecules.


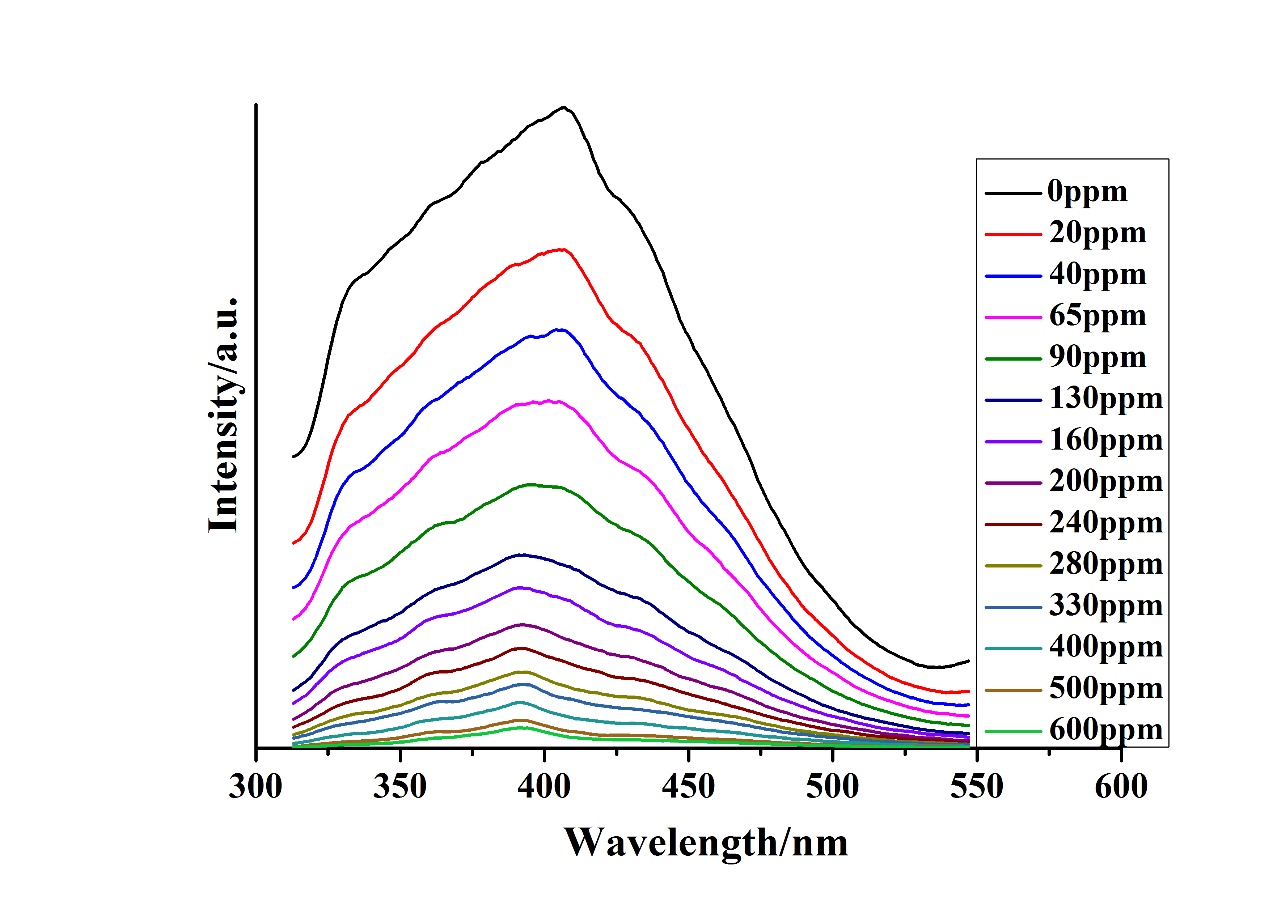


Fig. S6 Luminescent quenching of **1** dispersed in ethanol by the gradual addition of 1 mM solution of 1,3-DNB in DMF.


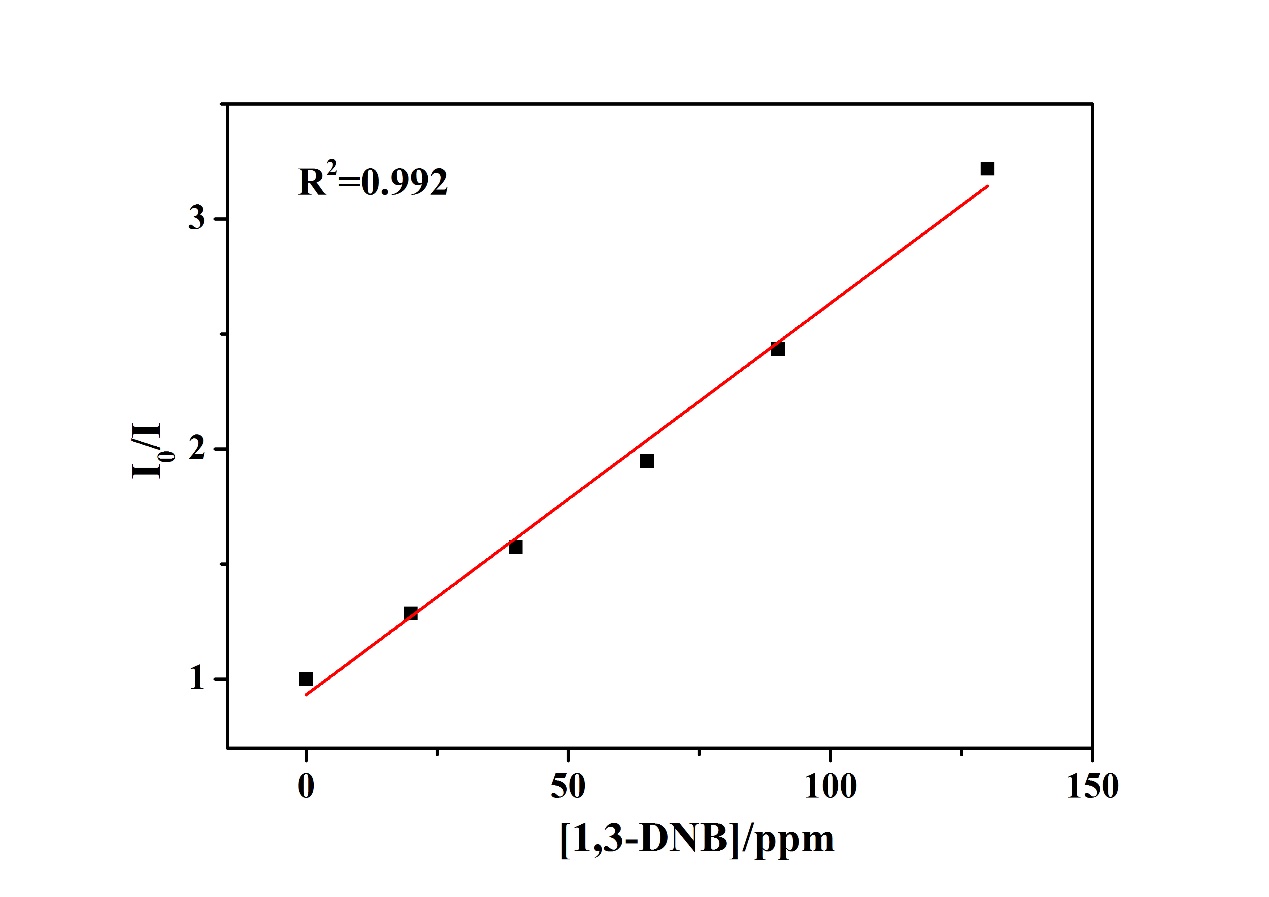


Fig. S7 The Stern–Volmer plot of **1** against 1,3-DNB.


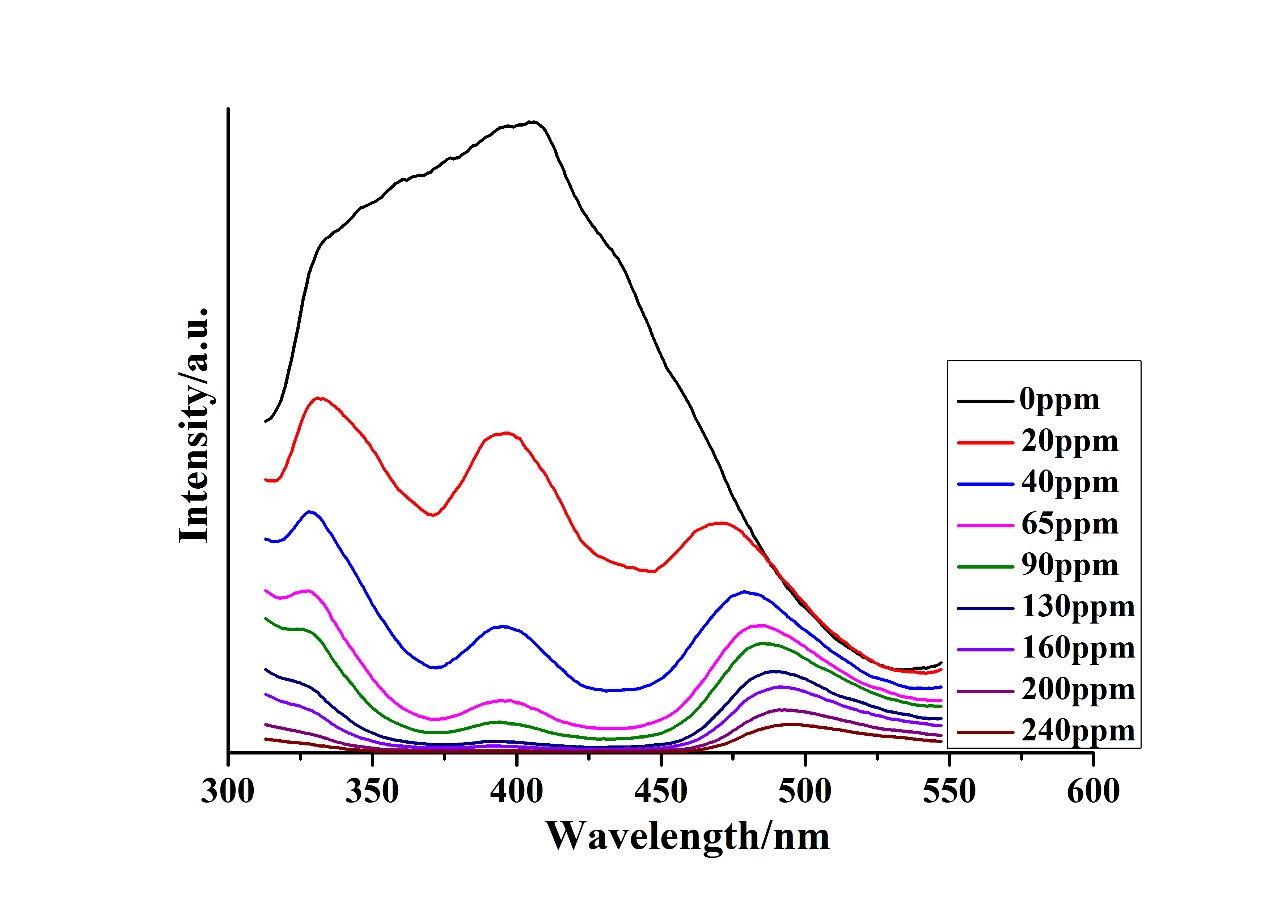


Fig. S8 Luminescent quenching of **1** dispersed in ethanol by the gradual addition of 1 mM solution of 2,4-DNP in DMF.


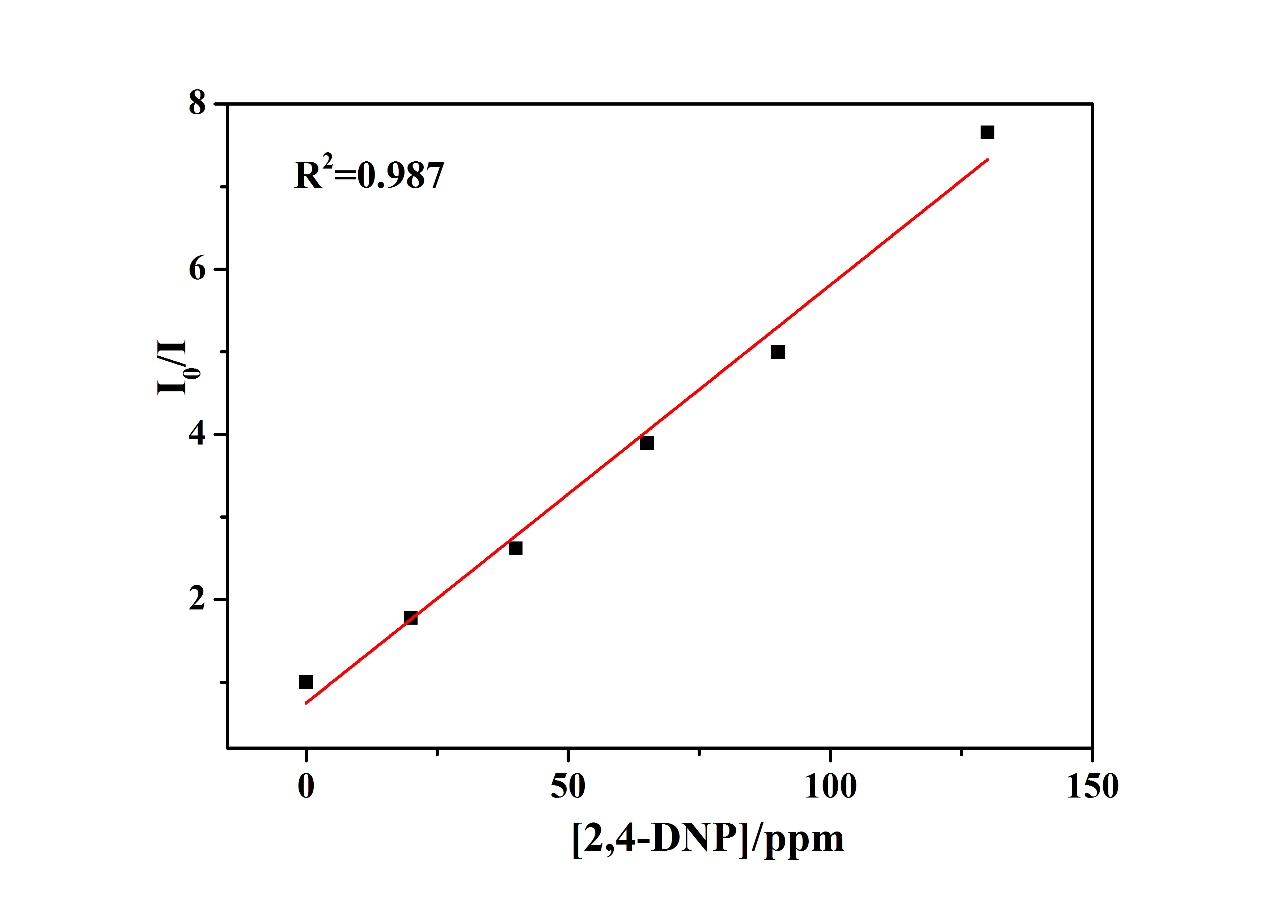


Fig. S9 Stern–Volmer plot for the fluorescence quenching of **1** upon the addition of 2,4-DNP.


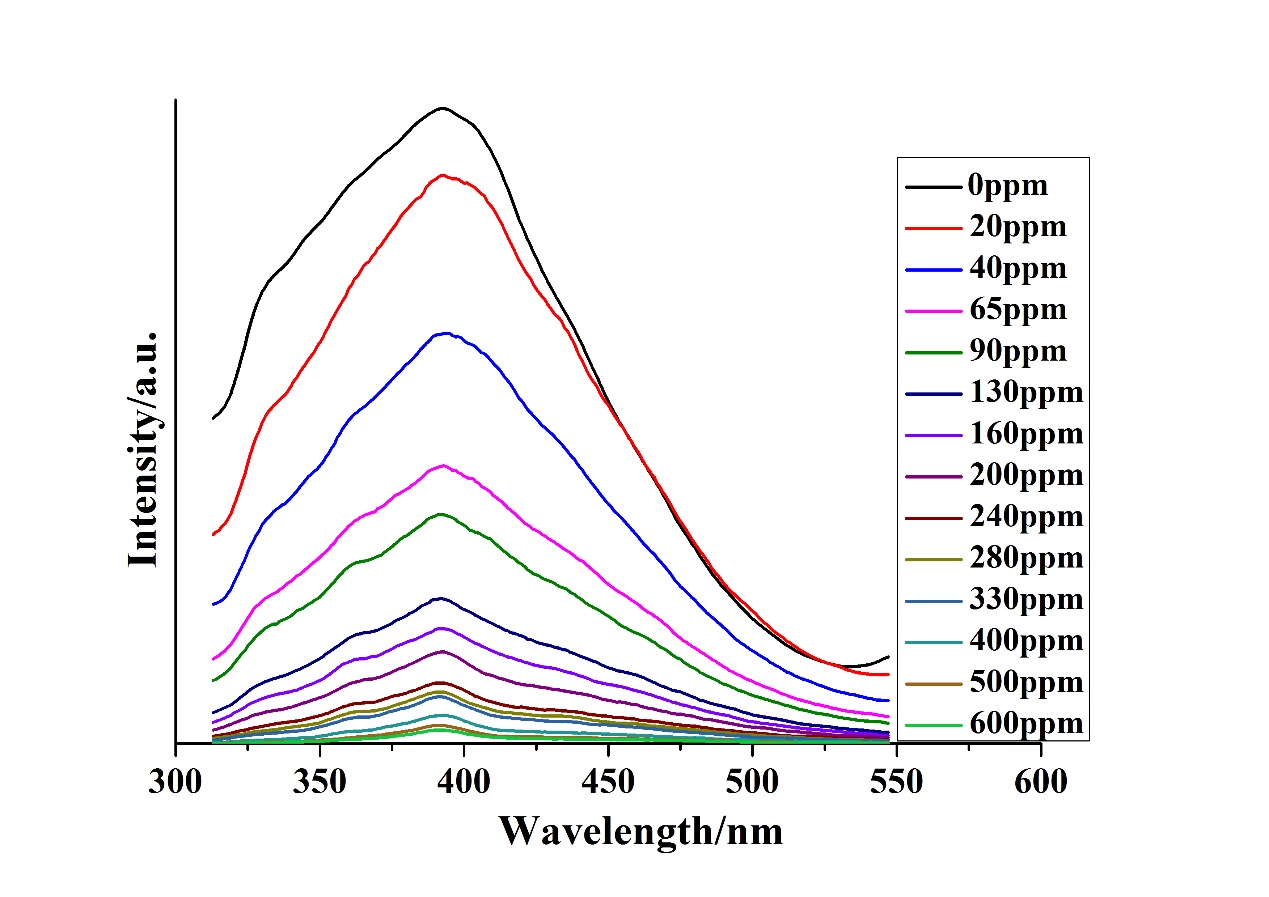


Fig. S10 Luminescent quenching of **1** dispersed in ethanol by the gradual addition of 1 mM solution of 2,4-DNT in DMF.


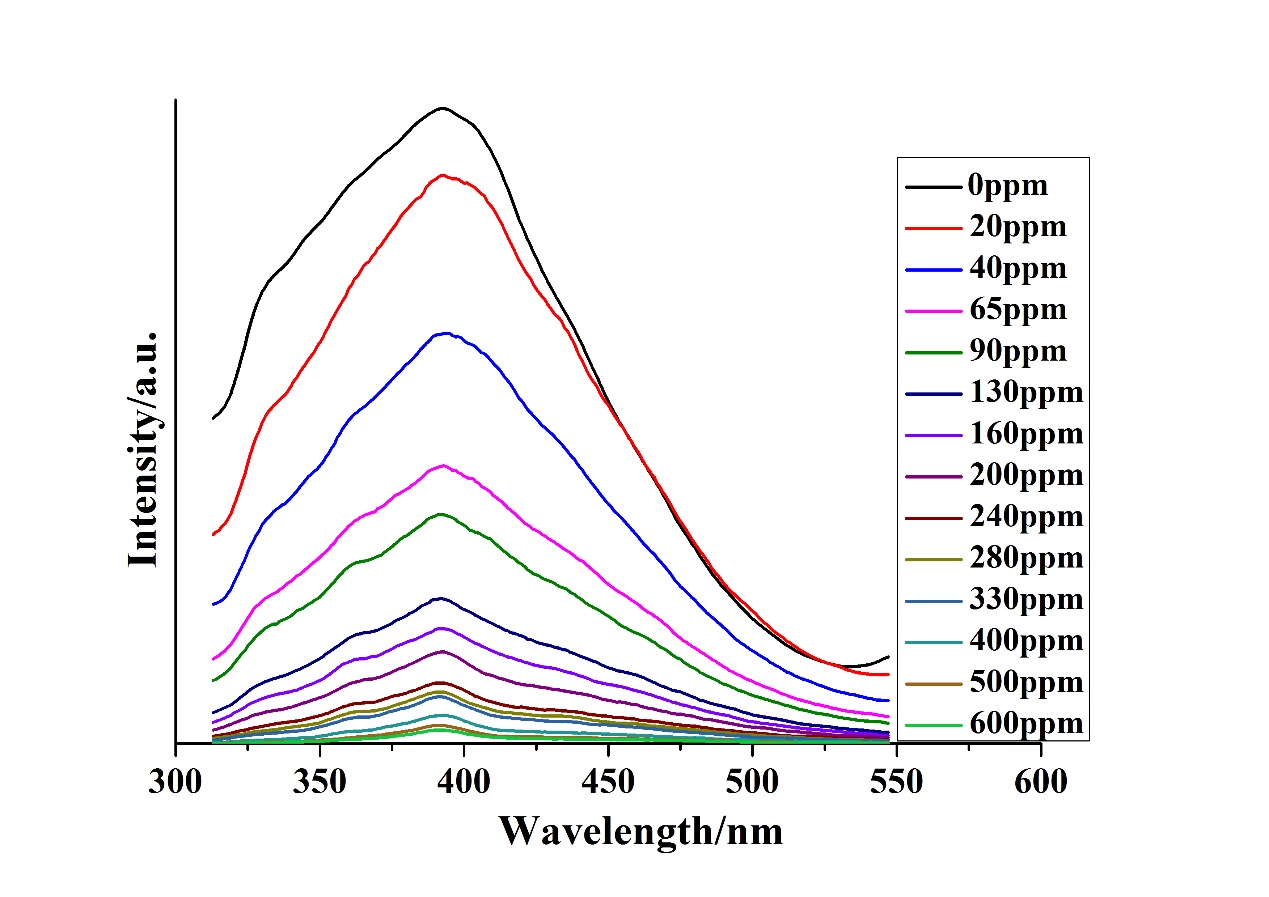


Fig. S11 Stern–Volmer plot for the fluorescence quenching of **1** upon the addition of 2,4-DNT.


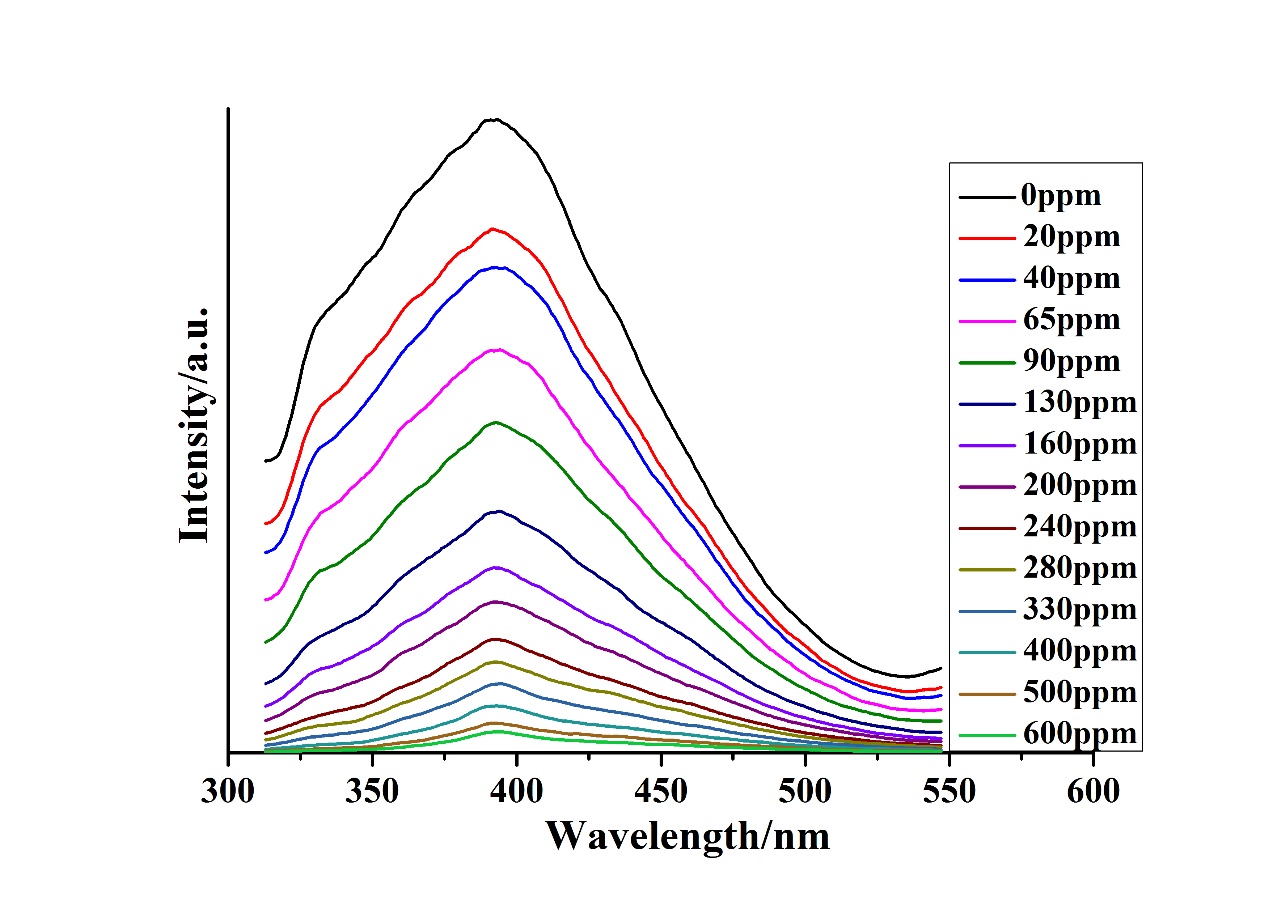


Fig. S12 Luminescent quenching of **1** dispersed in ethanol by the gradual addition of 1 mM solution of 2,6-DNT in DMF.


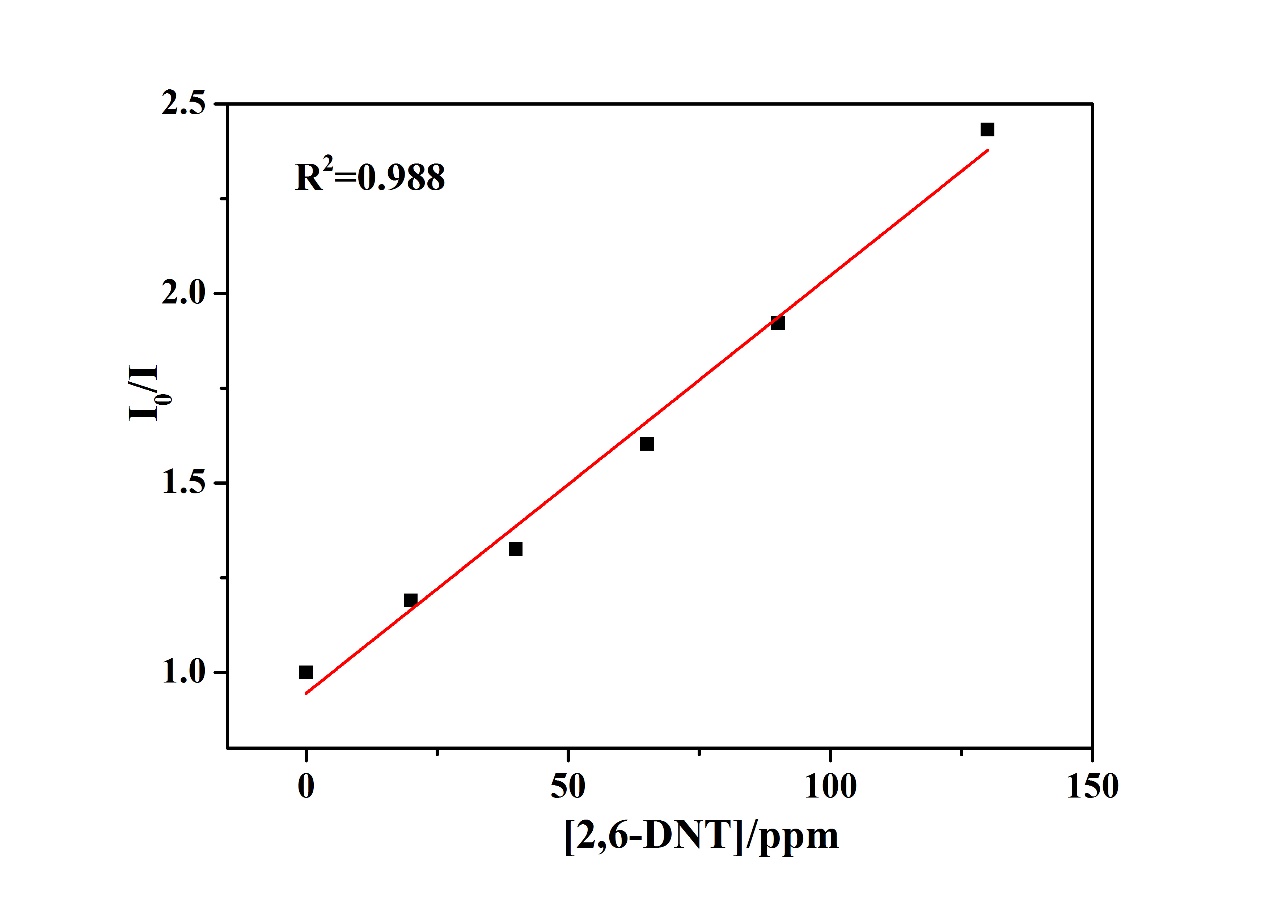


Fig. S13 Stern–Volmer plot for the fluorescence quenching of **1** upon the addition of 2,6-DNT.


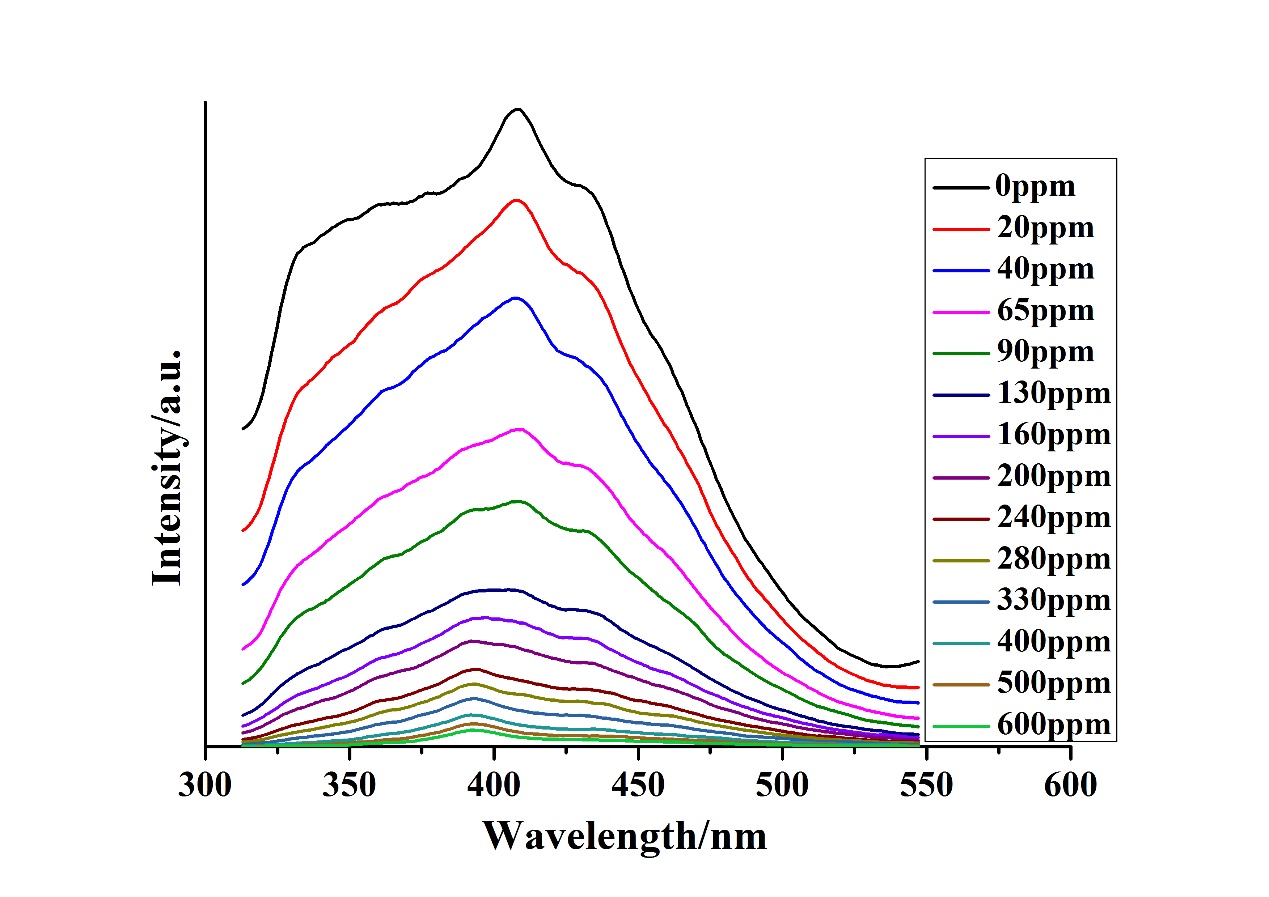


Fig. S14 Luminescent quenching of **1** dispersed in ethanol by the gradual addition of 1 mM solution of 2-NT in DMF.


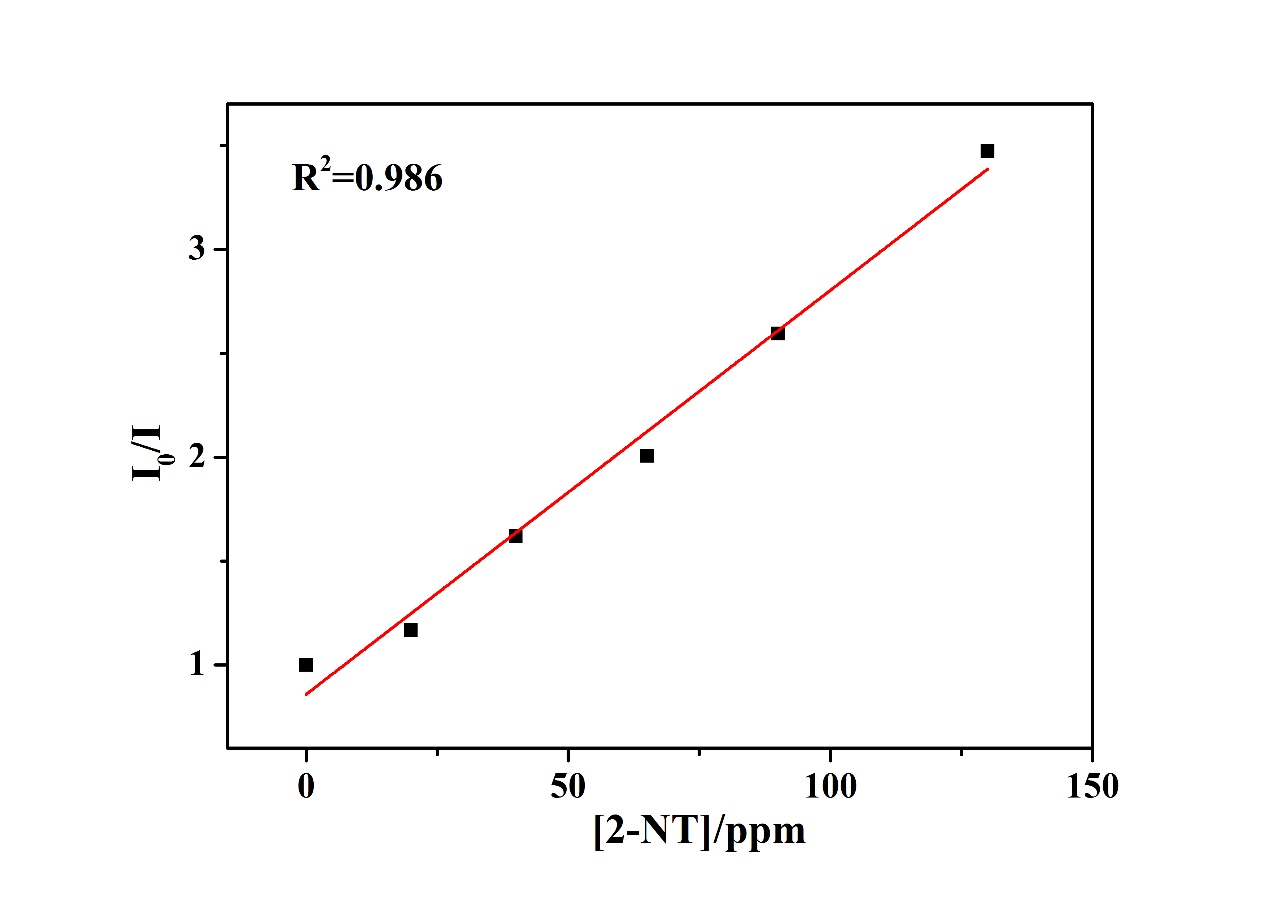


Fig. S15 Stern–Volmer plot for the fluorescence quenching of **1** upon the addition of 2-NT.


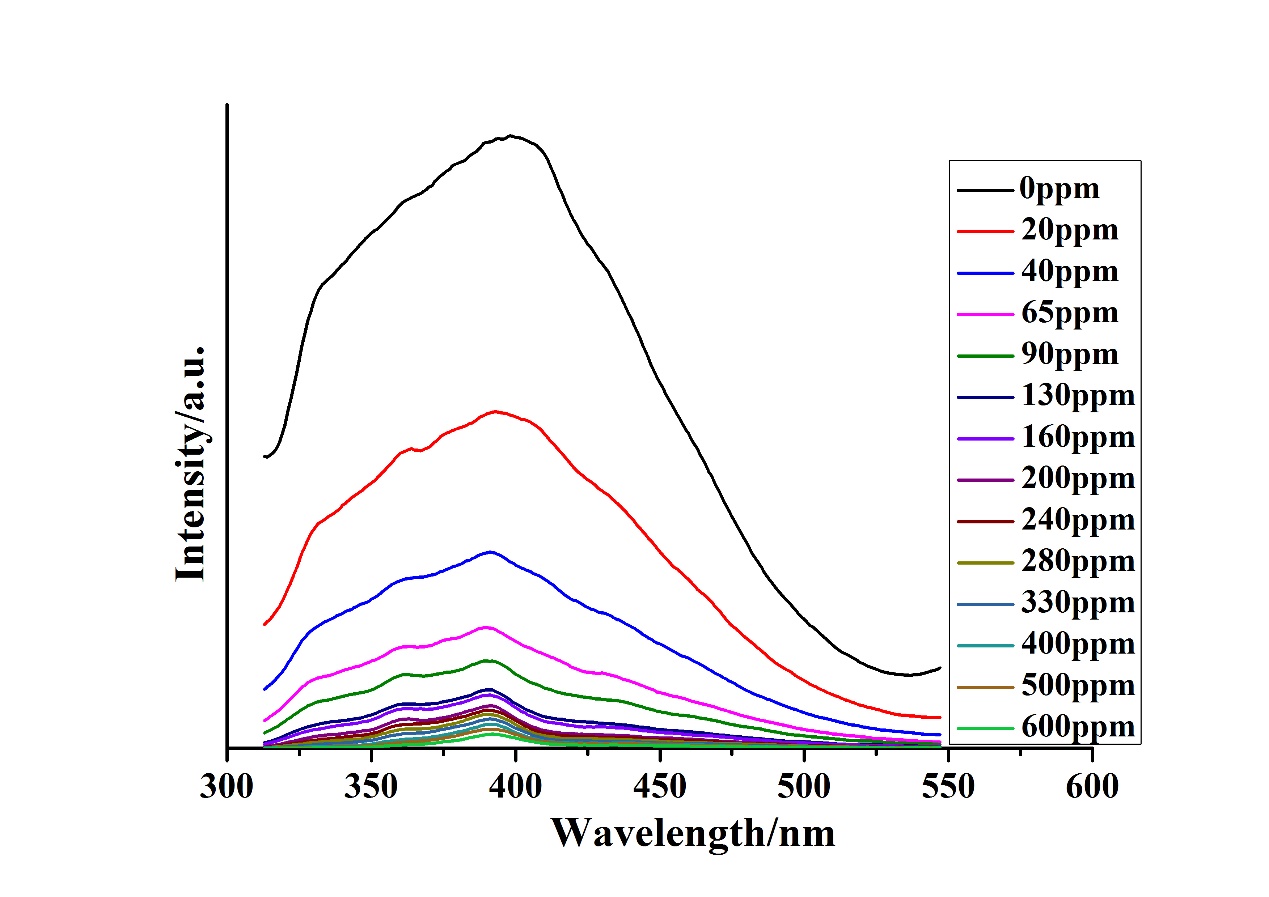


Fig. S16 Luminescent quenching of **1** dispersed in ethanol by the gradual addition of 1 mM solution of 4-NT in DMF.


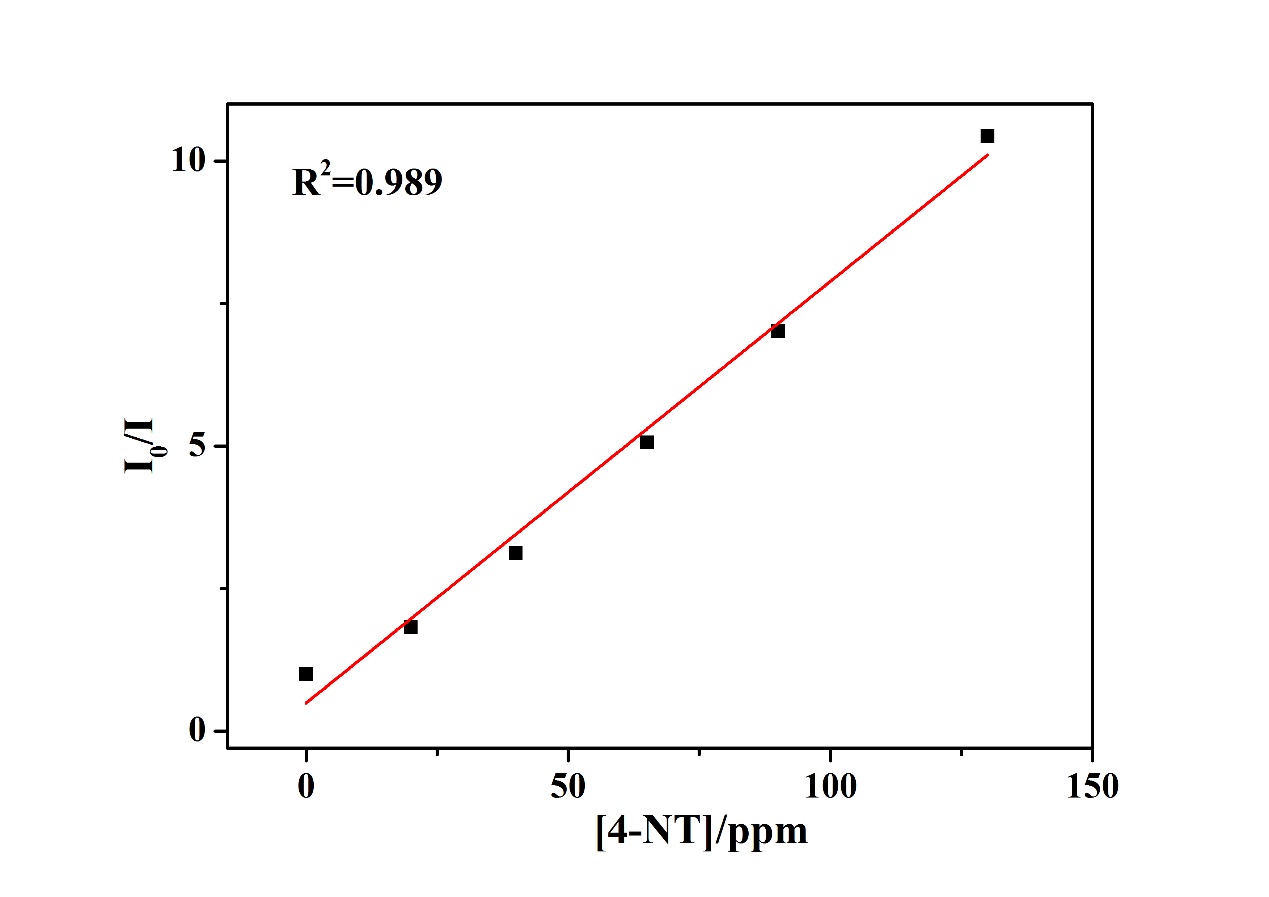


Fig. S17 Stern–Volmer plot for the fluorescence quenching of **1** upon the addition of 4-NT.


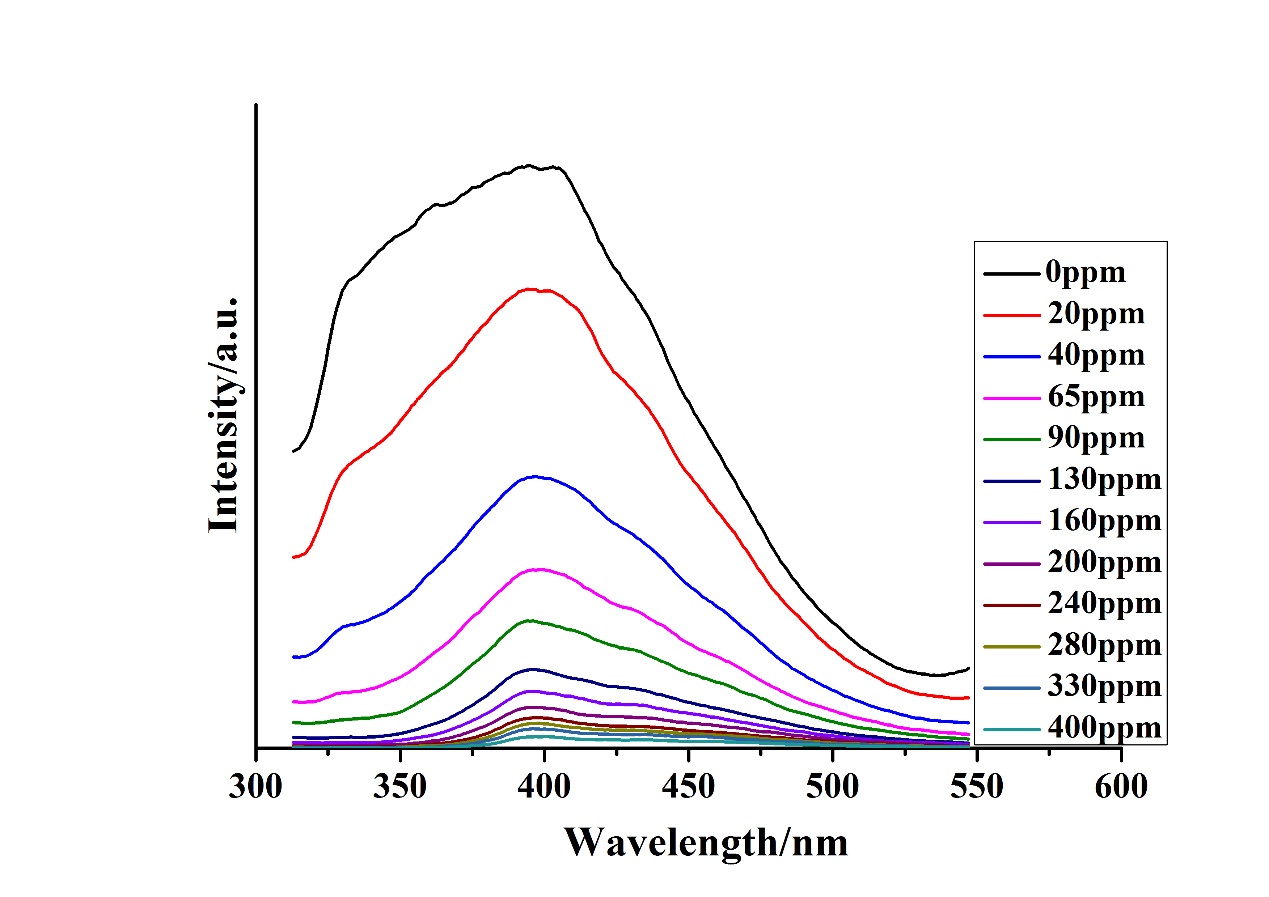


Fig. S17 Luminescent quenching of **1** dispersed in ethanol by the gradual addition of 1 mM solution of MNP in DMF.


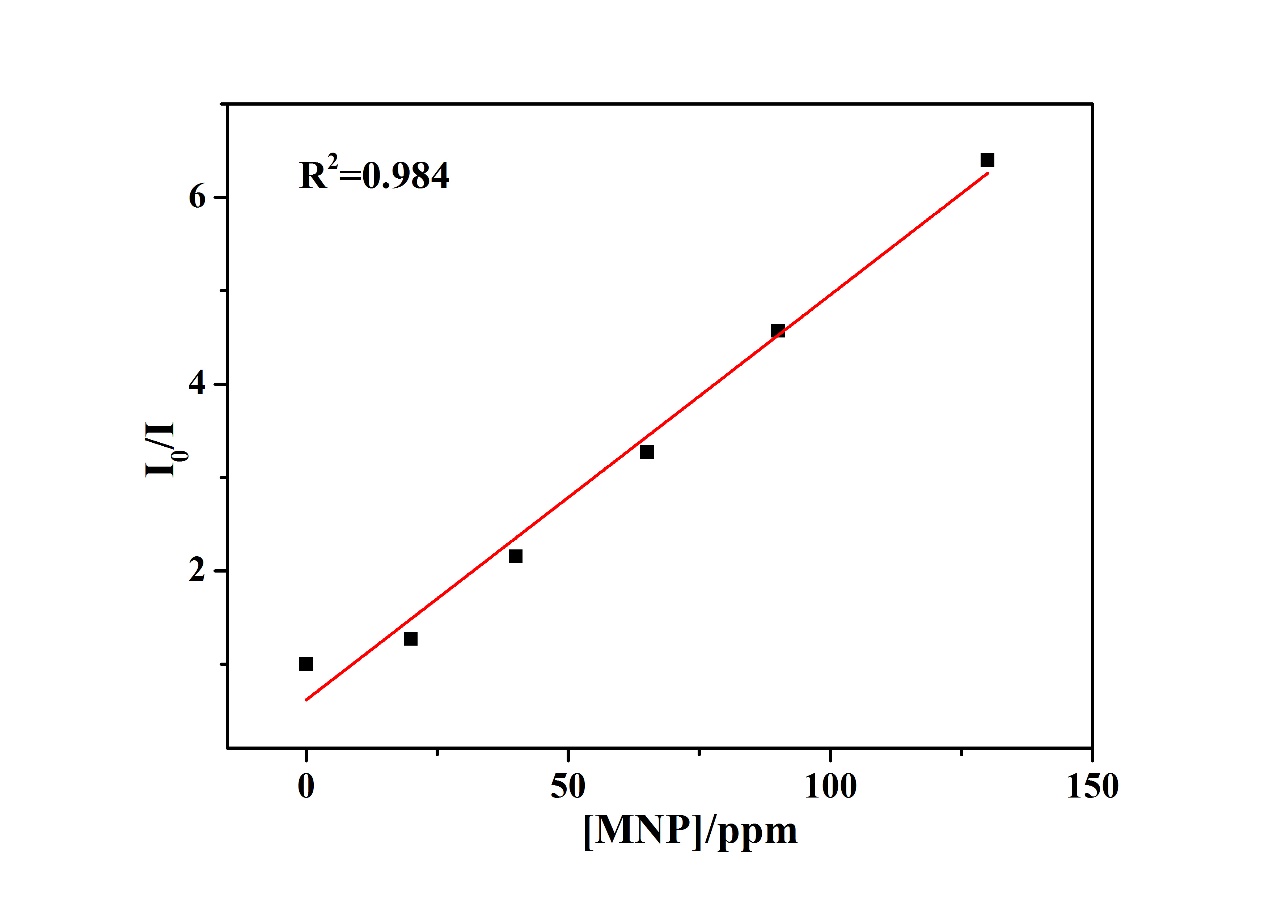


Fig. S18 Stern–Volmer plot for the fluorescence quenching of **1** upon the addition of MNP.


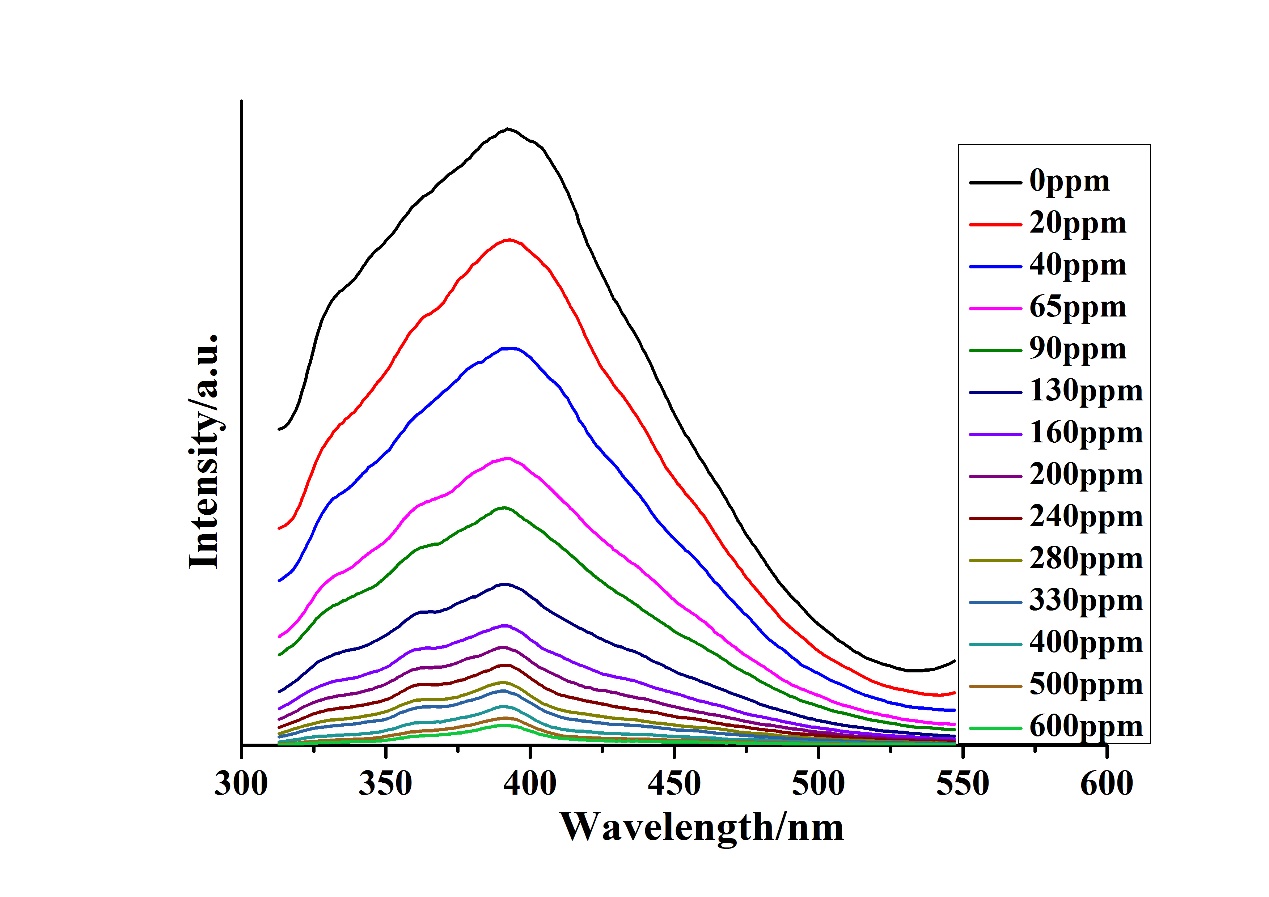


Fig. S19 Luminescent quenching of **1** dispersed in ethanol by the gradual addition of 1 mM solution of NB in DMF.


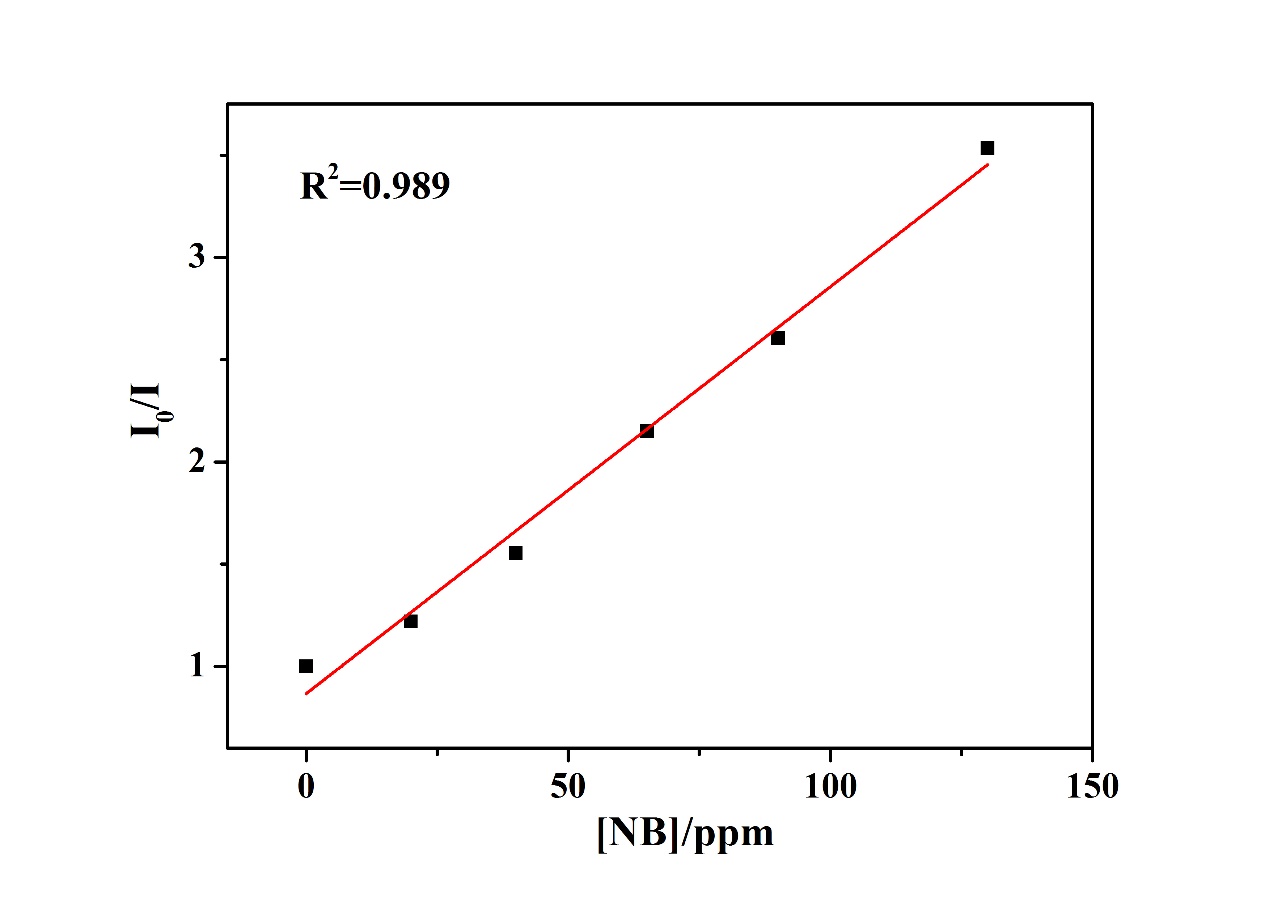


Fig. S20 Stern–Volmer plot for the fluorescence quenching of **1** upon the addition of NB.


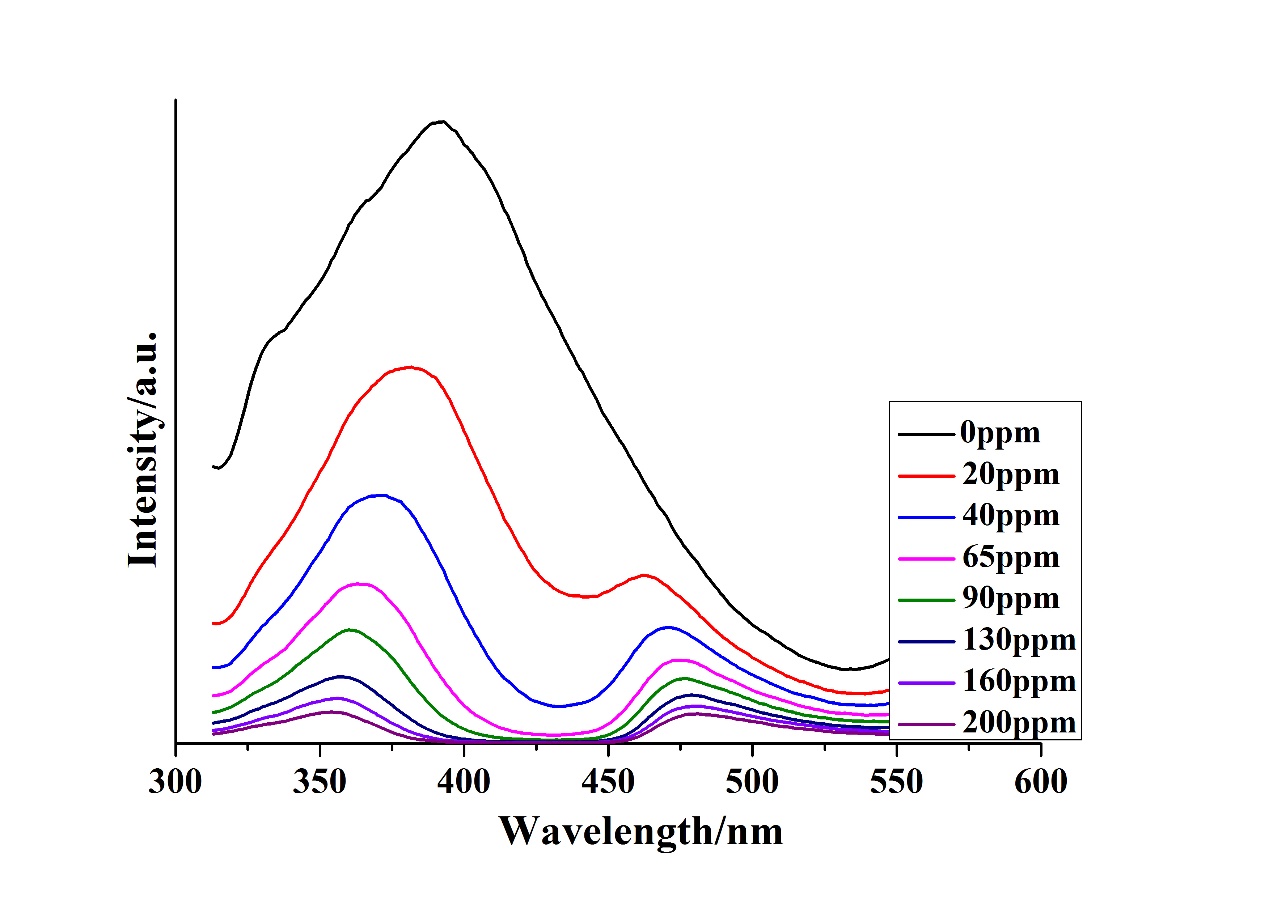


Fig. S21 Luminescent quenching of **1** dispersed in ethanol by the gradual addition of 1 mM solution of PNP in DMF.


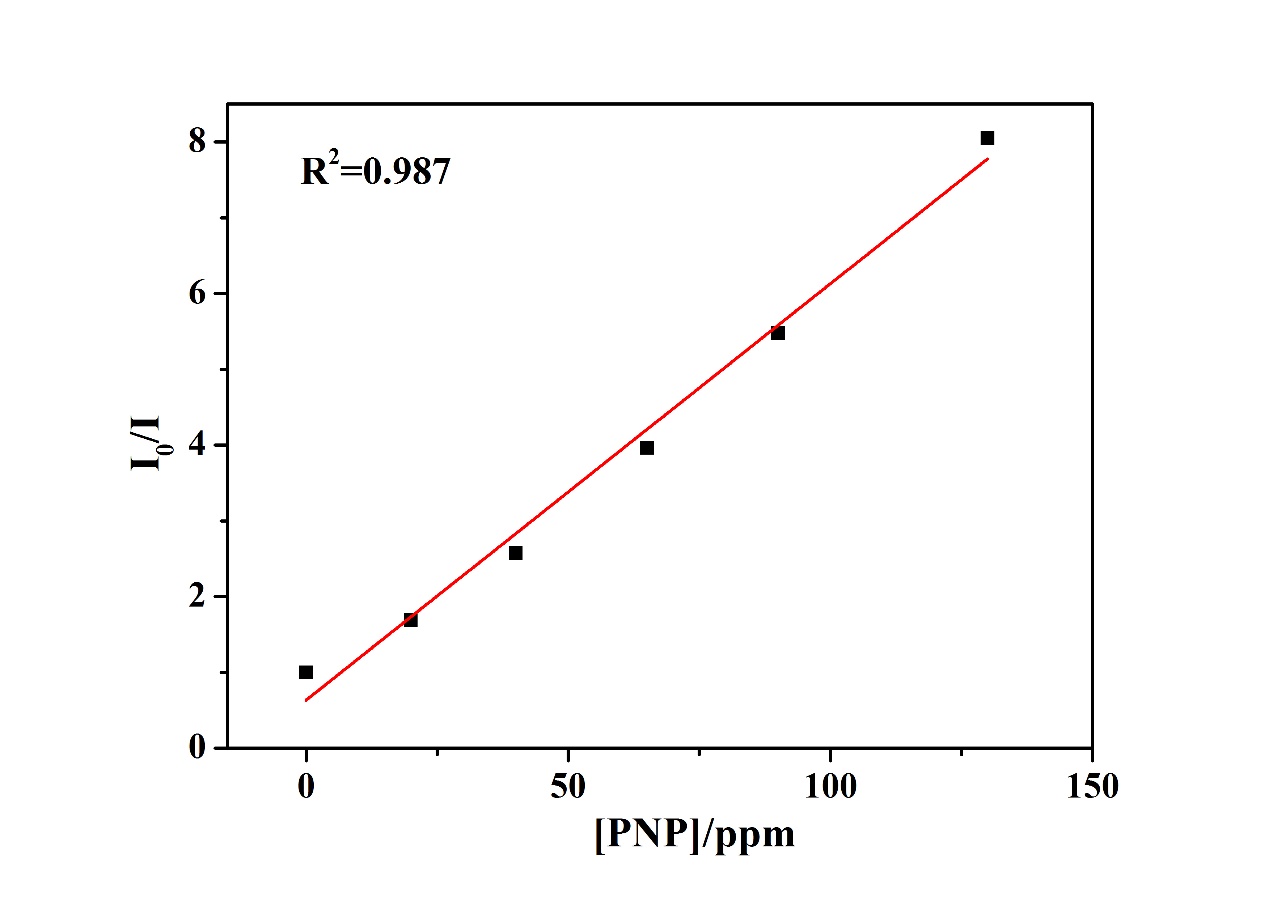


Fig. S22 Stern–Volmer plot for the fluorescence quenching of **1** upon the addition of PNP.


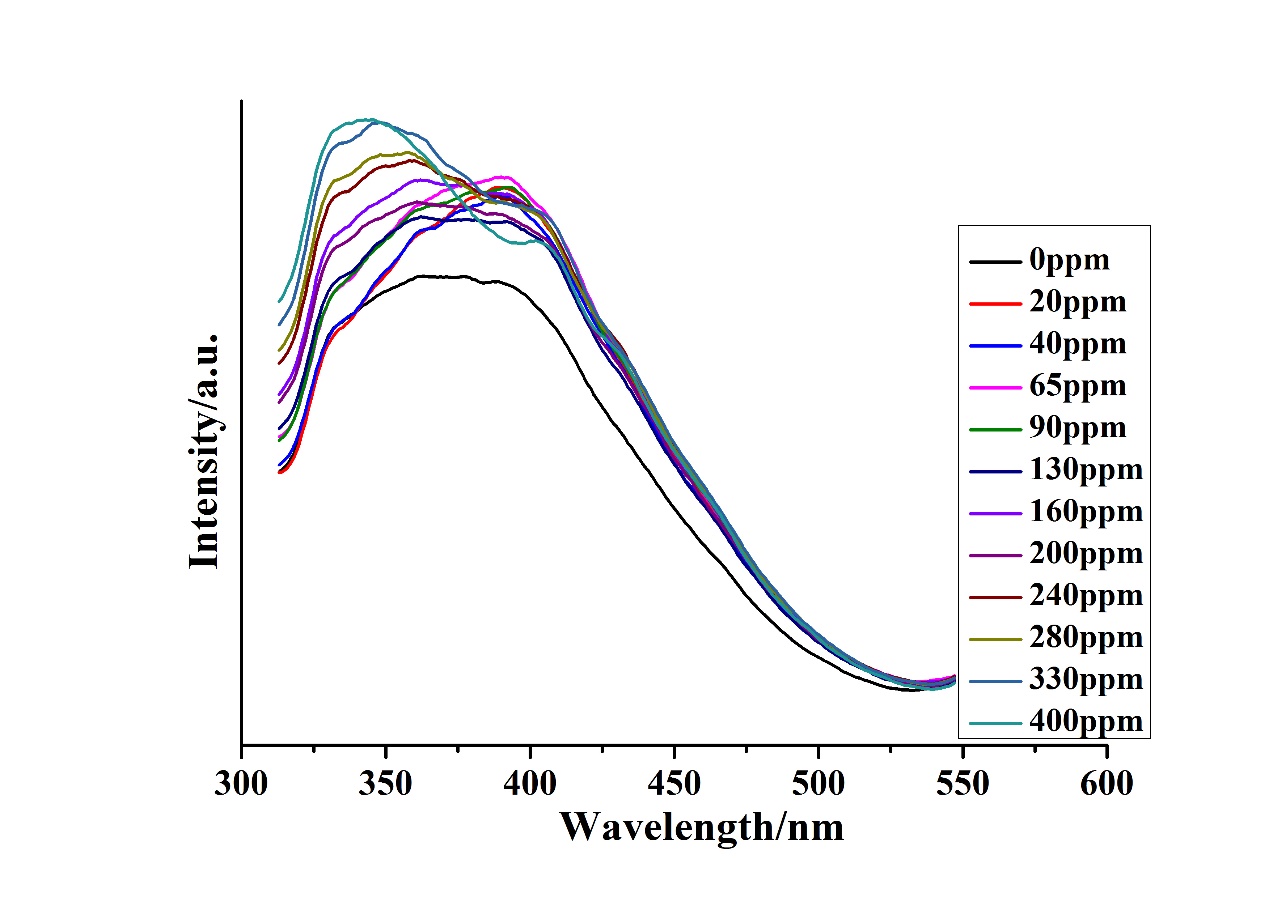


Fig. S23 Luminescent quenching of **1** dispersed in ethanol by the gradual addition of 1 mM solution of 1,2,4-TMB in DMF.


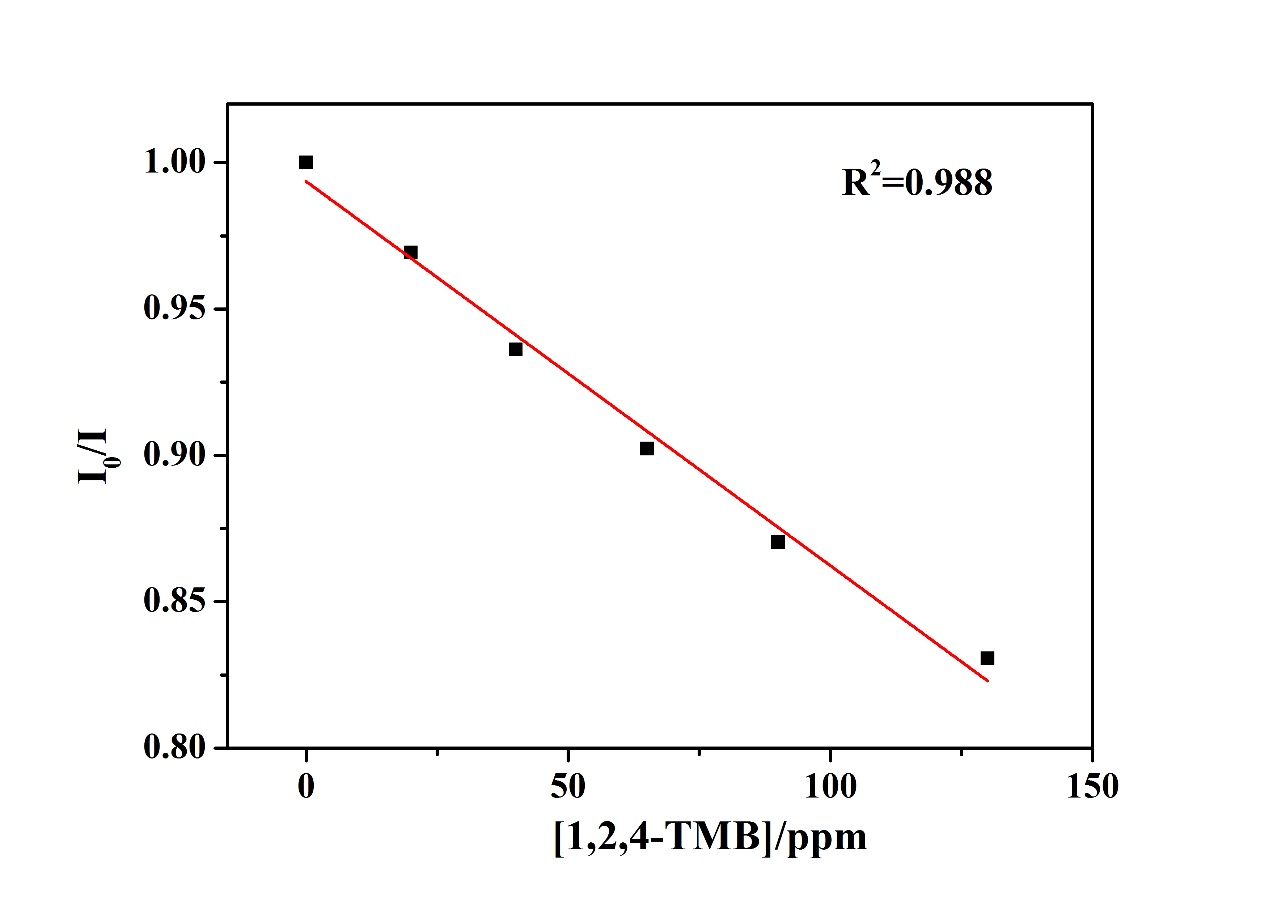


Fig. S24 Stern–Volmer plot for the fluorescence quenching of **1** upon the addition of 1,2,4-TMB.


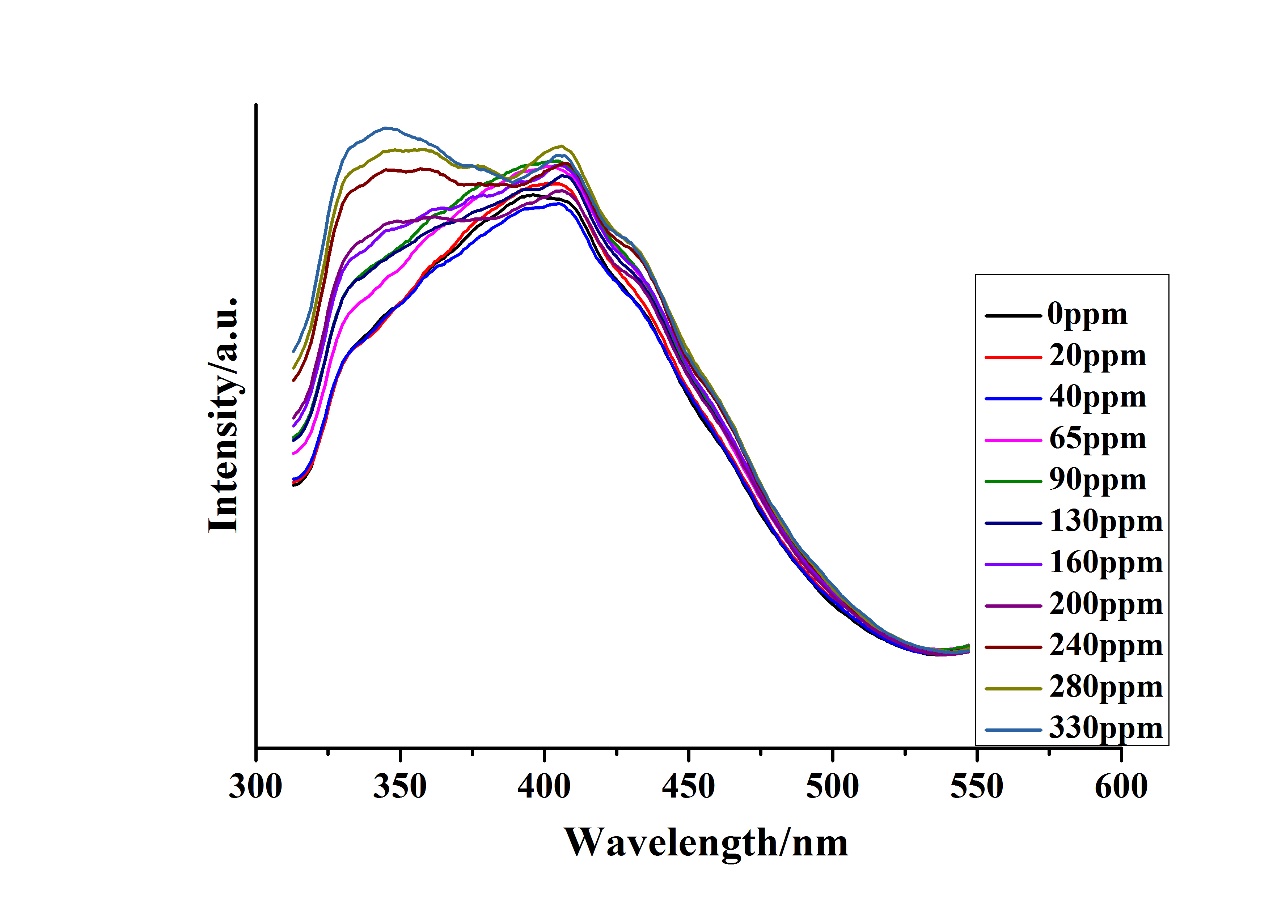


Fig. S25 Luminescent quenching of **1** dispersed in ethanol by the gradual addition of 1 mM solution of 1,3,5-TMB in DMF.


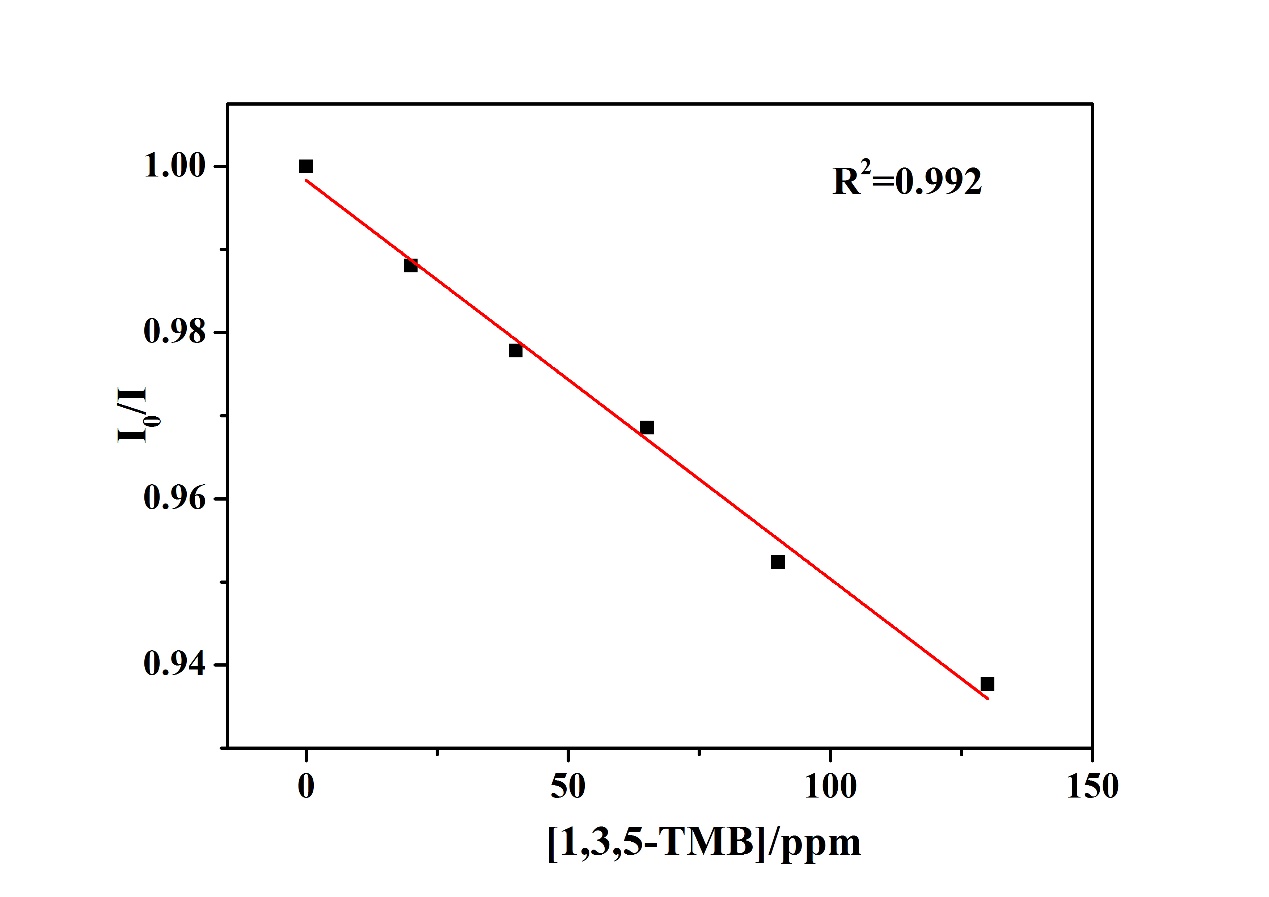


Fig. S26 Stern–Volmer plot for the fluorescence quenching of **1** upon the addition of 1,3,5-TMB.

| 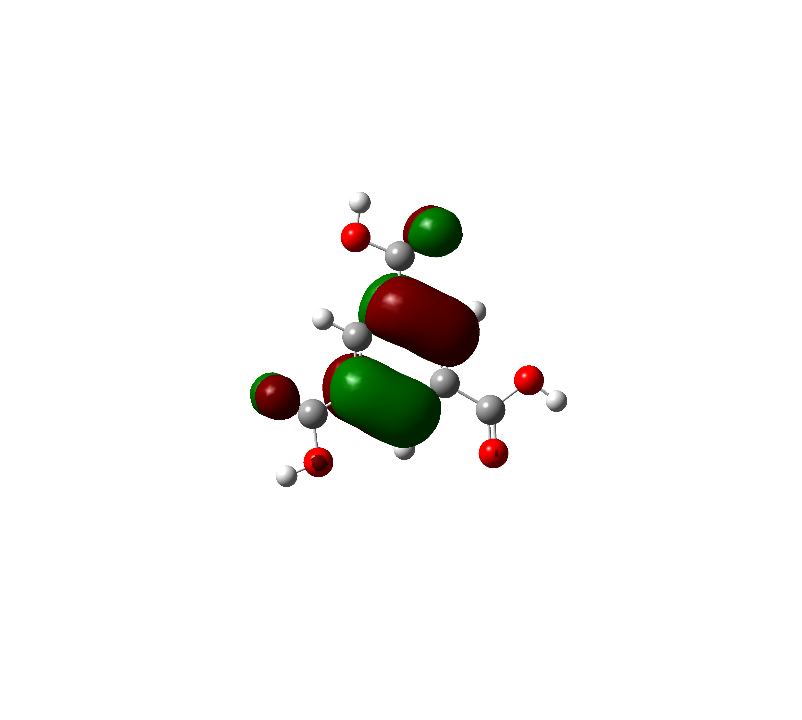  HOMO H**_3_**L | 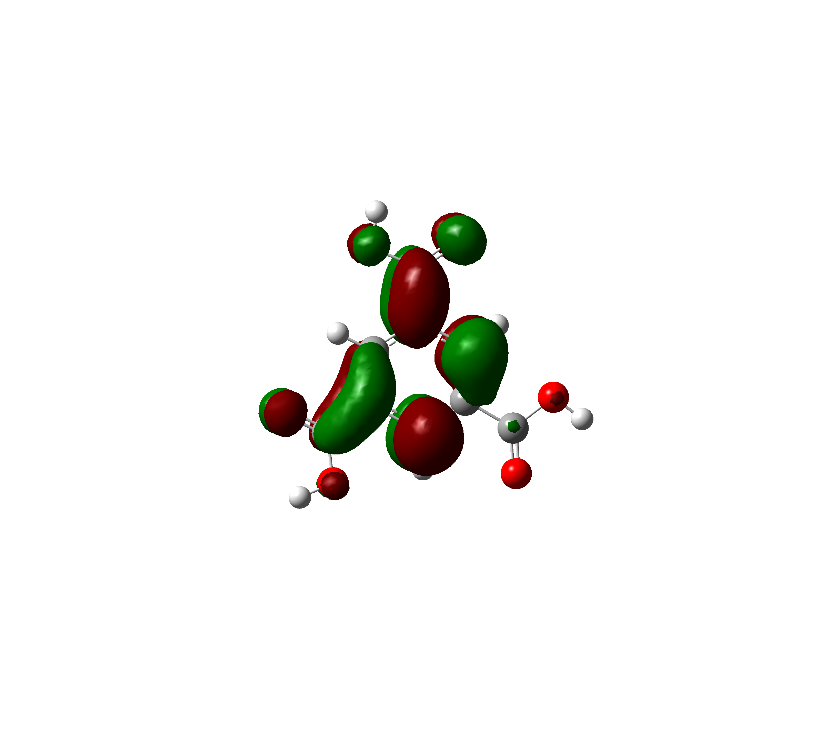  LUMO H_3_L |
| --- | --- |
| 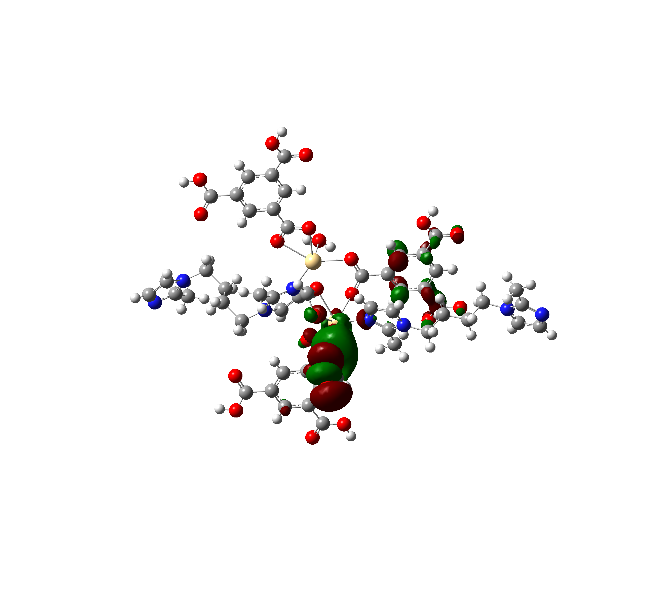  HOMO **1** | 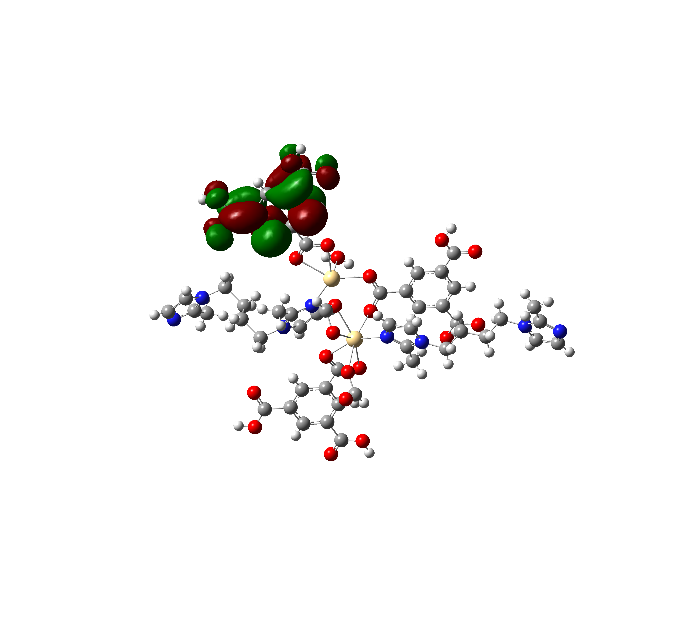  LUMO **1** |
| 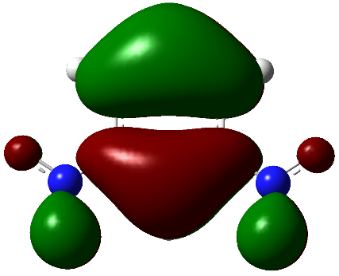  HOMO 1,3-DNB | 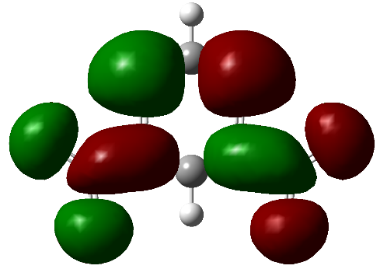  LUMO 1,3-DNB |
| 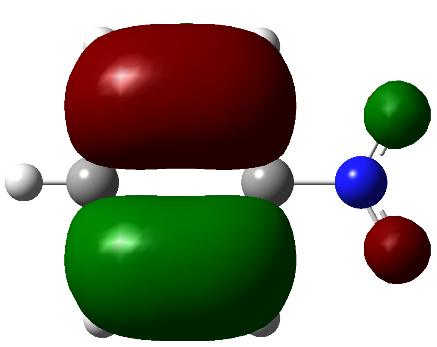  HOMO NB | 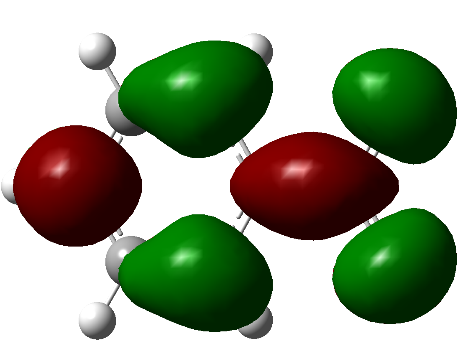  LUMO NB |
| 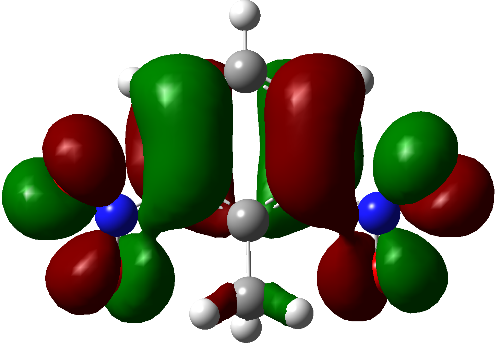  HOMO 2,6-DNT | 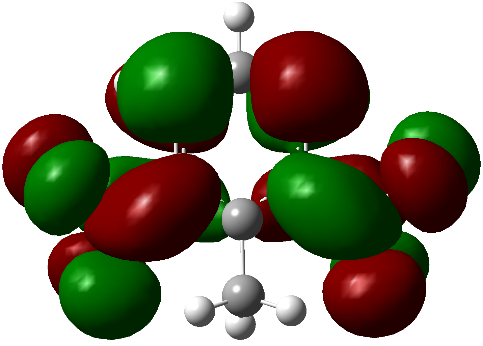  LUMO 2,6-DNT |
| 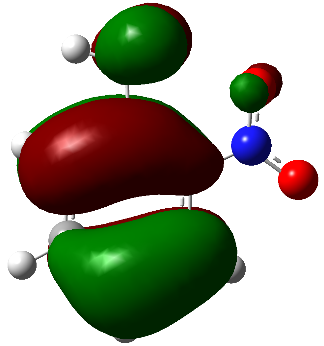  HOMO 2-NT | 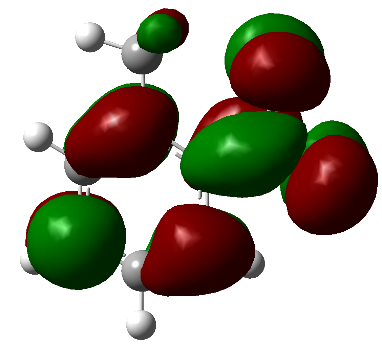  LUMO 2-NT |
| 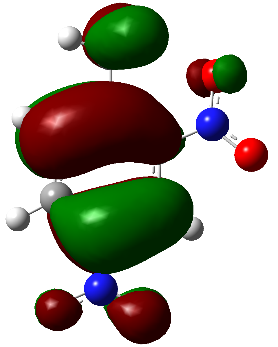  HOMO 2,4-DNT | 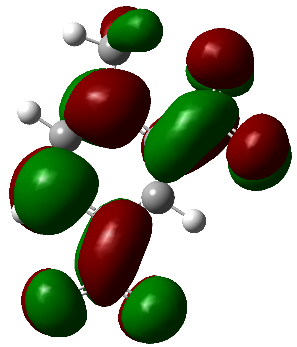  LUMO 2,4-DNT |
| 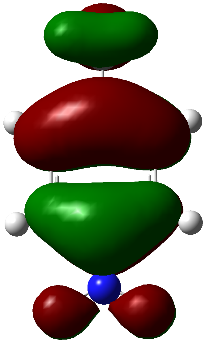  HOMO 4-NT | 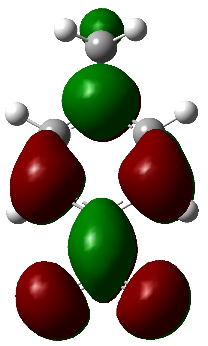  LUMO 4-NT |
| 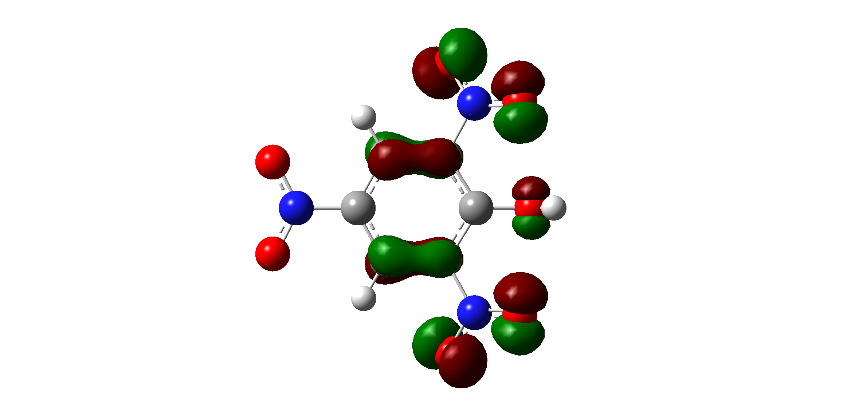  HOMO TNP | 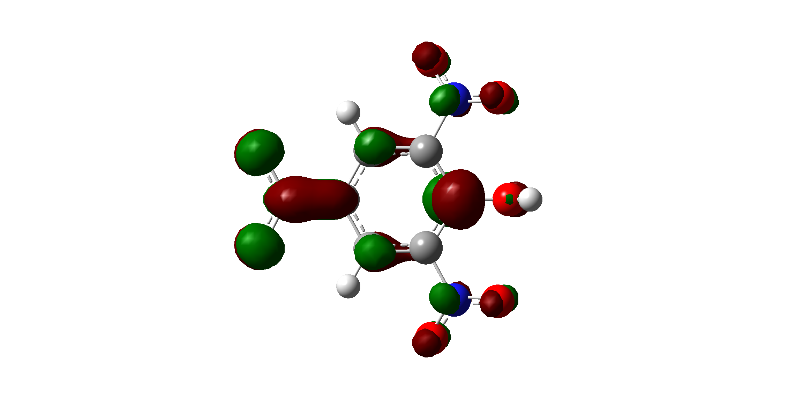  LUMO TNP |
| 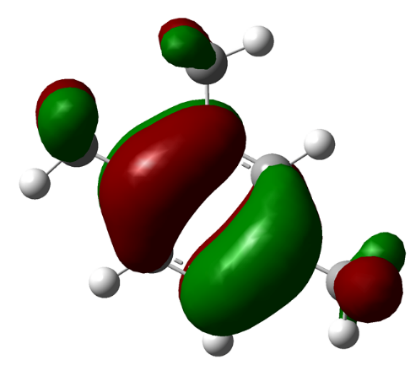  HOMO 1,2,4-TMB | 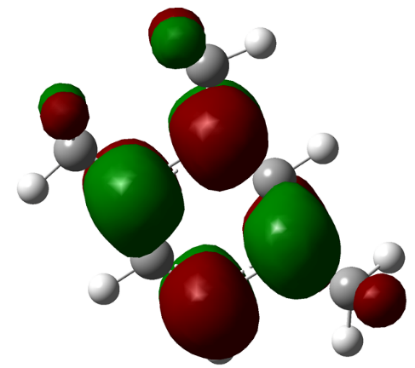  LUMO 1,2,4-TMB |
| 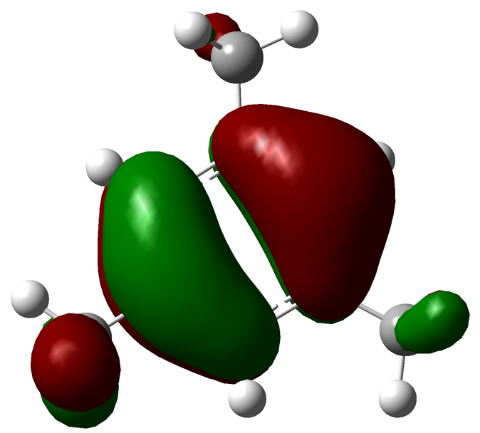  HOMO 1,3,5-TMB | 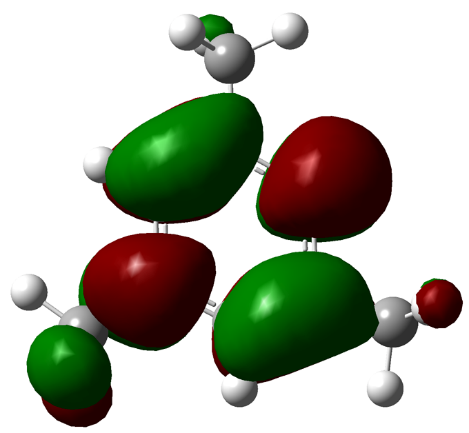  LUMO 1,3,5-TMB |
| 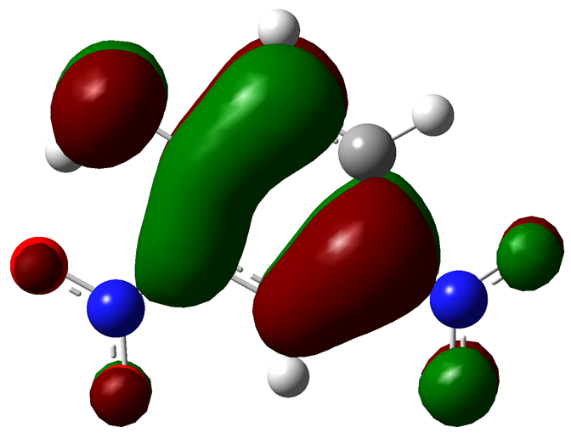  HOMO 2,4-DNP | 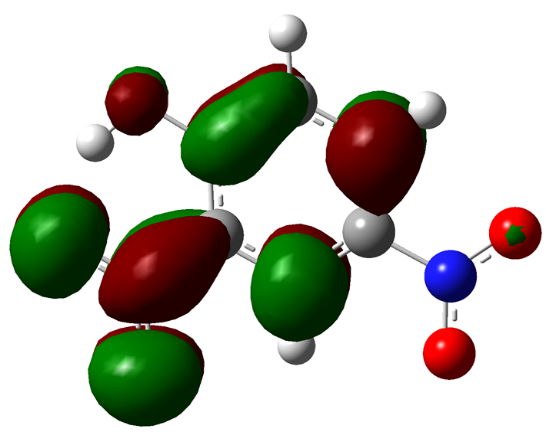  LUMO 2,4-DNP |
| 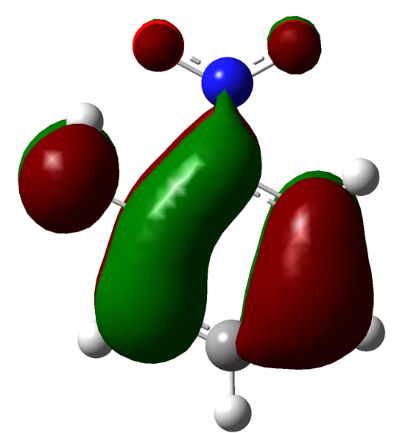  HOMO o-nitro phenol | 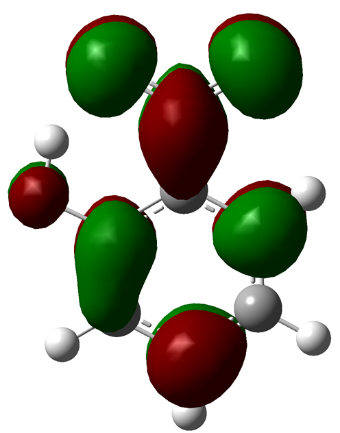  LUMO o-nitro phenol |
| 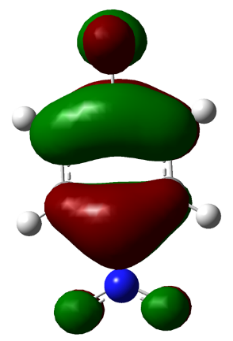  HOMO p-nitrophenol | 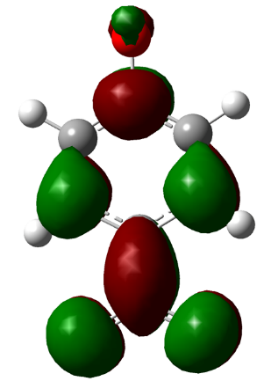  LUMO p-nitrophenol |

Fig. S27 HOMO–LUMO energies of the NACs along with CP **1** and ligands.


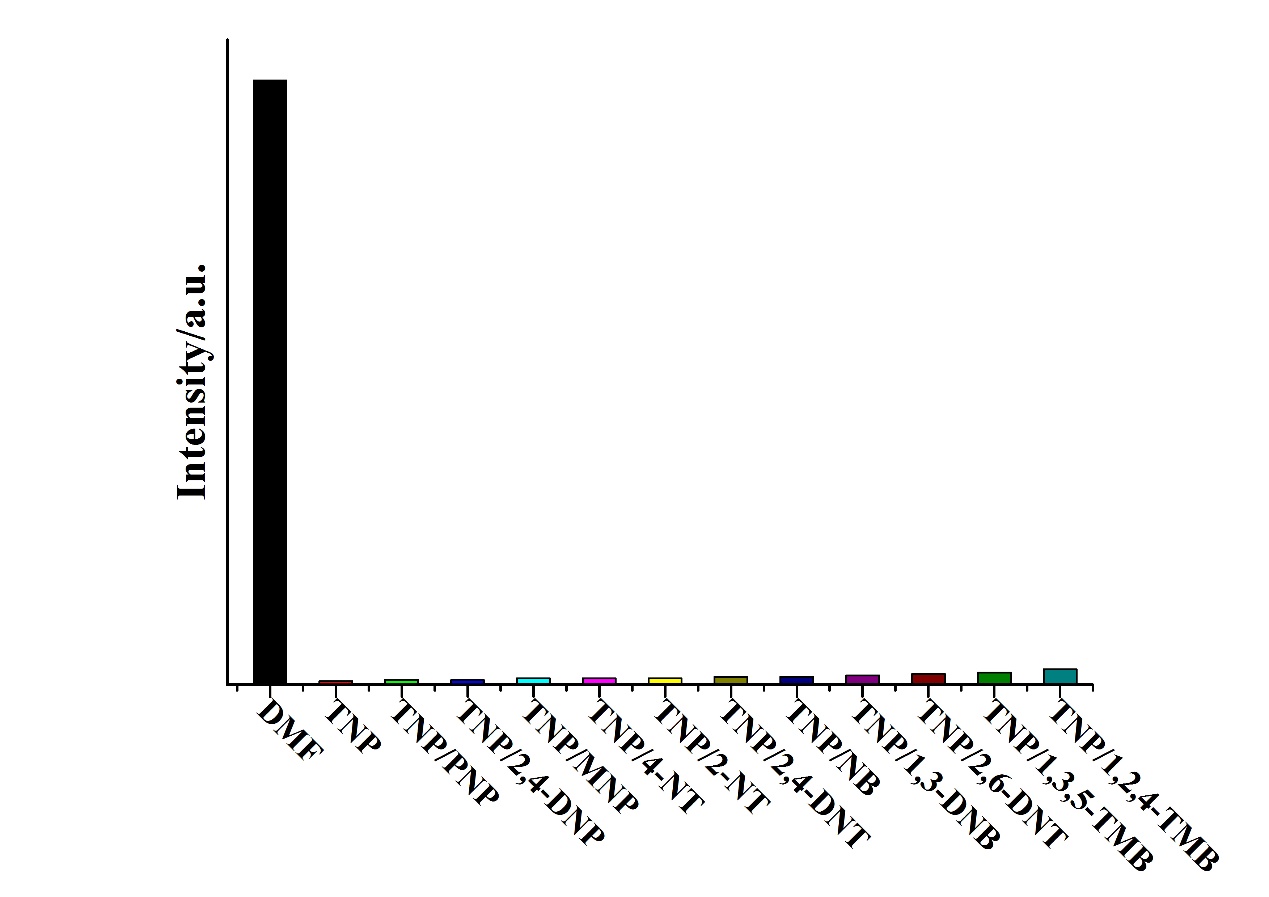


Fig. S28 The Emission spectra of the suspension of **1** upon the addition of NACs followed by TNP.

| 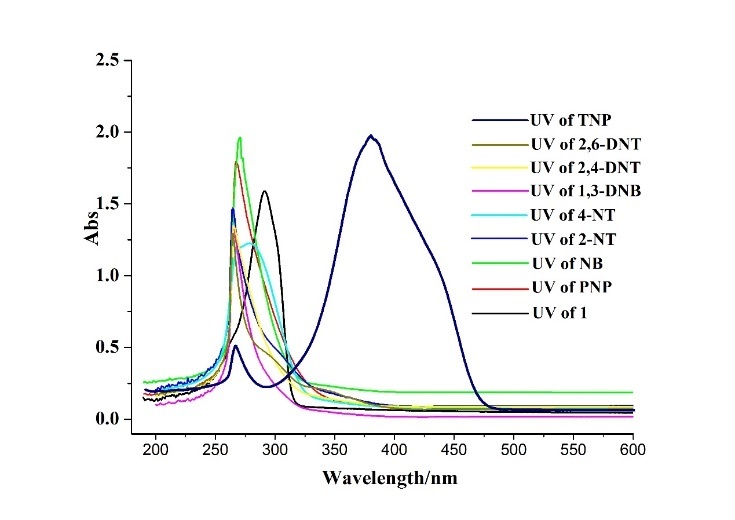 | 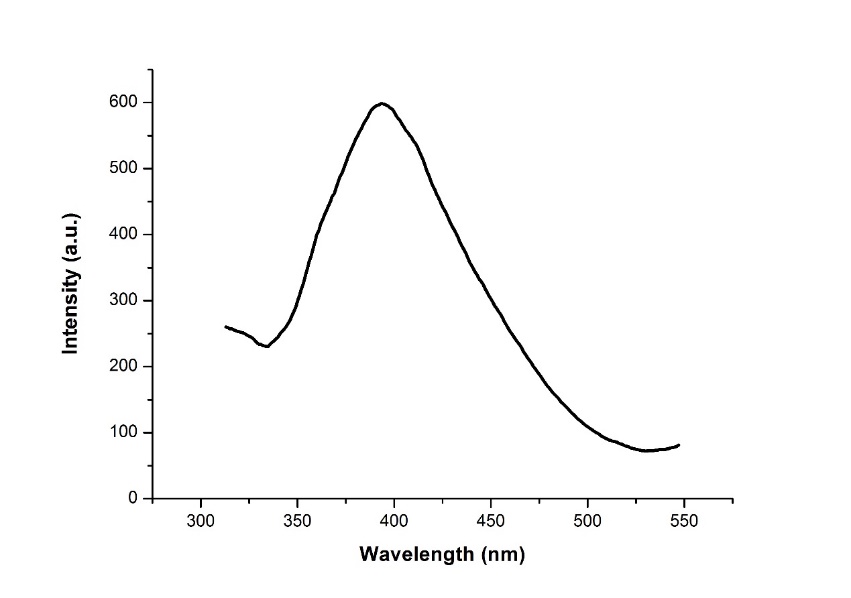 |
| --- | --- |

Fig. S29 The absorption spectra of NACs investigated (left) and the emission spectrum of **1** (right).


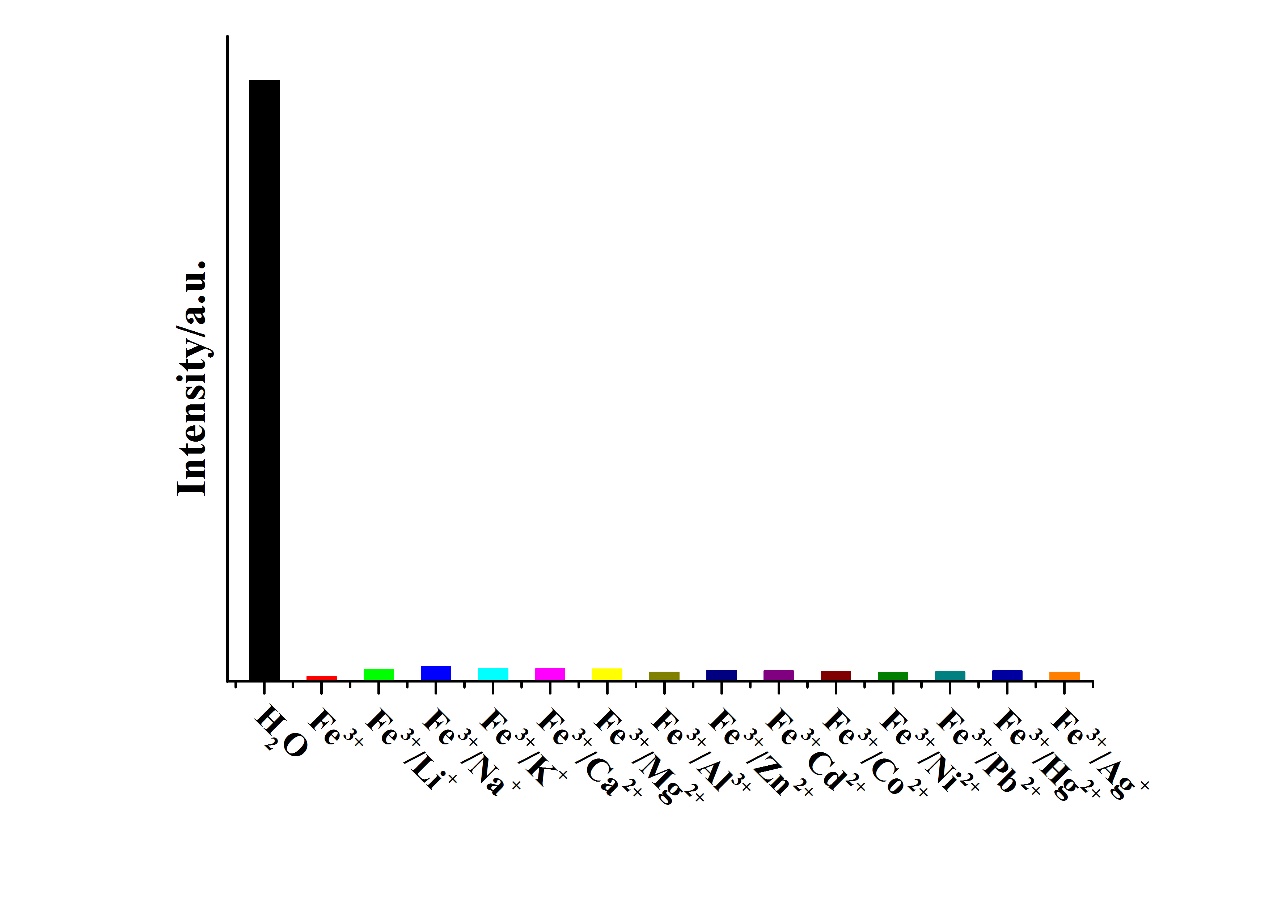


Fig. S30 Luminescence intensity of **1** dispersed in H_2_O with addition of different mixed ions (10^-2^ M) and Fe^3+^-incorporated systems (10^-2^ M).


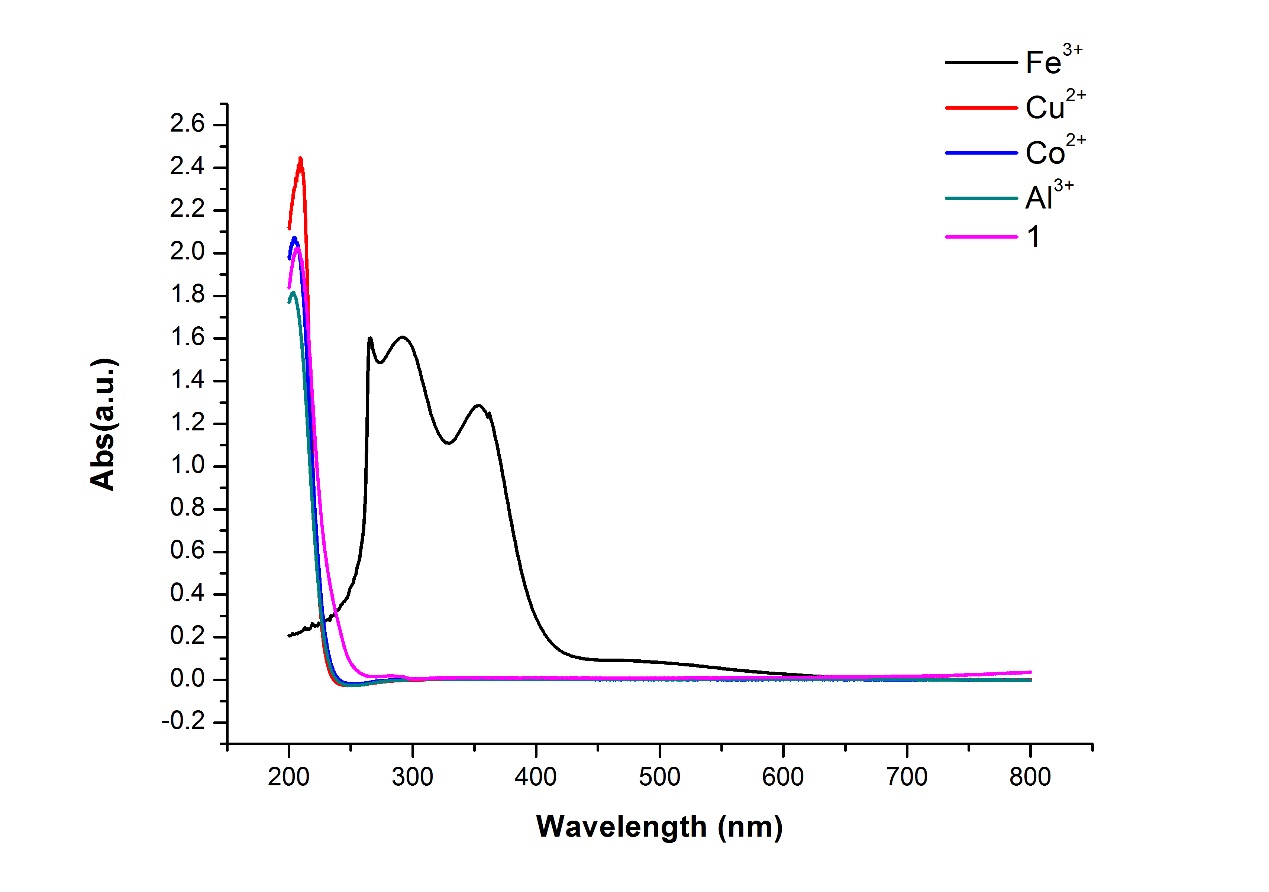


Fig. S31 UV-Vis adsorption spectrum of M(NO_3_)_X_ aqueous solution and the excitation spectrum of **1**.


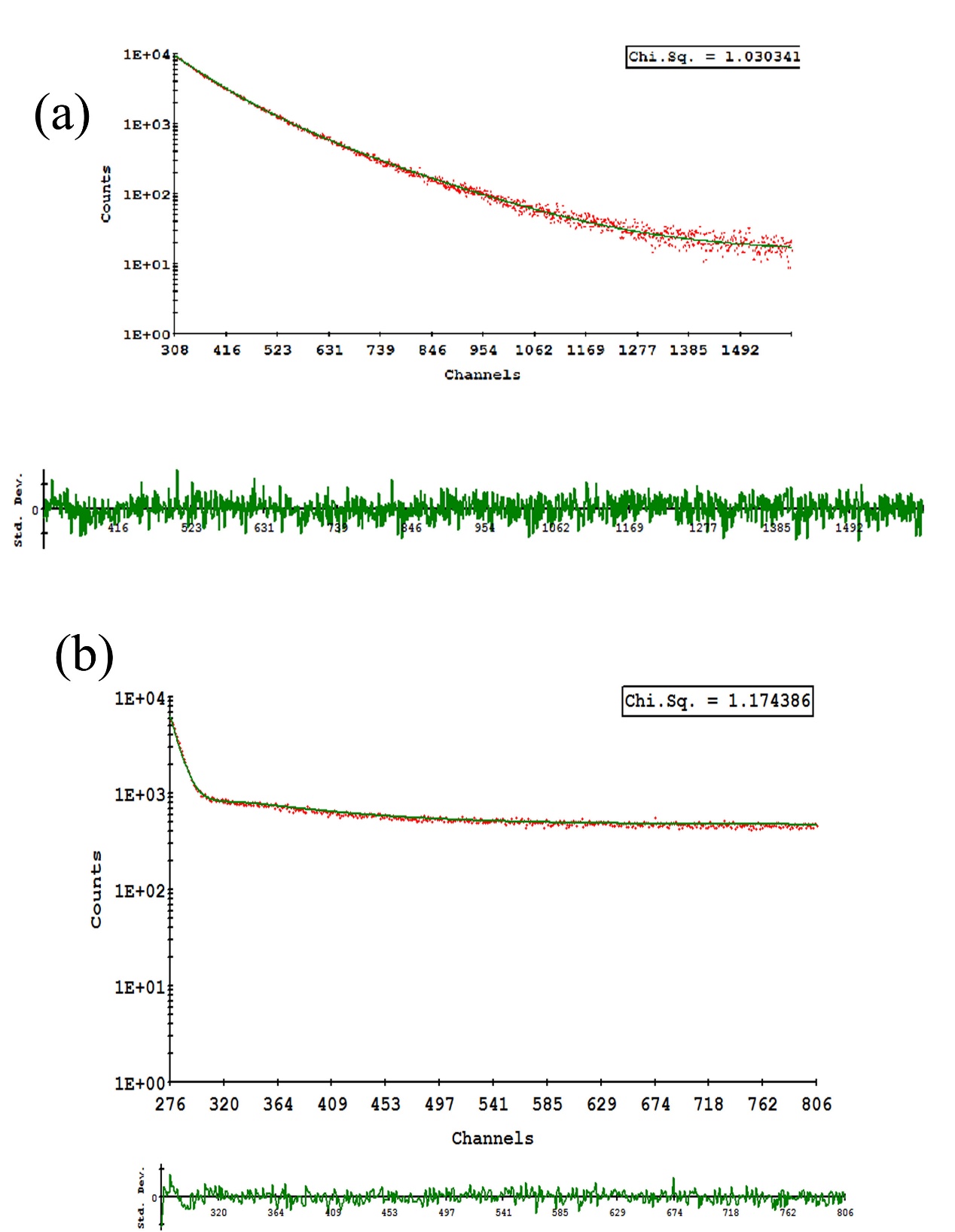


Fig. S32 Luminescence decay curves for **1** (a) and Fe^3+^@**1** (b).

**
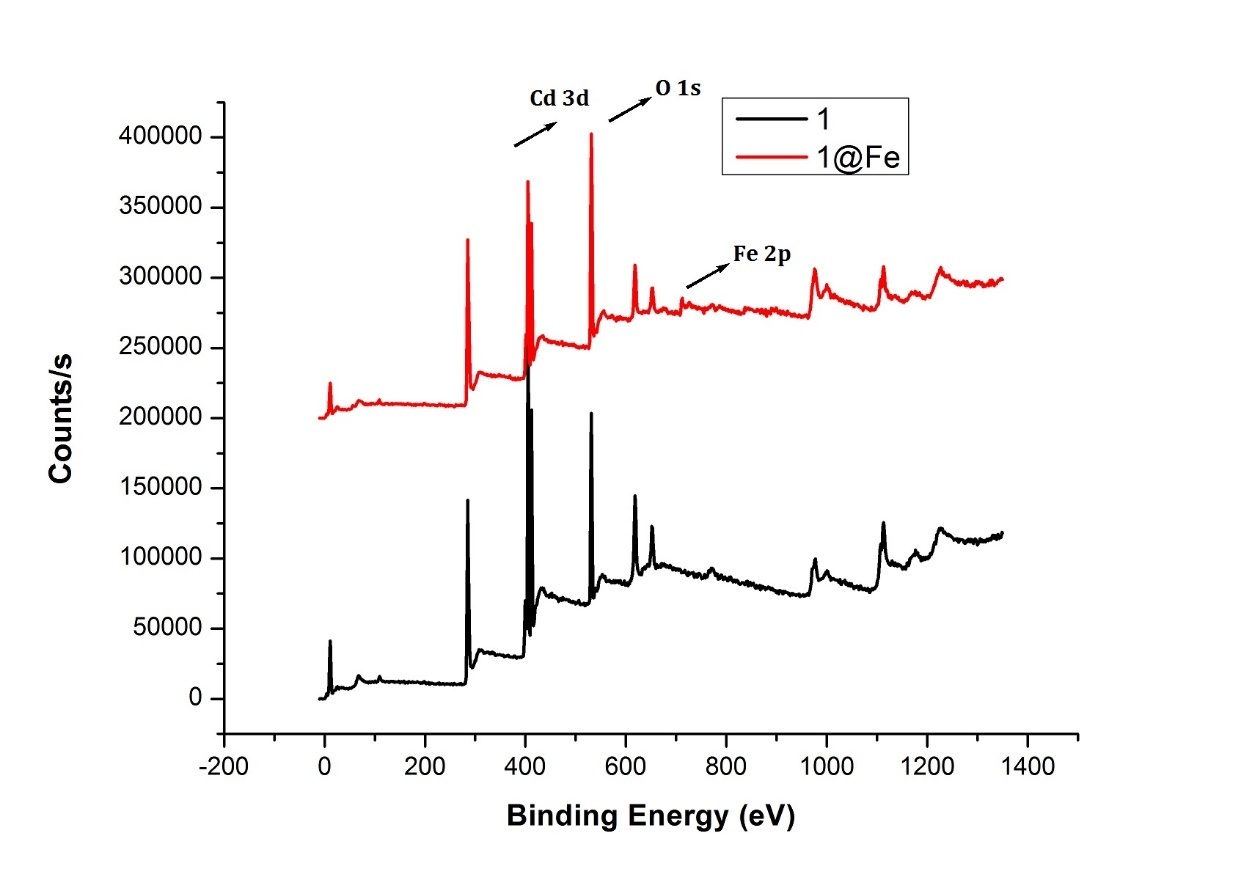
**

Fig. S33 X-ray photo-electron spectrum of Fe^3+^@**1**.

**
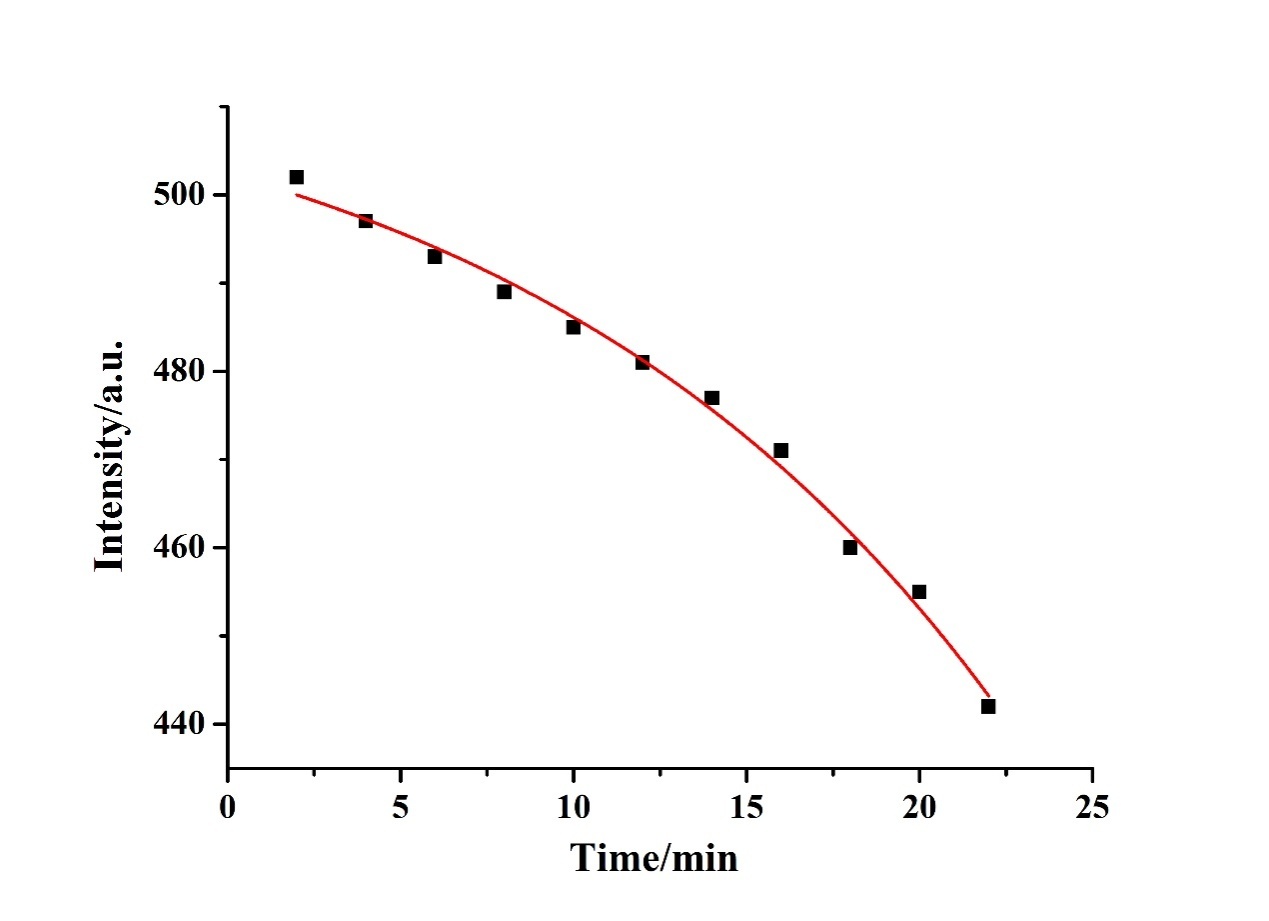
**

Fig. S34 The time-dependent intensity of **1**.

**Table S1. Crystallographic data and structure refinement details for 1**

| Parameter | **1** |
| --- | --- |
| Formula weight | 731.27 |
| Crystal system | Triclinic |
| Space group | P-1 |
| Crystal Cdlor | Colorless |
| *a*, Å | 9.7464(6) |
| *b*, Å | 10.3186(6) |
| *c*, Å | 13.9696(8) |
| *α*, ° | 97.0311(6) |
| *β*, ° | 109.4937(6) |
| *γ*, ° | 102.4697(6) |
| *V*, Å ^3^ | 1263.95(13) |
| *Z* | 2 |
| ρ_calcd_, g/cm^3^ | 1.921 |
| µ, mm^–1^ | 1.746 |
| *F*(000) | 724 |
| θ Range, deg | 2.1-27.6 |
| Reflection Collected | 5382 |
| Independent reflections (*R*_int_) | 0.018 |
| Reflections with *I* > 2σ(*I*) | 4873 |
| Number of parameters | 357 |
| *R*_1_, *wR*_2_ (*I* > 2σ(*I*))^*^ | 0.0362, 0.0974 |
| *R*_1_, *wR*_2_ (all data)^**^ | 0.0401, 0.1006 |

* *R* = ∑(*F*_o_ – *F*_c_)/∑(F_o_), ** *wR*_2_ = {∑[*w*(*F*_o_^2^ – *F*_c_^2^)^2^]/∑(*F*_o_^2^)^2^}^1/2^.

**Table S2.** Selected bond distances (Å) and angles (deg) for **1**

| **1** | | | | | |
| --- | --- | --- | --- | --- | --- |
| Cd(1)-O(1) | 2.414(3) | Cd(1)-O(1W) | | | 2.309(4) |
| Cd(1)-O(2) | 2.371(4) | Cd(1)-O(7) | | | 2.307(17) |
| Cd(1)-N(1) | 2.243(7) | Cd(1)-O(5)#1 | | | 2.243(3) |
| Cd(2)-O(7) | 2.75(2) | Cd(2)-O(8) | | | 2.288(9) |
| Cd(2)-N(3) | 2.217(4) | Cd(2)-O(6)#1 | | | 2.415(3) |
| Cd(2)-O(3)#2 | 2.512(4) | Cd(2)-O(4)#2 | | | 2.225(3) |
|  | | | | | |
| **1** | | | | | |
| O(1)-Cd(1)-O(1W) | 91.34(14) | | O(1)-Cd(1)-O(2) | 54.56(12) | |
| O(1)-Cd(1)-O(7) | 99.6(5) | | O(1)-Cd(1)-N(1) | 89.04(11) | |
| O(1W)-Cd(1)-O(2) | 93.98(14) | | O(1W)-Cd(1)-O(7) | 168.3(5) | |
| O(1W)-Cd(1)-N(1) | 91.90(14) | | O(2)-Cd(1)-O(7) | 89.1(5) | |
| O(2)-Cd(1)-N(1) | 143.20(12) | | O(7)-Cd(1)-N(1) | 92.4(5) | |
| O(7)-Cd(2)-O(8) | 49.1(4) | | O(7)-Cd(2)-N(3) | 97.1(4) | |
| O(8)-Cd(2)-N(3) | 91.1(2) | | O(6)#1-Cd(2)-N(3) | 87.37(13) | |

Symmetry Cddes: **For 1**: #1 = x, 1+y, z; #2 = 1+x, 1+y, z.

**Table S3** Comparison of the selected materials in detective sensitivity for TNP ions (DMF solvent)

| Material | *K*_sv_ | Sensitivity | Reference |
| --- | --- | --- | --- |
| [Eu_2_(L)_2_(DMAC)_2_]·nH_2_O | 3.58 × 10^4^ M^−1^ | 4.66 × 10^−4^ mM | 1 |
| {[Cd_4_(L)_2_(L2)_3_(H_2_O)_2_](8DMF)(8H_2_O)} | 3.89 × 10^4^ M^−1^ | ~ | 2 |
| [Zn_2_(TPOM)(NH_2_–BDC)_2_]·4H2O | 4.60 × 10^4^ M^−1^ | 0.98 mM | 3 |
| Zr_6_O4(OH)_8_(H_2_O)_4_(TTNA)_8/3_ | 5.1 × 10^5^ M^−1^ | 10 ppb | 4 |
| UiO-67@N | 2.9 × 10^4^ M^−1^ | ~ | 5 |
| [Cd(NDC)_0.5_ (PCA)] | 3.5 × 10^4^ M^−1^ | 1.55 mM | 6 |
| Zn-PDA | 4.5 × 10^4^ M^−1^ | 1.15 mM | 7 |
| poly(silafluo rene-vinylene) | 2.0 × 10^4^ M^−1^ | ~ | 8 |
| PCPC-MS | 1.5 × 10^4^ M^−1^ | ~ | 9 |
| TAPB | 1.2 × 10^4^ M^−1^ | 2.88 ppm | 10 |
| **1** | 5.42 × 10^4^ M^−1^ | 1.77 ppm | This work |

1. X. Y. Wang, P. F. Yan, Y. X. Li, G. H. An, X. Yao and G. M. Li, Cryst. Growth. Des., 2017, 17, 2178.

2. T. K. Pal, C. Nabanita and P. K. Bharadwaj, Inorg. Chem., 2016, 55, 1741.

3. R. Lv, J. Y. Wang, Y. P. Zhang, H.Li, L. Y. Yang, S. Y. Liao, W. Gu and X. Liu*, J. Mater. Chem. A., 2016, 4, 15494.

4. B. Wang, X. L. Lv, D. W. Feng, L. H. Xie, J. Zhang, M. Li, Y. B. Xie, J. R. Li and H. C. Zhou, J. Am. Chem. Soc., 2016, 138, 6204.

5. S. S. Nagarkar, A. V. Desai and S. K. Ghosh, Chem. Commun., 2014, 50, 8915.

6. S. S. Nagarkar, B. Joarder, A. K. Chaudhari, S. Mukherjee and S. K. Ghosh, Angew. Chem. Int. Ed., 2013, 52, 2881.

7. X. Jiang, Y. Liu, P. Wu, L. Wang, Q. Wang, G. Zhu, X. L. Li and J. Wang, RSC. Adv., 2014, 4, 47357.

8. J. C. Sanchez, A. G. DiPasquale, A. L. Rheingold and W. C. Trogler, Chem. Mater., 2007, 19, 6459.

9. W. Wei, R. Lu, S. Tang and X. J. Liu, Mater. Chem. A., 2015, 3, 4604.

10. P. Vishnoi, M. G. Walawalkar, S. Sen, A. Datta, G. N. Patwari and R. Murugavel, Phys. Chem. Chem. Phys., 2014, 16, 10651.

**Table S4** Comparison of the selected materials in detective sensitivity for Fe^3+^ ions (water solvent as medium)

| Material | Sensitivity | Reference |
| --- | --- | --- |
| Eu(acac)_3_@Zn(C_15_H_12_NO_2_)_2_ | 5×10^-3^ M | 1 |
| Eu(C_33_H_24_O_12_)(H_2_NMe)(H_2_O) | 2×10^-4^ M | 2 |
| Eu(C_22_H_14_O_2_)_3_ | 10^-4^ M | 3 |
| [Eu(BTPCA)(H_2_O)]·2DMF·3H_2_O | 10^-5^ M | 4 |
| MIL-53(Al) | 0.9×10^-6^ M | 5 |
| {[LnCd_2_(DTPA)_2_(H_2_O)_4_]·4H_2_O | 1.5×10^-5^ M | 6 |
| carbon nanoparticles (CNPs) | 0.32×10^-6^ M | 7 |
| Fluorescent Gold Nanoclusters | 5.4×10^-6^ M | 8 |
| [Cd_3_(dpa)(DMF)_2_(H_2_O)_3_]·DMF | 1.75×10^-4^ M | 9 |
| Zn_3_L_3_(DMF)_2_ | 10^-5^ M | 10 |
| [[Eu_2_(MFDA)_2_(HCOO)_2_(H_2_O)_6_]·H_2_O | 1.0×10^-4^ M | 11 |
| [Tb_4_(OH)_4_(DSOA)_2_(H_2_O)_8_]·(H_2_O)_8_ | 10^-6^ M | 12 |
| [H_2_N(Me)_2_][Eu_3_(OH)(bpt)_3_(H_2_O)_3_] (DMF)_2_·(H_2_O)_4_ | 10^-5^ M | 13 |
| [Eu_2_(MFDA)_2_(HCOO)_2_(H_2_O)_6_]·H_2_O | 10^-5^ M | 14 |
| TbL | 10^-6^ M | 15 |
| [Eu(HL)(H_2_O_2_)]·2H_2_O | 3.0×10^-4^ M | 16 |
| **1** | 2.8×10^-5^ M | *In this work* |

**Table. S5 The ICP result of filtrate.**

_____________________________________________________________________

Elem Units Avg

Cd mg/L 0.0109

Fe mg/L 0.3116

**References:**

1. G. G. Hou, Y. Liu, Q. K. Liu, J. P. Ma and Y. B. Dong, *Chem. Commun.*2011, ***47***, 10731-10733.
2. S. Dang, E. Ma, Z.M. Sun and H. J. Zhang, *J. Mater. Chem.* 2012, ***22***, 16920-16926.
3. M. Zheng, H. Q. Tan, Z. G. Xie, L. G. Zhang, X. B. Jing and Z. C. Sun, *ACS Appl. Mater. Interfaces,* 2013, ***5***, 1078-1083.
4. Q. Tang, S. X. Liu, Y. W. Liu, J. Miao, S. J. Li, L. Zhang, Z. Shi and Z. P. Zheng, *Inorg. Chem.*2013, ***52***, 2799-2801.
5. C. X. Yang, H. B. Ren and X. P. Yan, *Anal. Chem.*2013, ***85***, 7441-7446.
6. Q. Liu, F. Wan, L. X. Qiu, Y. Q. Sun and Y. P. Chen, *RSC Adv*., 2014, ***4***, 27013-27021.
7. K. G. Qu, J. S. Wang, J. S. Ren and X. G. Qu, *Chem. Eur. J.* 2013, ***19***, 7243-7249.
8. J.-A. A. Ho, H.-C. Chang and W.-T. Su, *Anal. Chem*. 2012, ***84***, 3246-3253.
9. J. C. Jin, L. Y. Pang, G. P. Yang, L. Hou and Y. Y. Wang, *Dalton Trans*., 2015, ***44***, 17222–17228.
10. Z. C. Yu, F. Q. Wang, X. Y. Lin, C. M. Wang, Y. Y. Fu, X. J. Wang, Y. N. Zhao and G. D. Li, *J. Solid. State. Chem*., 2015, ***232***, 96-101.
11. X. H. Zhou, L. Li, H. H. Li, T. Yang and W. Huang, *Dalton Trans*., 2013, **42**, 12403–12409.
12. X. Y. Dong, R. Wang, J. Z. Wang, S. Q. Zang and T. C. W. Mak, *J. Mater. Chem*. A, 2015, ***3***, 641–647.
13. S. Xing, Q. Bing, L. Song, G. Li, J. Liu, Z. Shi, S. Feng and R. Xu, *Chem. – Eur. J.*, 2016, **22**, 16230-16235.

[14] X. Zhou, L. Li, H. Li, A. Li, T. Yang and W. Huang, *Dalton Trans.,* 2013, **42**, 12403-12409.

[15] S. Dang, T. Wang, F. Yi, Q. Liu, W. Yang and Z. Sun, *Chem. – Asian J.*, 2015, **10**, 1703-1709.

[16] Y. Liang, G. Yang, B. Liu, Y. Yan, Z. Xi and Y. Wang, *Dalton Trans.,* 2015, **44**, 13325-13330.
